# Structural basis and physiological significance of non-canonical G_s_ coupling to the melatonin MT_1_ receptor

**DOI:** 10.1038/s41467-026-73555-6

**Published:** 2026-05-21

**Authors:** Atsuro Oishi, Hiroyuki H. Okamoto, Keisuke Ikegami, Ronan McHugh, Bernard Masri, Tsukasa Kusakizako, Kazuhiro Kobayashi, Akifumi Takaki, Angeliki Karamitri, Erika Cecon, Julie Dam, Miki Nagase, Irina G. Tikhonova, Osamu Nureki, Ralf Jockers

**Affiliations:** 1 Université Paris Cité, Institut Cochin, INSERM, CNRS, 75014 Paris, France; 2 Department of Anatomy, Kyorin University School of Medicine, Mitaka, Tokyo Japan; 3 Department of Biological Sciences, Graduate School of Science, The University of Tokyo, Bunkyo-ku, Tokyo Japan; 4 Department of Physiology, School of Medicine, Aichi Medical University, Nagakute, Aichi Japan; 5 Laboratory of Regulation in Metabolism and Behavior, Faculty of Agriculture, Kyushu University, Fukuoka, Japan; 6 School of Pharmacy, Queen’s University Belfast, Belfast, UK

**Keywords:** Cryoelectron microscopy, Neurophysiology, Melatonin, G protein-coupled receptors

## Abstract

G protein-coupled receptors (GPCRs) transduce extracellular stimuli into intracellular signals by coupling to various heterotrimeric G proteins. However, the rules governing G protein preference remain largely elusive. MT_1_ and MT_2_ are prototypical G_i/o_-coupled GPCRs responding to melatonin, a hormone secreted in a circadian manner. We show here that MT_1_, but not MT_2_, couples also to G_s_ proteins in vitro and activates the G_s_/cAMP pathway upon long-term melatonin exposure in vivo, mimicking physiological dawn conditions. We solve the cryo–electron microscopy structure of the melatonin-MT_1_-G_s_ complex at 3.0 Å resolution, which reveals a distinct binding mode compared to the MT_1_–G_i_ complex. The third intracellular loop of MT_1_ emerges as a key stabilizer for G_s_ coupling. This structure of a GPCR primarily coupling to G_i_, here in complex with G_s_, provides structural and functional insights into G protein selectivity and circadian switch of G protein coupling.

## Abstract (web summary)

How GPCRs choose between different G proteins remains unclear. Here, authors show that the prototypical Gi-coupled melatonin receptor MT_1_ also couples to G_s_, resolve the MT_1_-G_s_ structure, and identify ICL3 as a key determinant of this coupling.

## Introduction

G protein-coupled receptors (GPCRs) represent the largest family of membrane proteins involved in cellular signal transduction. GPCRs activate their primary transducers, heterotrimeric G proteins that are classified in four families: G_s_, G_i/o_, G_q/11_, and G_12/13_. Among those, G_s_ and G_i/o_ proteins have opposing roles as they are stimulating or inhibiting adenylyl cyclases and cyclic AMP production, respectively. Large-scale common G protein coupling datasets show that a particular GPCR can couple to one or several G protein types^1^. G_s_-coupled GPCRs show the lowest coupling promiscuity and rarely co-couple to G_i/o_ proteins (e.g., V2R) or with a large difference in their strength of activation (e.g., β1AR)^1^. The few exceptions of G_s_/G_i/o_ co-coupling include the primarily G_s_-coupled 5-HT4, EP4, and ß1AR^2–4^, as well as the primarily G_i/o_-coupled GPR139^5^.

MT_1_ and MT_2_ are high-affinity receptors for melatonin (5-methoxy-N-acetyl-tryptamine) that primarily couple to G_i/o_ proteins, leading to inhibition of cAMP production and the PKA/CREB pathway, activation of ERK1/2, and regulation of ion channels^6–8^. The molecular structures of MT_1_ and MT_2_ receptors have been solved, all in the presence of synthetic agonists, with some in complex with the G_i_ protein^8–13^. The stimulation profile of melatonin receptors is unique, governed by the circadian secretion pattern of melatonin in the pineal gland with peak levels during the night^14^. Due to this rhythmic pattern of melatonin secretion, melatonin receptors regulate and synchronize a variety of physiological functions, including sleep/awake rhythm, seasonal reproduction, and retina physiology, with currently marketed drugs indicated for sleep and circadian disorders and major depression^15,16^. Whereas most effects of melatonin receptors appear to be transmitted by G_i/o_ proteins, coupling to G_s_-dependent pathways has been suggested for MT_1_^17–19^. However, whether this is due to direct G_s_ coupling or to downstream crosstalk is unknown. The physiological relevance of dual G_i_/G_s_ coupling, with apparently opposing signaling outcomes, also remains elusive and intriguing.

In this work, we provide functional evidence for G_s_ coupling to MT_1_ under physiologically relevant conditions in the mouse hypophysial pars tuberalis (PT), and report the cryo-electron microscopy (cryo-EM) structure of the MT_1_–G_s_ complex in the presence of the natural ligand melatonin. The unique interaction mode of G_s_ depends on the third intracellular loop of MT_1_ (ICL3), which is required to stabilize the interaction with MT_1_.

## Results

### Melatonin MT_1_ receptor activates both the inhibitory G_i_/cAMP and the stimulatory G_s_/cAMP pathway

**Fig. 1:**
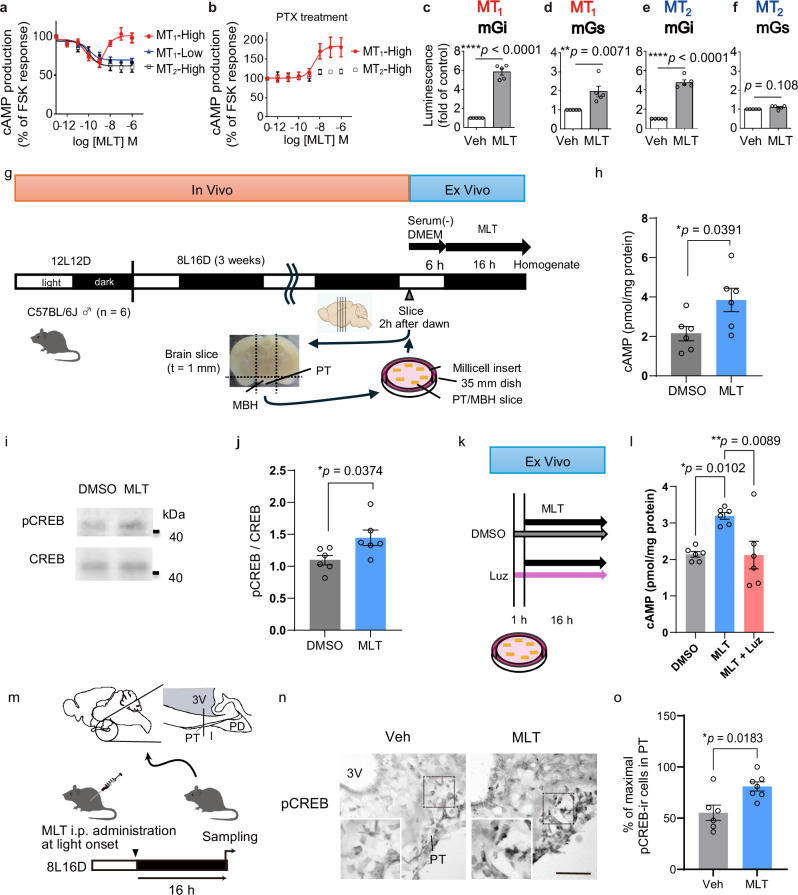
Melatonin MT_1_ receptor activates the stimulatory G_s_/cAMP pathway in vitro, ex vivo, and in vivo. **a**–**f** Melatonin MT_1_ receptor activated G_s_-cAMP pathway in HEK293T cells. Concentration–response effect of melatonin (MLT) on forskolin (FSK)-induced cAMP production (30 min, FSK [2 µM], IBMX [1 mM]) in HEK293T cells expressing MT_1_-WT or MT_2_-WT, assessed by HTRF without (**a**) or with (**b**) PTX treatment (400 ng/mL, 4 h). Data are means ± SEM from independent experiments ((**a**) *n* = 6; (**b**) MT_1_, *n* = 5; MT_2_, *n* = 4). NanoBiT complementation assay showing recruitment of LgBiT–miniG_i_ (**c**, **e**) or LgBiT–miniG_s_ (**d**, **f**) to human MT_1_-NP (**c**, **d**) or MT_2_-NP (**e**, **f**) upon melatonin stimulation (1 µM, 2 min). Data are means ± SEM from five independent experiments (*n* = 5). Two-tailed ratio-paired t-tests were used; exact *p*-values are reported. **g**–**l** Melatonin induces cAMP production in mouse pituitary pars tuberalis (PT) ex vivo. **g** Melatonin exposure (10 µM) of sliced PT/mediobasal hypothalamus (MBH) tissue complexes from C57BL/6J mice (*n* = 6) kept under 8 L/16 D conditions. **h** Sixteen-hour melatonin exposure increased PT/MBH cAMP levels (**p = 0.0391, Welch’s two-tailed unpaired t-test), and phosphorylated CREB (pCREB [Ser133]) (** *p* = 0.0374, Welch’s two-tailed unpaired t-test). Data are means ± SEM. **k**, **l** One-hour preincubation with MT_1/2_ antagonist luzindole (Luz) prevented the MLT-induced cAMP elevation (**p = 0.0089 vs MLT with Dunnett’s multiple-comparisons test). **m**–**o** Melatonin activates CREB in mouse pituitary PT in vivo. **m** MLT (0.26 mM/0.1 mL) was administered intraperitoneally (i.p.) at light offset under 8L16D, and brains were collected 16 h later for histological analyses. 3 V, Third ventricle. PD pars distalis. **n** Representative pCREB immunoreactivity (ir) images in the PT and MBH of Veh and MLT-injected mice. Scale bar = 100 μm. **o** Relative changes of pCREB-ir cells normalized by area in the PT. Melatonin increased pCREB (**p = 0.0183, Welch’s two-tailed unpaired t-test). Data are means ± SEM (Veh; *n* = 6, MLT; *n* = 7). Shapiro–Wilk normality/log-normality assessments (all *p* > 0.05) and *t*/*F*/d*f* values for all panels are presented in Supplementary Fig. 3. Source data are provided as a Source data file.

Acute stimulation with melatonin of HEK293 cells expressing low levels of human MT_1_ receptor inhibited forskolin (FSK)-stimulated cAMP production with a pEC_50_ of 9.96 ± 0.34 (*n* = 6), as expected for this prototypical G_i/o_-coupled receptor (Fig. 1a). In contrast, cells expressing high MT_1_ levels showed a biphasic behavior with inhibition of cAMP production at low melatonin concentrations (pEC_50_ = 10.3 ± 0.35; *n* = 6) and stimulation of cAMP production at higher melatonin concentrations (pEC_50_ = 8.6 ± 0.66; *n* = 6) (Fig. 1a). Similar experiments in cells expressing high levels of the closely related G_i/o_-coupled human MT_2_ melatonin receptor showed only the inhibitory component (pEC_50_ = 9.93 ± 0.23; *n* = 6), indicating that the biphasic behavior is a unique feature of MT_1_.

Pretreatment of HEK293 cells expressing high levels of MT_1_ and MT_2_ with pertussis toxin (PTX), a G_i/o_ inhibitor, abolished the inhibitory effect of melatonin for both receptors, while the stimulatory effect was preserved for MT_1_ with a pEC_50_ of 8.51 ± 0.38 (*n* = 5) (Fig. 1b), which was similar to the value observed in the absence of PTX. This indicates that MT_1_ and MT_2_ couple to PTX-sensitive G_i/o_ proteins and MT_1_ to an additional PTX-insensitive G protein, possibly the stimulatory G_s_ protein. To test this hypothesis, we measured G_i_ and G_s_ coupling directly to MT_1_ and MT_2_ with the nanoluciferase (Nluc) complementation assay^20,21^. MT_1_ and MT_2_ were fused at their C-terminus to the NP fragment of Nluc (MT_1(2)_-NP) and coexpressed with miniGα_s_ or miniGα_i_ proteins fused to the LgBiT fragment of Nluc (LgBit-miniGα_s/i_) to monitor G protein recruitment through Nluc complementation^16,17^. First, we recapitulated the bi- vs. monophasic melatonin concentration–response curves of FSK-stimulated cAMP production with the NP-tagged human and mouse MT_1_ and MT_2_ (Supplementary Fig. 1a–d). In the Nluc complementation assay, melatonin-promoted LgBit-miniGα_i_ recruitment was observed with both receptors (Fig. 1c, e) and between LgBit-miniGα_s_ and MT_1_-NP, but not MT_2_-NP (Fig. 1d, f), indicating that MT_1_ couples to G_i_ and G_s_ while MT_2_ couples only to G_i_. Melatonin-induced G protein profiles were replicated in complementary BRET assays (Supplementary Fig. 2a–d). In addition, BRET assays did not provide evidence for constitutive G_s_ coupling to MT_2_, which was readily detectable for GPR62, which has been reported to constitutively activate the G_s_/cAMP pathway^22^ (Supplementary Fig. 2d–f).

MT_1_ is abundantly expressed in the rodent pituitary PT, whereas MT_2_ is absent^23,24^. Acute melatonin treatment has been shown to inhibit FSK-stimulated cAMP production in cultured PT cells^25^. To simulate physiologically relevant long-term stimulation conditions, we first established ex vivo cultures from PT of melatonin-deficient C57BL/6 mice kept under short-day conditions (8 h light: 16 h dark) and stimulated them for 16 h with melatonin (Fig. 1g). After assessing the data normality (Supplementary Fig. 3), we found that, under these conditions, melatonin promoted cAMP production (Fig. 1h) and further downstream CREB phosphorylation (Fig. 1i, j, Supplementary Figs. 3 and 5), which are conserved in C57BL/6 mice kept under different light conditions (12 h light: 12 h dark) (Supplementary Fig. 4a–c) and in melatonin-proficient CBA/N mice (Supplementary Fig. 4d–f), indicating a minor contribution of the endogenous melatonin rhythm and circadian timing on the responses. To confirm the importance of MT_1_ in these responses, we applied the MT_1/2_ antagonist luzindole, which suppressed, as expected, the melatonin-induced cAMP elevation (Fig. 1k, l). Since these PT slices include the hypothalamus and the response was observed only ex vivo, we next verified whether this pathway responds specifically in the PT in vivo. Intraperitoneal administration of melatonin to mice kept under short-day conditions increased CREB phosphorylation in the PT 16 h after treatment (Fig. 1m–o and Supplementary Fig. 3). Collectively, these data indicate that endogenously expressed MT_1_ couples to the G_s_/cAMP pathway in vivo when stimulated for 16 h, mimicking the end of the dark phase under short-day conditions.

### Structural insights into the MT_1_-miniG_s_ signaling complex

**Fig. 2:**
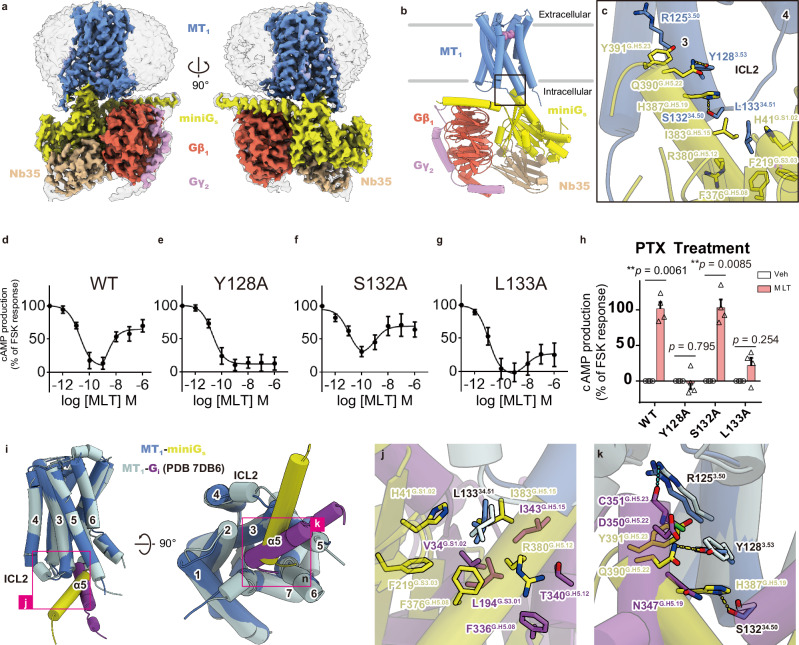
Cryo-EM structure of the MT_1_-miniG_s_ complex. **a** CryoEM density map of MT_1_-miniG_s_ with Gβ_1_, Gγ_2_, and Nb35. MT_1_, miniG_s_, Gβ_1_, Gγ_2_, and Nb35 were shown as blue, yellow, red, pink, and brown, respectively. Map including micelle density was shown in a transparent style. **b** Overall structure of MT_1_-miniG_s_ with Gβ_1_, Gγ_2_, and Nb35. The melatonin molecule is shown as a light purple CPK model. **c** The interface between MT_1_ and miniG_s_. Concentration–response effect of melatonin (MLT) on FSK-induced cAMP production (5 min, FSK [5 µM]) in HEK293T cells expressing MT_1_-WT (**d**) or mutant receptors Y128A (**e**), S132A (**f**), and L133A (**g**), monitored with the BRET-based CAMYEL biosensor. Data represent means ± SEM from six independent experiments. **h** Melatonin (1 µM, 10 min)-induced cAMP production in the absence of FSK in HEK293T cells pretreated with PTX (400 ng/mL, 4 h) and expressing MT1-WT or mutant receptors (Y128A, S132A, and L133A) assessed with the BRET-based CAMYEL biosensor. Data represent means ± SEM from four independent experiments (*n* = 4). Multiple paired two-tailed t-tests with Holm–Šidák correction were used for statistical comparison; exact adjusted *p*-values are indicated. Shapiro–Wilk normality assessments (all *p* > 0.05) and *t*/*F*/d*f* values for all panels are presented in Supplementary Fig. 3. Structural comparison between MT_1_-miniG_s_ (light blue) and MT_1_–G_i_ (purple; PDB 7DB6), displaying **i** overall structure, **j** ICL2, and **k** TM3. Source data are provided as a Source data file.

To obtain structural evidence for G_s_ coupling to MT_1_, we determined the structure of the MT_1_-miniG_s_ complex by cryo-EM at 3.0 Å global resolution, together with the endogenous ligand melatonin, Gβ_1_, Gγ_2_ subunits, and the Nb35 fragment (Fig. 2a, b, and Supplementary Fig. 6a, b). The overall structure of MT_1_ is similar to the MT_1_ structure in the previously reported MT_1_–G_i_ complex^12^. In the MT_1_-miniG_s_ complex, MT_1_ is in an active state, supported by the structural similarity at the conserved activation motifs within the MT_1_–Gi complex (Supplementary Fig. 7a–e). At the G_s_ interface, the MT_1_-miniG_s_ displayed a distinctive binding mode, where Arg125^3.50^, in the DRY motif of MT_1_ made no interaction with Tyr391^G.H.5.23^ of G_s_, while this interaction is observed in almost all of the reported structures of class A GPCR-G_s_ complex (Fig. 2c). Instead of the typical interaction between Arg^3.50^ and Tyr391^G.H.5.23^, Tyr128^3.53^ at TM3 of MT_1_ formed a hydrogen bond with Gln390^G.H.5.22^ of G_s_, and Ser132^34.50^ at intracellular loop 2 (ICL2) of MT_1_ formed a hydrogen bond with His387^G.H.5.19^ of G_s_. In parallel, Leu133^34.51^ at ICL2 of MT_1_ formed a hydrophobic interaction with the pocket of miniG_s_ formed by His41^G.S1.02^, Phe219^G.S3.03^, Phe376^G.H5.08^, Arg380^G.H5.12^, and Ile383^G.H5.15^, which is observed in most of the G_s_ complexes (Fig. 2c).

The importance of these interacting residues was tested by the real-time cAMP assay with the Y128^3.53^A, S132^34.50^A, and L133^34.51^A MT_1_ mutants (Fig. 2d–h). All mutated receptor variants showed similar expression levels (Supplementary Fig. 8). Whereas the inhibitory G_i_-dependent phase was maintained for all mutants, the stimulatory G_s_-dependent phase was completely lost for Y128^3.53^A, not affected for S132^34.50^A, and substantially reduced for L133^34.51^A. This suggests the importance of Tyr128^3.53^ and, to a lesser extent, Ser132^34.50^ in cAMP production via MT_1_–G_s_ coupling, while maintaining MT_1_–G_i_ coupling.

Despite these unique binding modes at the MT_1_-miniG_s_ interface, miniG_s_ in the MT_1_-miniG_s_ showed a similar conformation as the previously reported β_2_AR-G_s_ complex (PDB 3SN6) and was distinct from the GDP-bound inactive G_s_ protein structure (PDB 6EG8) (Supplementary Fig. 7k–m). Phe376^G.H5.08^ moved upward to the C-terminal of the α5 helix in a typical way, causing the extension of the α5 helix together with the movement of the TCAT motif at the nucleotide binding site (Supplementary Fig. 7k–m). From these observations, we concluded that the MT_1_-miniG_s_ complex is a canonical signaling complex rather than an artifact.

Structural comparison with the previous structure of MT_1_ in complex with its primary signal transducer, the G_i_ trimer (PDB 7DB6), revealed that MT_1_ exhibits a distinct binding mode when bound to miniG_s_ (Fig. 2i). While the overall structure of MT_1_ remains similar between complexes, the G protein α5 helixes adopt dramatically different entry angles: G_s_ α5 inserts from the ICL2 side, whereas G_i_ α5 penetrates from the ICL3 side (Fig. 2i). This differential positioning enables unique interactions in each complex. In the MT_1_–G_s_ structure, Leu133^34.51^ forms hydrophobic contacts with the Gα pocket - an interaction absent in the MT_1_–G_i_ complex (Fig. 2j, k). This observation aligns with preserved cAMP inhibition in L133^34.51^A and Y128^3.53^A mutants. Conversely, G_i_ residues at equivalent positions do not form similar interactions between Cys351^G.H.5.23^ of G_i_ and Arg125^3.50^ of MT_1_ (Fig. 2k). Critically, G_s_ α5 adopts shallow binding with its C-terminal tip oriented toward ICL2/TM3, while G_i_ α5 penetrates more deeply toward TM5/TM6 regions^12^. We conclude from these observations that the binding mode of the MT_1_–G_s_ complex is distinctive from that of the MT_1_–G_i_ complex.

### MT_1_ shares a similar G_s_ entry angle with GPR61 but shows a different binding mode from GPR61

**Fig. 3:**
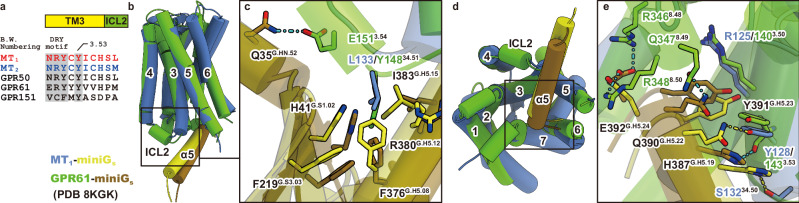
Structural comparison with the GPR61-G_s_ complex. **a** Amino acid sequences alignment of human MT_1_, MT_2_, GPR50, GPR61, and GPR151. Structural comparison between MT_1_-miniG_s_ and GPR61-miniG_s_ (PDB 8KGK) displaying **b** overall, **c** ICL2 interface, **d** overall structure from cytoplasmic side, and **e** α5 helix interface.

To identify other GPCRs coupling to G_s_ in a similar way to MT_1_, we performed an amino acid sequence alignment of TM3 and ICL2 of class A GPCRs using the GPCRdb server^26^. Surprisingly, only 5 class A GPCRs contain a Tyr at position 3.53, including MT_1_ and MT_2_ (Fig. 3a). Among the 5 GPCRs, GPR61 is the only receptor for which the structure of the G_s_ complex is available in PDB. Structural comparison with the recent cryo-EM structure of the GPR61-G_s_ complex (PDB 8KGK) revealed that MT_1_ and GPR61 share a similar G_s_ entry angle (Fig. 3b, d). However, G_s_ enters deeper into GPR61 than into MT_1_ both at ICL2 and the cytoplasmic pocket of the receptor (Fig. 3c, e). At the ICL2 interface, residues around Tyr148^34.51^ of GPR61 make closer contacts than MT_1_, and Glu151^3.54^ of GPR61 interacts with G_s_ via Gln35^G.HN.52^, which is not observed in the MT_1_-miniG_s_ structure (Fig. 3c). At the cytoplasmic pocket, the backbone of Tyr143^3.53^ of GPR61 forms a hydrogen bond with His387^G.H.5.19^ of G_s_, while Tyr128^3.53^ of MT_1_ forms a hydrogen bond with Gln390^G.H.5.22^ of G_s_ (Fig. 3e). Associated with this conformational difference, Pro147^34.50^ of GPR61 does not interact with G_s_, which is at the same position as Ser132^34.50^ of MT_1_ interacting with His387^G.H.5.19^ of G_s_. As a result, G_s_ enters deeper into GPR61 than MT_1_, associated with additional interactions; Gln347^8.49^ to Gln390^G.H.5.22^, and Arg346^8.48^ and Arg348^8.50^ to Glu392^G.H.5.24^. These structural insights may reflect the difference in G protein coupling between primary constitutive G_s_ signaling (of GPR61) and secondary G_s_ signaling (of MT_1,_ primarily coupling to G_i_).

We also compared the structures of five GPCRs in complex with both G_s_- and the G_i_-trimers with the MT_1_-miniG_s_ (Supplementary Fig. 9). Among these receptors, MT_1_ is unique as a primarily G_i_-coupled GPCR also complexed with G_s_. 5HT4-G_s_/-G_i_ displayed conformational differences in the α5 helix (Supplementary Fig. 9a), a pattern closely resembling MT_1_–G_s_/-G_i_ complexes (Fig. 2i). In contrast, the other four GPCRs-G_s_/-G_i_ complexes exhibited less pronounced differences in this region (Supplementary Fig. 9a, b). These comparisons implied there are several patterns of -G_s_/-G_i_ coupling to the same GPCR; however, identifying common structural features—including those involving the C-terminal regions of Gα—will likely require the accumulation of additional structural data for multiple GPCR–Gα binding modes.

### Structural basis of MT_1_ subtype-specific G_s_-coupling is in the TM5-ICL3-TM6 region

**Fig. 4:**
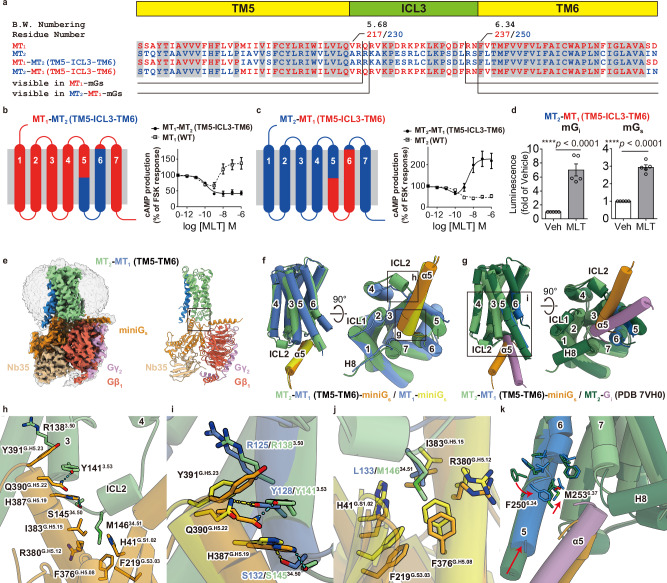
Structural basis of MT_2_-MT_1_-chimera G_s_ coupling. **a** Amino acid sequence alignment of human MT_1_ and MT_2_ chimeric receptors in the TM5-ICL3-TM6 region. In sequence alignments and schematics, red and blue indicate MT_1_- and MT_2_-derived regions, respectively. Concentration–response effect of melatonin on FSK-induced cAMP production (5 min, FSK [5 µM]) in HEK293T cells expressing **b** MT_1_–MT_2_(TM5-ICL3-TM6) or **c** MT_2_-MT_1_(TM5-ICL3-TM6) chimeric receptors, assessed with the BRET-based CAMYEL biosensor. Data represent means ± SEM of six independent experiments (*n* = 6). **d** NanoBiT complementation assay showing recruitment of LgBiT–miniG_i_ (left) or LgBiT–miniG_s_ (right) to the MT_2_-MT_1_(TM5-ICL3-TM6)-NP receptor upon melatonin stimulation (1 µM, 2 min). Data represent means ± SEM of five independent experiments (*n* = 5). Statistical significance was determined using ratio-paired two-tailed t-tests; exact *p*-values are reported. Shapiro–Wilk log-normality assessments (all *p* > 0.05) and *t*/*F*/d*f* values for all panels are presented in Supplementary Fig. 3. **e** CryoEM density map (left) and overall structure (right) of MT_2_-MT_1_(TM5-TM6)-miniG_s_ with Gβ_1_, Gγ_2_, and Nb35. MT_2_, MT_1_(TM5-TM6), miniG_s_, Gβ_1_, Gγ_2_, and Nb35 were shown as light green, blue, orange, red, pink, and brown, respectively. Map including micelle density was shown in a transparent style. Structural comparison of MT_2_-MT_1_(TM5-TM6)-miniG_s_ with **f** MT_1_-miniG_s_ or **g** MT_2_–G_i_ (PDB 7VH0), including lateral and cytoplasmic viewpoints. MT_2_ in MT_2_–G_i_ is shown as light purple, and G_i_ in MT_2_–G_i_ is shown as green. **h** The interface between MT_2_-MT_1_(TM5-TM6) and miniG_s_ around TM3 and ICL2. Structural comparison between MT_2_-MT_1_(TM5-TM6)-miniG_s_ and MT_1_-miniG_s_ at **i** TM3 and α5 helix or **j** ICL2. **k** Structural comparison between MT_2_-MT_1_(TM5-TM6)-miniG_s_ and MT_2_–G_i_ at TM5, TM6 and α5 helix. Source data are provided as a Source data file.

Tyr128^3.53^ is a key feature of MT_1_ for G_s_ coupling, yet Tyr^3.53^ also exists in MT_2_, which does not couple to G_s_, suggesting that features other than Tyr^3.53^ explain the exclusive MT_1_ coupling to G_s_ and not MT_2_. Recent structural studies demonstrated that the distance from the latter half of TM5 to TM6 is the key for G_s_ and G_i_ selectivity of serotonin receptor subtypes, as swapping of the TM5-ICL3-TM6 domain between the G_s_-coupled 5HT_4_ and the G_i_-coupled 5HT_1A_ switched also their G protein coupling profiles^2^. Inspired by this study, we exchanged the TM5-ICL3-TM6 regions between MT_1_ and MT_2_ (Fig. 4a, b). The MT_1_–MT_2_(TM5-6) chimera lost the melatonin-induced cAMP elevation phase seen in wild-type MT_1_, whereas the MT_2_-MT_1_(TM5-6) chimera gained the cAMP elevation phase of the wild-type MT_1_ (Fig. 4c). The MT_2_-MT_1_(TM5-6) chimera coupled to both miniGα_i_ and miniGα_s_ proteins (Fig. 4d). These data indicate that the molecular determinants of G_s_ coupling are located in the TM5-ICL3-TM6 region of MT_1_ and are absent in MT_2_.

To better understand the binding mode of G_s_ to the MT_2_-MT_1_(TM5-6) chimera, we determined its cryo-EM structure in complex with miniG_s_^star^^27^ (Fig. 4e and Supplementary Fig. 10a, b). The overall structure of the MT_2_-MT_1_(TM5-6)-G_s_ complex was similar to that of the MT_1_–G_s_ complex (Fig. 4f), which represents an active receptor state. This is supported by the structural similarity of the conserved activation motifs (Supplementary Fig. 7f–j) and the specific motifs in G_s_ (Supplementary Fig. 7n–p). The only notable difference is a slight shift in the position of the α5 helix between the chimera complex and the MT_1_–G_s_ (Fig. 4f). Associated with this conformational change, Arg138^3.50^ formed a stacking interaction with Tyr391^G.H.5.23^ of G_s_, though Tyr141^3.53^ and Ser145^34.50^ kept interactions with Gln390^G.H.5.22^ and His387^G.H.5.19^ of G_s_, respectively (Fig. 4h, i). In parallel, Met146^34.51^ at the same position of Leu133^34.51^ maintained a hydrophobic interaction with the pocket of G_s_ (Fig. 4j). To understand why the substitution of TM5-ICL3-TM6 enabled MT_2_ to couple to G_s_, we compared the MT_2_-MT_1_(TM5-6)-G_s_ structure with the previously solved structure of MT_2_ in complex with G_i_ (Fig. 4g, k). Similar to MT_1_, different entry angles were observed between G_s_ and G_i_. No other substantial changes were found. The position of TM5 and TM6 was similar between MT_2_ and the MT_2_-MT_1_(TM5-6) chimera structures indicating that the positioning and the rotation of TM5 and TM6 are not responsible for G_s_ coupling of the chimera (Fig. 4k). Collectively, these data suggest that TM5 and TM6 do not play an important role in G_s_ coupling of MT_1_ vs MT_2_, and suggest that key molecular determinants are likely located within ICL3.

### ICL3 of MT_1_ supports coupling to G_s_ but not to G_i_

**Fig. 5:**
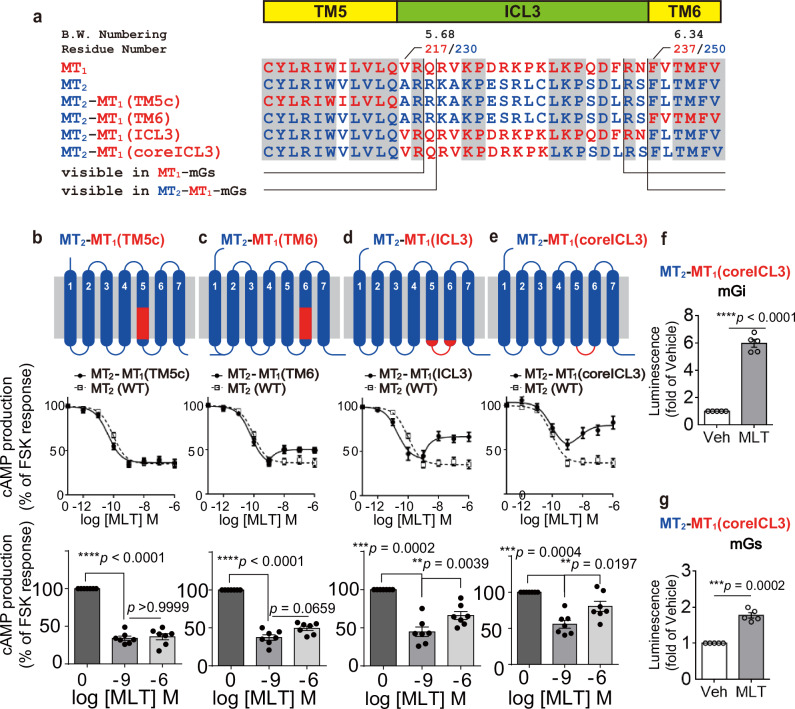
Identification of key determinants of MT_1_–G_s_ interaction by MT_2_-MT_1_ chimera receptors. **a** Amino acid sequences alignment of human MT_2_ chimeric receptors. In sequence alignments and schematics, red and blue indicate MT_1_- and MT_2_-derived regions, respectively. **b**–**e** (Upper) Schematic representation of a chimera. (Middle) Concentration–response effect of melatonin on FSK-induced cAMP production (10 min, FSK [5 µM]) in HEK293T cells expressing **b** MT_2_-MT_1_(TM5c), **c** MT_2_-MT_1_(TM6), **d** MT_2_-MT_1_(ICL3), or **e** MT_2_-MT_1_(core ICL3) chimeric receptors, assessed with the BRET-based CAMYEL biosensor. Data represent means ± SEM of seven independent experiments (*n* = 7). (Lower) Statistical comparison of concentration–response data at 0, 1 nM, and 1 µM of melatonin. Statistical analysis was performed by repeated-measures one-way ANOVA with Geisser–Greenhouse correction followed by Bonferroni’s multiple-comparisons test. Exact adjusted *p*-values are shown on the plots. NanoBiT complementation assay showing recruitment of LgBiT–miniG_i_ (**f**) or LgBiT–miniG_s_ (**g**) to the MT_2_-MT_1_(coreICL3)–NP receptor upon melatonin stimulation (1 µM, 2 min). Data represent means ± SEM of five independent experiments. Statistical significance was determined using ratio-paired two-tailed t-tests; exact *p*-values are reported. Shapiro–Wilk log-normality assessments (all *p* > 0.05) and *t*/*F*/d*f* values for all panels are presented in Supplementary Fig. 3. Source data are provided as a Source data file.

To provide experimental evidence for the importance of ICL3 over TM5 and TM6 of MT_1_ for G_s_ coupling, we generated MT_2_-MT_1_ chimera containing TM5, ICL3, or TM6 of MT_1_ alone or a combination of ICL3 and TM6 (Fig. 5a–e and Supplementary Fig. 11). All chimeras were expressed at comparable levels as the MT_2_-MT_1_(TM5c-ICL3-TM6) reference chimera (Supplementary Fig. 12). All chimeras showed the melatonin-induced cAMP inhibition phase (Fig. 5b–e and Supplementary Fig. 11). The MT_2_-MT_1_(ICL3) chimera displayed, in addition, the cAMP elevation phase (Fig. 5d). Among the 20 amino acids of the ICL3 region, the MT_2_-MT_1_(coreICL3) chimera containing the N-terminal 12 amino acids (VRQRVKPDRKPK) of MT_1_ maintained the G_s_ phase (Fig. 5e), whereas the MT_2_-MT_1_(C-ICL3 + TM6) chimera containing the 8 C-terminal amino acids did not contribute to the G_s_ phase (Supplementary Fig. 11b). Further delimitation of the 12 amino acid stretch failed to induce the G_s_ phase in the MT_2_ context (Supplementary Fig. 11c, d), defining the 12 N-terminal amino acids of the MT_1_-ICL3 as the minimal region for the G_s_ phase of cAMP signaling. Coupling of the minimal MT_2_-MT_1_(coreICL3) chimera to both G_s_ and G_i_ was confirmed directly in the miniG_s/i_ coupling assays (Fig. 5f, g).

**Fig. 6:**
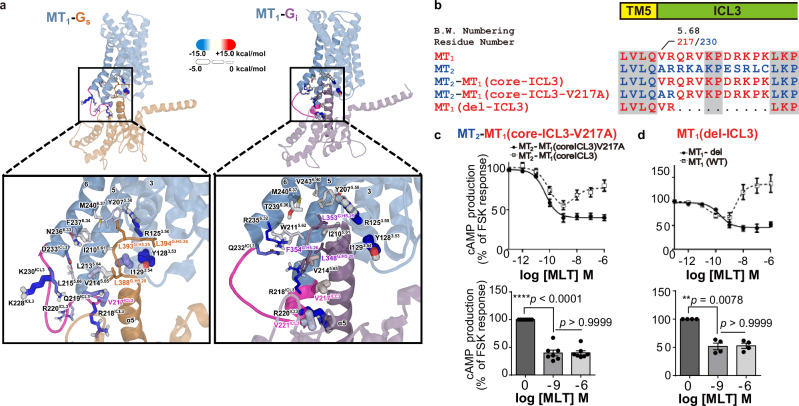
Molecular dynamics simulations support differential G protein binding modes and ICL3-dependent G_s_ coupling of MT_1_. **a** Representative MD simulation snapshot of MT_1_–G protein complexes. Carbon colors indicate electrostatic energies, stick thickness represents van der Waals interactions with the α5 helix. Only residues >1 kcal/mol in TM5-ICL3-TM6 are shown. AlphaFold3-modeled ICL3 (pink) highlights non-conserved hydrophobic residues. TM5 and TM6 are highlighted in a darker color. Key contacts: I210^5.61^ with L348^G.H5.20^, F354^GH5.26^, and L353^GH5.25^ (G_i_ deep binding) versus V217^ICL3^ with L388^G.H5.20^ (G_s_ shallow binding). **b** Amino acid sequence alignment of human MT_2_ chimera and human MT_1_ deletion mutant in the TM5-ICL3 region. Red and blue indicate MT_1_- and MT_2_-derived regions, respectively. (Upper) Concentration–response effect of melatonin on FSK-induced cAMP production (10 min, FSK [5 µM]) in HEK293T cells expressing **c** MT_2_-MT_1_(coreICL3-V217A) and **d** MT_1_-ICL3-deletion mutant chimeric receptors, assessed with the BRET-based CAMYEL biosensor. Data represent means ± SEM of seven (**c**) or four (**d**) independent experiments (*n* = 7 (**c**), *n* = 4 (**d**)). (Lower) Statistical comparison of concentration–response data (upper) at 0, 1 nM, and 1 µM of melatonin. Statistical analysis was performed by repeated-measures one-way ANOVA with Geisser–Greenhouse correction followed by Bonferroni’s multiple-comparisons test. Exact adjusted *p*-values are shown on the plots. Shapiro–Wilk log-normality assessments (all *p* > 0.05) and *t*/*F*/d*f* values for all panels are presented in Supplementary Fig. 3. Source data are provided as a Source data file.

Since the flexible ICL3 could not be fully resolved in our MT_1_–G_s_ structure, we performed molecular dynamics (MD) simulations of MT_1_–G_s_, MT_1_–G_i_, MT_2_–G_i_, and MT_2_-MT_1_(TM5-ICL3-TM6)-G_s_ complexes using AlphaFold3-modeled ICL3 structures (Supplementary Tables 1 and 2). An MT_2_–G_s_ test model was generated to understand why wild-type MT_2_ cannot stabilize G_s_ interactions. Unlike many GPCRs with extensive ICL3 regions (>100 residues), the melatonin receptors possess relatively short, 20-residue loops with high AlphaFold3 confidence (>60%). The AlphaFold models align well with our cryo-EM structures, particularly at the N-terminal ICL3 region, where the first five residues are experimentally resolved as an extended TM5 helix (Supplementary Fig. 13). The AlphaFold pLDDT confidence scores (65.17 for MT_1_; 73.62 for MT_2_) reflect inherent loop flexibility. To avoid bias from the starting conformation, we performed both conventional MD and Gaussian accelerated MD (GaMD) simulations. These approaches revealed through principal component analysis that ICL3 samples substantially larger conformational space (Supplementary Fig. 14). MD simulations revealed that the ICL3 forms dynamic polar and hydrophobic interactions with G protein α5 helices, with notable hydrophobic contacts between MT_1_ V217^ICL3^ and L388^G.H5.20^ of G_s_ (Fig. 6a). Together with I129^3.54^, V214^5.65^, and L213^5.64^, these residues create a hydrophobic patch that stabilizes the receptor-G_s_ interface. While V217^ICL3^ also interacts with G_i_, these contacts are less essential because G_i_ α5 penetrates deeper into TM5/TM6 regions, achieving stability through intrinsic hydrophobic interactions involving L348^G.H5.20^, F354^GH5.26^, and L353^GH5.25^ with I210^5.61^ and I129^3.54^ (Fig. 6b), whose alanine mutation substantially reduces G_i_ coupling^12^. To experimentally validate the predicted role of V217^ICL3^, we generated a V217A mutant in the MT_2_-MT_1_(coreICL3) chimera, substituting the MT_1_ valine with the corresponding alanine from MT_2_ (Fig. 6c). While the inhibitory phase was preserved, the stimulatory phase was abolished consistent with the important role of V217^ICL3^ for G_s_, but not G_i_, coupling (Fig. 6c). Deletion of ICL3 (ΔQRVKPDRKPK) in MT_1_ resulted in the loss of the melatonin-induced cAMP stimulation phase while maintaining the inhibition phase, further confirming the essential role of ICL3 for G_s_ coupling but not for G_i_ coupling to MT_1_ (Fig. 6d). This ICL3 deletion chimera also shows that V217^ICL3^ alone is not sufficient for G_s_ coupling, as this residue, along with the other residues involved in the hydrophobic patch (I129^3.54^, L213^5.64^, and V214^5.65^) are present in this chimera (Fig. 6b). This is consistent with the observation that chimeric replacement of TM5 alone is not sufficient to enable G_s_ coupling to MT_2_ (see Fig. 5b). Our MT_2_–G_s_ test model MD simulations support this mechanism, showing that equivalent alanine residues in MT_2_ ICL3 cannot provide adequate stabilization of G_s_, resulting in high ICL3 mobility with substantially reduced GPCR–G protein interaction energies (Supplementary Fig. 15a–c). Since VRQRVKPDRKPK represents the minimal sequence enabling notable MT_2_–G_s_ coupling, a specific ICL3 conformation appears also essential for establishing hydrophobic interactions necessary for G_s_ recognition. Our findings demonstrate that ICL3 is essential for G_s_ coupling but dispensable for G_i_ coupling, in a context where G_s_ coupling to MT_1_ relies heavily on ICL3 interactions, which likely compensates for its shallow binding mode.

## Discussion

We describe here the molecular determinants enabling G_s_ coupling to MT_1_, a prototypical G_i_-coupled GPCR. The binding mode of the MT_1_–G_s_ complex is structurally distinctive from the MT_1_–G_i_ coupling: while G_i_ binding involves the C-terminal end of TM5 and TM6 of MT_1_^12,13^, G_s_ forms essential interactions with TM3 (Y128^3.53^) and ICL2 (L133^34.51^) of MT_1_, which were experimentally validated. Since the flexible ICL3 could not be resolved in our MT_1_–G_s_ structure, we investigated its role through MT_2_-MT_1_ chimera studies and MD simulations. Chimera studies defined the N-terminal 12 amino acids (217-VRQRVKPDRKPK-228) of MT_1_-ICL3 as the minimal region required for G_s_ coupling with V217^ICL3^, establishing hydrophobic contacts participating in G_s_ recognition and stabilizing the otherwise shallow binding mode of G_s_ in terms of depth of penetration into the TM5/TM6 region. Consistent with the absence of G_s_ coupling to MT_2_, MD simulations revealed that the stabilizing role of ICL3 observed for MT_1_ is not present in MT_2_. While the ICL3 is essential for G_s_ coupling to MT_1_, it is dispensable for G_i_ coupling as demonstrated with the MT_1_-ICL3 deletion mutant.

The contribution of ICL3 in G protein coupling has been suggested previously by functional and bioinformatics studies^28–30^. On the other hand, for different serotonin receptor subtypes, the length of TM5 and TM6 has been suggested as an important determinant of G_s_-G_i_ selectivity^2^. For MT_1_, the contribution of TM5 and TM6 could be excluded by solving the cryo-EM structure of the MT_2_-MT_1_(TM5-6) chimera, which couples to G_s_, but shows no significant differences in TM5 and TM6 length, position, and rotation compared to MT_2_, which does not couple to G_s_. The study of Sadler et al.^30^ suggested a gating function of ICL3 controlling G protein access to receptors. Our data reveals distinct roles of ICL3 for G protein coupling to MT_1_, while ICL3 plays a critical supportive role in stabilizing G_s_ coupling, it is dispensable for G_i_ coupling to MT_1_.

Coupling of MT_1_ to G_i_ occurs upon acute melatonin stimulation. Coupling to G_s_ is also of physiological relevance as it occurs upon long-term melatonin stimulation, mimicking the conditions at dawn, when receptors are exposed to melatonin throughout the night. Stimulation of the G_s_/cAMP/PKA pathway was observed in our study ex vivo and in vivo in both melatonin-proficient and -deficient mouse PT expressing endogenous MT_1_ regardless of ambient photoperiod, which seems to be independent of internal circadian timing. Previously, melatonin has been shown to have a dual impact on the rhythmic transcription of PT cells^31^. At the beginning of the night, melatonin acutely suppresses cAMP levels, which is consistent with the well-documented G_i_-coupling of the MT_1_ receptor. At dawn, melatonin has a delayed heterologous sensitizing effect on the adenylyl cyclase system^32^, which was shown by the sensitization of the signaling of adenosine A_2b_ receptor, the most prominently expressed G_s_-coupled receptor in the PT^33^. Our study indicates that apart from this heterologous sensitization by amplifying the A_2b_ receptor response, the G_i_–G_s_ switch of MT_1_ coupling is likely to contribute to the increased activity of the cAMP/PKA pathway at the end of the night, thus enhancing the contrast of cAMP-dependent rhythmic transcription in PT cells at the night/day transition.

Several limitations of this study should be acknowledged. First, in vivo signaling was assessed at a single melatonin dose and at a single time point, leaving open questions regarding the minimal effective melatonin dose and potential time-of-day–dependent dynamics of MT_1_ G_i_–G_s_ switching. Second, our analyses focused primarily on the PT, and broader hypothalamic network effects, including those involving SCN, remain to be examined. Finally, behavioral or physiological alterations affected by the observed signaling shift should be determined in future studies.

These results provide fundamental insights on the molecular mechanisms underlying the synchronization effects of melatonin, where the acute G_i/o_ pathway upon melatonin rise would signal the beginning of the dark phase of the light/dark cycle, and the switch to G_s_-coupling would signal the night/day transition.

## Methods

### Ethics statement

All animal experiments were approved by the Animal Care and Use Committees of Aichi Medical University (protocol number 2022-62) and Kyushu University (protocol number A25-228-1). All animal experimental procedures were conducted in accordance with the institutional guidelines for the use of experimental animals.

### Material

HEK293T, Sf9, and HEK293F cells were purchased from ATCC, Gibco, and Thermo Fisher Scientific, respectively. The expression constructs of melatonin receptors used in this study were as follows. For Fig. 1a, b, C-terminal untagged Flag-MT_1_ and HA-MT_2_ constructs were used as described previously^22^. For other experiments, C-terminal-tagged constructs (Flag-MT_1_-NP and Flag-MT_2_-NP) were generated by PCR-based modification using plasmids obtained from Addgene (Plasmid #66443^34^ and #66444^34^). Melatonin receptors (MTR) mutants were generated by PCR using PNK-phosphorylated primers carrying the intended mutation, followed by self-ligation with T4 DNA ligase (Nippon Gene), as described previously^35^. Chimeric MTRs were generated by PCR using the In-Fusion HD cloning kit (Clontech) as described previously^36^. cDNA encoding LgBit–miniG_s_ protein or –miniG_s/i_ chimera protein was provided by Dr. Nevin Lambert^37^ (Augusta, Georgia, USA). GPR62-Rluc was presented previously^22^.

### Cell culture and transfection

HEK293T cells were transiently transfected with Jet-PEI or PEI-Max according to the manufacturer’s protocol^35,36^. Briefly, approximately 0.3 × 10⁶ cells per well were seeded into 12-well plates. After 6 h, a total of 1 µg DNA (expression plasmid supplemented with empty vector) was mixed with 3 µL of transfection reagent, incubated for 15 min, and then added to the cells. On the following day, cells were transferred to white 96-well plates, and experiments were performed 48 h post-transfection.

### miniG protein nanoluc complementation assay

Nanobit complementation assay was performed as following: HEK293T cells seeded in a 12-well plate were co-transfected with LgBiT-fused miniG_s_　(5 ng/well) or miniG_s/i_ (15 ng/well)^37^ together with 5 ng/well NP-fragment–tagged^21^ human Flag-MT_1_ or Flag-MT_2_ receptors or their chimera proteins. Cells were reseeded into white 96-well plates on the following day. After 46 h, the culture medium was replaced with phenol red-free DMEM. Two hours later, 5 µM coelenterazine H was added, and baseline luminescence was recorded. Cells were then stimulated with vehicle or 1 µM melatonin, and luciferase activity was measured. Data represent luminescence values obtained 2 min after melatonin stimulation.

### miniG protein recruitment assay by BRET

Bioluminescence resonance energy transfer (BRET) experiments were performed in HEK293T cells transiently co-transfected in 12-well plates with Rluc-C-terminal tagged human MT_2_ (100 ng/well) or human GPR62 (10 ng/well) or MT_1_-Rluc8 (10 ng/well) receptors together with 30 ng/well of Venus-fused miniG_s_ or miniG_s/i_ (obtained from Dr. Nevin Lambert^37^). Twenty-four hours later, cells were reseeded into white 96-well plates. The day after, the cell culture medium was replaced with HBSS, and coelenterazine h was added to yield a final concentration of 5 µM. Cells were then stimulated with vehicle (for GPR62) or concentration–response of melatonin (for MT_2_ and MT_1_). BRET readings were collected using a Mithras LB940 instrument that allows the sequential integration of the signals detected in the 465–505 and 515–555 nm windows using filters with the appropriate band pass and by using MicroWin 2000 software.

### In vitro cAMP assay by HTRF

cAMP accumulation was assessed by HTRF (Fig. 1a, b) under suspension conditions as described previously^22^. HEK293T cells were transfected with varying amounts of Flag-MT_1_ without any C-terminal tag (1 µg, “high;” or 10 ng plus 990 ng empty vector, “low”) or 1 µg HA-MT_2_ without any C-terminal tag plasmids in 12-well plates. After 48 h, cells were dispensed into 384-well plates (4000 cells per well) and stimulated with 2 µM forskolin in the presence of the indicated melatonin concentrations for 30 min at room temperature in PBS supplemented with 1 mM 3-isobutyl-1-methylxanthine (IBMX; Sigma-Aldrich, France). Cells were then lysed, and cAMP levels were quantified following the manufacturer’s instructions (Cisbio Bioassays, France). Where indicated, cells were pretreated with PTX (400 ng/mL, 4 h). Luminescence was recorded using an Infinite F500 microplate reader (Tecan).

### cAMP monitoring by CAMYEL-BRET sensor

cAMP measurement by CAMYEL-BRET sensor was performed as previously described^38^. HEK293T cells were co-transfected with 500 ng CAMYEL sensor and 500 ng melatonin receptor (Flag-MT_1_-NP or Flag-MT_2_-NP and their mutants or chimera receptor) plasmids in 12-well plates. The following day, cells were reseeded into 96-well plates. After 46 h, the culture medium was replaced with phenol red-free DMEM. Two hours later, 5 µM coelenterazine H was added, and baseline BRET signals were recorded. Cells were then treated with vehicle or 5 µM forskolin in the presence of the indicated melatonin concentrations (without IBMX), and real-time BRET changes were monitored. Data shown correspond to measurements taken 10 min after melatonin stimulation. BRET was measured by TECAN SPARK.

### Animals

Since PT expressed not only MT_1_ but also G_s_-coupled thyroid-stimulating hormone receptor related with melatonin signaling^39,40^, we compared melatonin-deficient C57BL/6J and melatonin-proficient CBA/N mice. Only male mice were used to avoid variability associated with the estrous cycle. Five-week-old male C57BL/6JJmsSlc mice and CBA/NSlc (Japan SLC Inc., Shizuoka, Japan) were purchased and housed in plastic cages (170 W × 240 D × 125 H mm, Clea, Tokyo, Japan) maintained at a constant temperature and humidity (23 ± 1 °C; relative humidity of 50–70%). Standard rodent diet (MF; Oriental Yeast Co., Ltd., Tokyo, Japan) and autoclave water were provided ad libitum. C57BL/6J and CBA/N mice (*n* = 6 per each group) were kept under short day conditions (8-h light (200 lx of fluorescent light)/16-h dark cycles (8L16D, 1200 light ON, 2000 light OFF)). Under 8L16D, melatonin-proficient mice showed prolonged melatonin secretion during dark compared with long day conditions^41^. To address the effects of photoperiod, C57BL/6JJmsSlc mice were kept under a 12-h light / 12-h dark cycle (12L12D, 0800 light ON, 2000 light OFF) for more than 3 weeks. Sample sizes (Veh; *n* = 6, MLT; *n* = 7) for in vivo experiments were selected based on prior experience with similar studies and pilot data. The observed effect size was large (*η*² = 0.51), and group sizes of *n* = 6–7 provide approximately 80% power at a two-sided *α* = 0.05 (Supplementary Fig. 3). All animal experiments were approved by the Committee of Animal Care and Use of the Aichi Medical University (protocol number 2022-62) and the Kyushu University (protocol number A25-228-1). All experimental procedures were conducted in accordance with the institutional guidelines for the use of experimental animals.

### Ex and in vivo melatonin treatment

For analysis of melatonin effect on cultured PT/MBH, brain of 8–12-week-old male C57BL/6J mice (*n* = 6 per each group) were quickly removed under general anesthesia at 6 h after dawn in 12L12D and at 2 h after dawn in short day conditions (8L16D), and then immersed in ice-cold Hank’s buffered saline (HBS; #09735-75, Nacalai tesque, Kyoto, Japan). The hypothalamus, containing the PT and MBH, was sectioned at 1 mm thickness by brain matrix. Then, the PT/MBHs were placed on a culture membrane (Millicell-CM PICM0RG50, Merck Millipore, Darmstadt, Germany) in a 35-mm petri dish and cultured at 37 °C with 1.2 mL of medium containing Dulbecco’s modified Eagle medium (DMEM) (Gibco, Carlsbad, CA), 100 U/mL penicillin, and 100 μg/mL streptomycin. After 6 h preincubation, slices were then added with melatonin (finally 10 μM, 0.1% DMSO; Wako, Tokyo, Japan) in C57BL/6J mice, and in CBA/N mice (finally 10 μM) at light offset. After 16 h, proteins of the PT/MBH were extracted for cAMP measurement and western blotting.

For the pharmacological inhibitory experiment, the brains of 8-week-old mice (*n* = 6 per each group) were quickly removed under general anesthesia 2 h after dawn in 8L16D. After slice and 5 h preincubation, slices were then added with MT_1/2_ antagonist luzindole (Luz, 30 µM) for 1 h, and then with melatonin (finally 10 μM) for 16 h.

For in vivo analysis of melatonin effect, mice (Veh; *n* = 6, melatonin; *n* = 7) were kept under 8-h light/16-h dark cycles (8L16D) for 3 weeks, and melatonin (0.26 mM/0.1 mL) was intraperitoneally (i.p.) injected as previously reported^42^, once at ZT8. After 16 h (ZT0), mice were perfused and then fixed on slides with 4% paraformaldehyde (PFA) in 0.1 M phosphate buffer (pH 7.4), and brains were removed and put into the 4% PFA for 48 h, and then transferred to 20 % sucrose/PBS for frozen sectioning of the brain.

### Ex vivo cAMP measurement at PT

Protein extraction of cultured PT/MBH was performed using cell lysis buffer (#9803, Cell Signaling Technology, Tokyo, Japan) containing PDE inhibitor IBMX (3-isobutyl-1-methylxanthine) (0.1 mM; #19624-86, Nacalai Tesque), protease inhibitor cocktail (#P8340, Sigma), and phosphatase inhibitor cocktail 1 (#P2850, Sigma) according to the manufacturer’s instructions.

cAMP measurements were performed with a homogeneous TR-FRET immunoassay using the LANCE cAMP Detection Kit (#AD0262, PerkinElmer, USA), according to the manufacturer’s instructions (PerkinElmer). Cultured PT/MBH tissues homogenates (0.2 μg/μL) were diluted 10 times with stimulation buffer, and added to 10 µL of Alexa Fluor 647 anti-cAMP antibody diluted with stimulation buffer. After incubation for 45 min at room temperature, the reaction was stopped by the addition of 20 µL working solution (10 µL Eu-cAMP and 10 µL ULight-anti-cAMP), and incubated for 1 h at room temperature. The TR-FRET signal was read using a microplate reader, SpectraMax M5 (Molecular Devices) and Nivo (PerkinElmer). cAMP concentrations were determined using GraphPad Prism 10 software (GraphPad Software Inc., San Diego, CA).

### Western blot analysis

Western blot analysis was performed as follows: homogenized PT/MBH was loaded as 2 μg protein samples per lane onto 8 % SDS-polyacrylamide gels. Following separation at 100 mA, membranes were incubated with the following primary antibodies: rabbit monoclonal antibodies against phospho-CREB (Ser133) (1:1000; #9198, Cell Signaling Technology), and CREB (1:1000; #9192, Cell Signaling Technology). Membranes were washed and then incubated with HRP-conjugated goat polyclonal antibody against rabbit IgG (1:10,000; #7074, Cell Signaling Technology). By using ImmunoStar LD (#292-69903, Fujifilm), chemiluminescent images were detected using an Amersham Imager 600 (Cytiva Lifescience) and FUSION SOLO.7S.EDGE (Vilber-Lourmat).

### Immunohistochemistry

Immunohistochemistry was performed by using the Vectastain Elite ABC rabbit IgG kit (Vector, Burlingame, CA), as our previous reports^43^. Frozen sections of paraformaldehyde-fixed, trimmed mice brain (14 µm), including the PT/MBH, were used. Briefly, after drying, the sections were immersed in HistoVT One (pH 7.0, #06380, Nacalai) and heated in a microwave oven for 15 min at low voltage. After blocking with normal rabbit serum (Vectastain), sections were incubated with rabbit polyclonal antibody against phospho-CREB (Ser133) (1:500; #9198, Cell Signaling Technology) at 4 °C for 48 h. After encapsulation with mounting medium, images were detected using a BZ-X800 (Keyence). ImageJ software (NIH) was used to analyze pCREB-immunoreactive (ir) cell numbers as previous report^44^. Data are shown as percent of maximum values (pCREB-ir cells/mm^2^).

### Expression and purification of Gβ_1_-Gγ_2_ dimer

In the previous study, we expressed and purified the G_i1_ heterotrimer, including human Gα_i1_, bovine Gβ_1_, and mouse Gγ_2_ by Ni-NTA affinity chromatography and anion exchange chromatography. As a byproduct of the anion exchange chromatography step, the fraction of Gβ_1_-Gγ_2_ dimer was eluted before the fraction of the G_i__1_ heterotrimer.

### Expression and purification of Nb35

The plasmid encoding Nb35 was prepared as previously reported^27^. The protein was expressed in the periplasm of *E. coli* C41 (Rosetta) cells cultured in LB medium supplemented with 1 mM IPTG for 20 h at 25 °C. After 20 h, the cells were collected and disrupted by ultrasonication in hypotonic buffer (20 mM Tris-HCl, pH 7.5, 150 mM NaCl and 2 mM MgCl_2_), and the Nb35 protein was purified by Ni-NTA affinity chromatography and then subjected to size-exclusion chromatography on a HiLoad Superdex75 16/600 column. Peak fractions were pooled and concentrated to 3 mg ml^–1^.

### Expression and purification of MT_1_-miniG_s_-Gβ_1_-Gγ_2_ complex

The HA signal sequence, the FLAG epitope tag (DYKDDDDK), the ALFA tag (SRLEEELRRRLTE)^45^ and the GSGSG linker were fused to the N-terminus of MT_1_, in this order. In addition, we fused the miniG_s_ fragment to the C-terminus of MT_1_.

In the MT_2_-MT_1_ chimera construction, we substituted TM5-ICL3-TM6 of MT_2_ to the MT_1_ sequence (from Met200^5.51^ to Phe256^6.53^) between Pro212^5.50^ and Ile270^6.54^. And we fused the same fragments as MT_1_–G_s_ to the N-terminus of MT_1_. In contrast, we fused the mini-G_s_^star^ protein created in our previous study^27^ to the C-terminus of MT_1_. All the above constructions were cloned into the pEGBacmam vector.

Then we infected a one-tenth volume of a solution containing the virus encoding the above construction to the HEK293F cells at a density of 3–4 × 10^6^ cells ml^−1^, and grown at 37 °C for 20 h in FreeStyle 293 Expression Medium (Gibco). After sodium butyrate (FujiFilm Wako Pure Chemical Corporation) was added to 10 mM, the cells were incubated further at 30 °C for 48 h.

The HEK293F cells were collected by centrifugation at 5000 × *g* for 10 min, lysed in buffer containing 50 mM Tris (pH 8.0), 150 mM NaCl, and 10% glycerol, and fresh frozen by liquid nitrogen. The cells were thawed and directly solubilized at 4 °C for 2 h in the solubilization buffer, containing 50 mM Tris (pH 8.0), 150 mM NaCl, 10% glycerol,1.5% DDM, 0.15% CHS, 5.2 μg ml^−1^ aprotinin, 2.0 μg ml^−1^ leupeptin, 1.4 μg ml^−1^ pepstatin A, 100 μM PMSF, and 10 μM melatonin (Wako). The soluble fraction containing MT_1_-miniG_s_ was separated by ultracentrifugation (186,000 × *g* for 30 min), and the supernatant was mixed with the purified Gβ_1_-Gγ_2_ dimer (0.8 mg l^−1^ HEK cells), the purified Nb35 (0.5 mg l^−1^ HEK cells), and a final 50 mU/mL of Apyrase (New England Biolabs). Then they were stirred at 4 °C overnight. The solution was supplemented with 2 mM CaCl_2_ at the final concentration, mixed with the M1 anti-FLAG resin, and rotated at 4 °C for 2 h.

The resin was collected by centrifugation (500 × *g* for 3 min) and washed with 20 column volumes of buffer, containing 50 mM Tris-HCl (pH 8.0), 150 mM NaCl, 10% glycerol, 0.03% LMNG, 0.003% CHS, 2 mM CaCl_2_, and 1 μM melatonin. The complex was eluted with buffer, containing 50 mM Tris-HCl (pH 8.0), 150 mM NaCl, 10% glycerol, 0.03% LMNG, 0.003% CHS, 2 mM CaCl_2_, 1 μM melatonin, 5 mM EDTA (pH 8.0) and 125 μg ml^−1^ FLAG peptide (Synthesized by GenScript), concentrated and purified by size-exclusion chromatography on a Superdex200 10/300 Increase (GE) column, using buffer containing 50 mM HEPES-NaOH (pH 7.5), 150 mM NaCl, 100 μM TCEP, 0.01% LMNG, 0.001% CHS and 1 μM melatonin. Peak fractions were pooled and concentrated to around 7 mg ml^−1^ with a centrifugal filter device (Millipore 50 kDa molecular weight cutoff).

### Grid preparation and cryo-EM data collection

The purified complex solution was applied to freshly glow-discharged Au 300 mesh R1.2/1.3 grids (Quantifoil), using a Vitrobot Mark IV (FEI) at 4 °C, with a blotting time of 4 s under 100% humidity conditions. The grids were then plunge-frozen in liquid ethane cooled to the temperature of liquid nitrogen.

Cryo-EM data were collected using a Titan Krios G3i microscope (Thermo Fisher Scientific), running at 300 kV and equipped with a Gatan Quantum-LS Energy Filter (GIF) and a Gatan K3 Summit direct electron detector in the electron counting mode (The University of Tokyo, Japan). Movies were recorded at a nominal magnification of ×105,000, corresponding to a calibrated pixel size of 0.83 Å, with a total dose of approximately 50 electrons per Å^2^ per 48 frames. The data were automatically acquired using the EPU software (Thermo Fisher Scientific), with a defocus range of −0.8 to −1.6 μm. For MT_1_-miniG_s_, 10,373 movies were obtained. For MT_1_–MT_2_-miniG_s_, 8174 movies were obtained.

### Image processing

All acquired movies were dose-fractionated and subjected to beam-induced motion correction implemented in RELION 3.1^46^. The contrast transfer function (CTF) parameters were estimated using patch CTF estimation in cryoSPARC v3.3^47^.

For MT_1_-miniG_s_, particles were initially picked from a small fraction with the Blob picker and subjected to several rounds of two-dimensional (2D) classification in cryoSPARC using 897 movies. Selected particles were used for template picking on the full dataset, and 8,196,012 particles were picked and extracted with a pixel size of 3.32 Å, followed by 2D classification, ab initio reconstruction, homogeneous refinement, and heterogeneous refinement, displayed in the Supplementary Fig. 6. A total of 624,555 particles were used for training of topaz model^48^, and 3,524,939 particles were extracted. After 2D classification, heterogenous refinement and nonuniform (NU) refinement, 449,204 particles were transferred into RELION 3.1 and were processed by 3D classification without alignment using TMD mask with several T parameters. After 3D refinement with SIDESPLITTER^49^, particle polishing, re-extract as 1.0 Å/pix, and removal of the duplicated particles, 85,855 particles were transferred into cryoSPARC.

Finally, these 85,855 particles were reconstructed using NU refinement, resulting in a 3.03 Å overall resolution reconstruction, with the gold standard Fourier shell correlation (FSC = 0.143) criteria in cryoSPARC. Moreover, the 3D model was refined with a mask on the receptor by local refinement. As a result, the local resolution of the receptor portion was estimated as 3.24 Å by cryoSPARC. In parallel, the 3D model was refined with a mask on the G protein by local refinement, resulting in the local resolution at 2.9 Å estimated by cryoSPARC.

For MT_2_-MT_1_-miniG_s_, particles were initially picked from a small fraction with the Blob picker and subjected to several rounds of 2D classification in cryoSPARC using 2429 movies. Selected particles were used for template picking on the full dataset, and 54,229,298 particles were picked and extracted with a pixel size of 3.32 Å, followed by 2D classification, ab initio reconstruction, homogeneous refinement, and heterogeneous refinement, displayed in the Supplementary Fig. 10. A total of 272,585 particles were selected as the best class and were transferred into RELION 4.0. After 3D classification without alignment using TMD mask with several T parameters, 74,839 particles were selected, processed by 3D refinement with SIDESPLITTER^49^, particle polishing, and re-extract as 1.0375 Å/pix and transferred into cryoSPARC.

Finally, these 74,839 particles were reconstructed using NU refinement, resulting in a 2.94 Å overall resolution reconstruction, with the gold standard Fourier shell correlation (FSC = 0.143) criteria in cryoSPARC. Moreover, the 3D model was refined with a mask on the receptor by local refinement. As a result, the local resolution of the receptor portion was estimated as 3.43 Å by cryoSPARC. In parallel, the 3D model was refined with a mask on the G protein by local refinement, resulting in the local resolution at 2.82 Å estimated by cryoSPARC.

### Model building and validation

The cryo-EM structures of the MT_1_–G_i_ complex (PDB 7DB6)^12^, MT_2_–G_i_ complex (PDB 7VH0)^13^, and OR51E2-miniG_s_ (PDB 8F76)^50^ were used as starting templates for modeling the MT_1_, MT_2_-MT_1_, miniG_s_, Gβγ, and Nb35 components. Initially, these models were positioned into the density map using jiggle fit in COOT^51^. Then these models were modified by COOT and refined with phenix.real_space_refine (v1.19)^52,53^ and the secondary structure restraints from phenix.secondary_structure_restraints. Cif format definition (ML1) downloaded from PDB was used for modeling the melatonin molecule at the ligand binding site. Statistics are reported in Supplementary Table 3.

### Computer simulations

MD simulations were conducted on melatonin receptor systems derived from cryo-EM structures. All simulated systems and their stability parameters are listed in (Supplementary Table 1 and Supplementary Figs. 16, 17). MD simulations were performed on five distinct melatonin receptor-G protein complexes: MT_1_ + G_i_ (PDB: 7DB6), MT_1_ + G_s_, MT_2_ + G_i_ (PDB: 7VH0), MT_2_-MT_1_(TM5-ICL3-TM6) + G_s_, and MT_2_-WT + G_s_. The cryo-EM structures were prepared for simulations using Schrödinger Maestro (2025-1)^54^. ICL3 regions were rebuilt by grafting the corresponding segments from GPCRdb active state AlphaFold2-Multistate models^55,56^ (version 15-05-2024): MT_1_ AlphaFold ICL3 (V217–P231) with an average pLDDT confidence score of 65.17, and MT_2_ AlphaFold ICL3 (A230-P244) with an average pLDDT confidence score of 73.62. The grafting boundaries were determined by identifying structured TM5/TM6 regions flanking the missing ICL3 density in the cryo-EM structures. AlphaFold segments were integrated as follows: MT_1_ + G_i_ used the MT1 AlphaFold model [V198–F244], MT_1_ + G_s_ used the MT_1_ AlphaFold model [V198–N236], MT_2_ + G_i_ used the MT_2_ AlphaFold model [L211–L251], and MT_2_-MT_1_(TM5-ICL3-TM6) + G_s_ used the MT_1_ AlphaFold model [V198–F244]. The experimentally determined chimeric structure was used as the template for both MT_2_-MT_1_(TM5-ICL3-TM6) + G_s_ and MT_2_-WT + G_s_ systems, with the MT_2_-WT + G_s_ variant generated by mutating the MT_1_-derived TM5-ICL3-TM6 segment back to the MT_2_ wild-type sequence using Schrödinger Maestro 3D builder utility. In all systems, D55^2.50^ was protonated as per PROPKA3 predictions^57,58^.

CHARMM-GUI^59–67^ server was used to prepare simulation systems. Membrane protein orientations were determined using the positioning of proteins in membranes (PPM) 2.0^59^ web server prior to embedding in a 1-palmitoyl-2-oleoyl-sn-glycero-3-phosphocholine (POPC) bilayer membrane. Membrane dimensions were optimized for each receptor-G protein complex, ranging from 130 Å × 130 Å to 140 Å × 140 Å, to maintain adequate lipid-protein boundaries and prevent periodic artifacts. Each system was solvated with TIP3P water extending 30–35 Å from the membrane surfaces, with ionic strength adjusted to physiological conditions using 0.15 M NaCl. The resulting simulation systems comprised approximately 250,000 atoms, with total system size dependent on the specific receptor-G protein complex dimensions and corresponding membrane requirements.

All MD simulations were performed using AMBER20^68^ under NPT ensemble conditions at 310 K and 1 bar. The FF19SB^69^ force field was employed for protein parameters, while lipid21^70^ and GAFF^71^ force fields described lipid and ligand interactions, respectively. The OPC^72^ water model was used for solvation, with long-range electrostatic interactions calculated via particle mesh Ewald. A 9 Å van der Waals cutoff was applied throughout all simulations. This force field combination was selected based on current best practices for membrane-embedded GPCR simulations.

System preparation involved energy minimization followed by 47.5 ns of equilibration. Energy minimization was performed using 2500 steepest descent steps followed by 2500 conjugate gradient steps. Temperature equilibration employed the Langevin thermostat (*γ* = 1.0 ps⁻¹) with gradual heating to 310 K over two consecutive 2.5 ns NVT phases using 1 fs timesteps. Pressure equilibration utilized the Berendsen barostat (*τ*_P_ = 1.0 ps) through a series of NPT phases: an initial 2.5 ns phase at 1 fs timestep, followed by two consecutive 10 ns phases at 2 fs timestep, and a final 20 ns phase at 2 fs timestep with restraints applied only to protein and ligand atoms. Harmonic restraints were systematically reduced across the equilibration stages. Lipid restraints decreased progressively to 2.5, 2.5, 1.0, 0.5, 0.1, and 0.0 kcal/mol/Å² across the six equilibration phases. Protein and ligand restraints followed a similar pattern: 10.0, 5.0, 2.5, 1.0, 0.5, and 0.1 kcal/mol/Å². Production simulations were conducted for 1000 ns per replica with five independent replicates per system, using 2 fs timesteps and coordinates saved every 1000 ps. Classical MD simulations were considered equilibrated when Cα root-mean-square deviation (RMSD) of the 7TM bundle stabilized within 1.5 Å for at least 100 ns.

Gaussian accelerated MD (GaMD)^73^ simulations were initiated from the equilibrated system using the dual-boost GaMD method (igamd = 3). The protocol implemented semi-isotropic pressure control for the membrane system (ntb = 2, barostat = 1, ntp = 3, csurften = 3, gamma_ten = 0.0, ninterface = 2) with a target pressure of 1.0 bar (pres0 = 1.0 bar, taup = 1.0 ps). Bonds involving hydrogen were constrained with SHAKE (ntc = 2, ntf = 2), allowing a 2 fs timestep.

The simulations followed a standard two-stage workflow. First, a 10 ns statistics-collection stage (iE = 1) was performed from a fresh start (irest = 0, ntx = 1, irest_gamd = 0). This involved classical MD, statistics collection, bias preparation, and biased MD, controlled by ntcmdprep, ntcmd, ntebprep, nteb, and ntave. The production stage was then initiated by restarting from this point (irest = 1, ntx = 5, irest_gamd = 1) in production mode (iE = 2), applying a biasing potential based on the collected statistics. During production, the boost parameters were fixed (sigma0P = 6.0, sigma0D = 6.0) and no further statistics were collected (ntcmd = 0, nteb = 0). Convergence of GaMD simulations was assessed by monitoring boost potential distributions (Supplementary Fig. 18).

Structural dynamics analyses were performed on simulation trajectories using AmberTools23 CPPTRAJ^74^ for trajectory processing, imaging, and alignment. The simulation trajectories were aligned to the post-minimization starting structure using Cα atoms of residues (MT_1_ systems: S28–R54, F65–V84, S103–Y128, L145–N162, I189–Q216, F234–G258, and V278–Y295; and MT_2_ systems: A42–V65, F78–I101, A115–Y139, P158–P174, Y200–A230, F257–L272, and F290–I306) of the 7-transmembrane bundle as the reference framework. RMSD and root-mean-square fluctuation calculations were conducted relative to these aligned post-minimization structures to assess structural stability and regional flexibility throughout the simulation period.

For interaction energy analysis, noncovalent interaction energies between individual receptor residues and the G protein α5 helix were calculated using the NAMDenergy utility^75,76^ (v1.6) distributed with VMD 1.9.3^77^. The energy calculations employed a 9 Å cutoff distance with a 7.5 Å van der Waals smoothing switch distance. Energy analyses were performed on every 10th frame of the trajectories (100 frames total from each 1000-frame simulation), corresponding to a 10 ns sampling frequency, across independent replicas of 1 μs MD simulations. Results are reported as mean ± standard deviation values calculated across all replicas to ensure statistical robustness of the findings.

MM/GBSA binding free energies between the GPCR and full G protein were computed using MMPBSA.py^78^ in Amber 20^68^. Calculations were averaged over five (three for GaMD) replicas for each complex, using 100 equally spaced snapshots (10 ns intervals) extracted from each 1 μs MD trajectory. Results are reported as the mean effective binding free energy Δ*G* (kcal/mol) and standard deviation (SD). Trajectories were stripped of all explicit solvent and associated molecules, and subsequent energy evaluations were performed without periodic boundary conditions using an effectively infinite nonbonded cutoff. Polar solvation energies were calculated by the generalized Born (GB) model (igb = 5)^79^ with 0.150 M salt concentration, while nonpolar solvation contributions were estimated from the solvent-accessible surface area (SASA). A completed MD simulations checklist is provided as Supplementary Table 4.

### Statistical analysis

All data are presented as the mean ± standard error of the mean (SEM). Normality was assessed by Shapiro–Wilk (Supplementary Fig. 3; all *p* > 0.05). For paired analyses (ratio-paired t-tests and paired multiple t-tests), equality of variances was not assumed. For repeated-measures one-way ANOVA, sphericity was not assumed, and the Geisser–Greenhouse correction was applied. For the unpaired t-test in ex vivo and in vivo experiments, Welch’s correction was applied; therefore, equality of variances was not assumed. Statistical significance was assessed using ratio-paired t-tests, and exact two-tailed *p*-values are reported for Figs. 1c–f, 4d, and 5f, g. For Fig. 2h, statistical significance was assessed using multiple two-tailed t-tests with Holm–Šidák correction for multiple comparisons, and exact adjusted *p*-values are reported. For Fig. 1l, statistical analysis was performed using ordinary one-way ANOVA followed by Dunnett’s multiple comparisons test, and exact adjusted *p*-values are reported. For Figs. 5b–e and 6c, d, statistical analysis was performed using repeated-measures one-way ANOVA with Geisser–Greenhouse correction followed by Bonferroni’s multiple-comparisons test, and exact adjusted *p*-values are reported. For the unpaired t-test in ex vivo and in vivo experiments, Welch’s correction was applied, and two-tailed *p*-values are reported (Fig. 1h, j, o). *P* < 0.05 were considered statistically significant. All analyses were performed using GraphPad Prism versions 6, 8, 10, and 11 (GraphPad Software Inc., San Diego, CA).

### Reporting summary

Further information on research design is available in the Nature Portfolio Reporting Summary linked to this article.

## Supplementary information

Supplementary Information

Reporting Summary

Transparent Peer Review File

## Source data

Source Data

## Data Availability

Source data are provided with this paper. The atomic coordinates generated in this study have been deposited in the Protein Data Bank under accession codes 11OD (MT_1_-miniG_s_ complex) and 11OZ (MT_2_-MT_1_(TM5-ICL3-TM6)-miniG_s_ complex). The cryo-EM maps generated in this study have been deposited in the Electron Microscopy Data Bank under accession codes EMD-75880 (MT_1_-miniG_s_ overall map), EMD-76180 (MT_1_-miniG_s_ TMD map), EMD-76181 (MT_1_-miniG_s_ G protein map), EMD-75909 (MT_2_-MT_1_ (TM5-ICL3-TM6)-miniG_s_ overall map), EMD-76182 (MT_2_-MT_1_(TM5-ICL3-TM6)-miniG_s_ TMD map), and EMD-76183 (MT_2_-MT_1_ (TM5-ICL3-TM6)-miniG_s_ G protein map). The molecular dynamics simulation data generated in this study have been deposited in Zenodo at Zenodo entry 17107103. Source data are provided with this paper.
