# Supplementary material for: Structural basis and physiological significance of non-canonical Gs coupling to the melatonin MT1 receptor
Source: Nat Commun. 2026 May 21;17:6706. doi: 10.1038/s41467-026-73555-6 (PMC13385403; doi:10.1038/s41467-026-73555-6)
Supplement: Supplementary file 1 — Supplementary Information [file 41467_2026_73555_MOESM1_ESM.pdf]

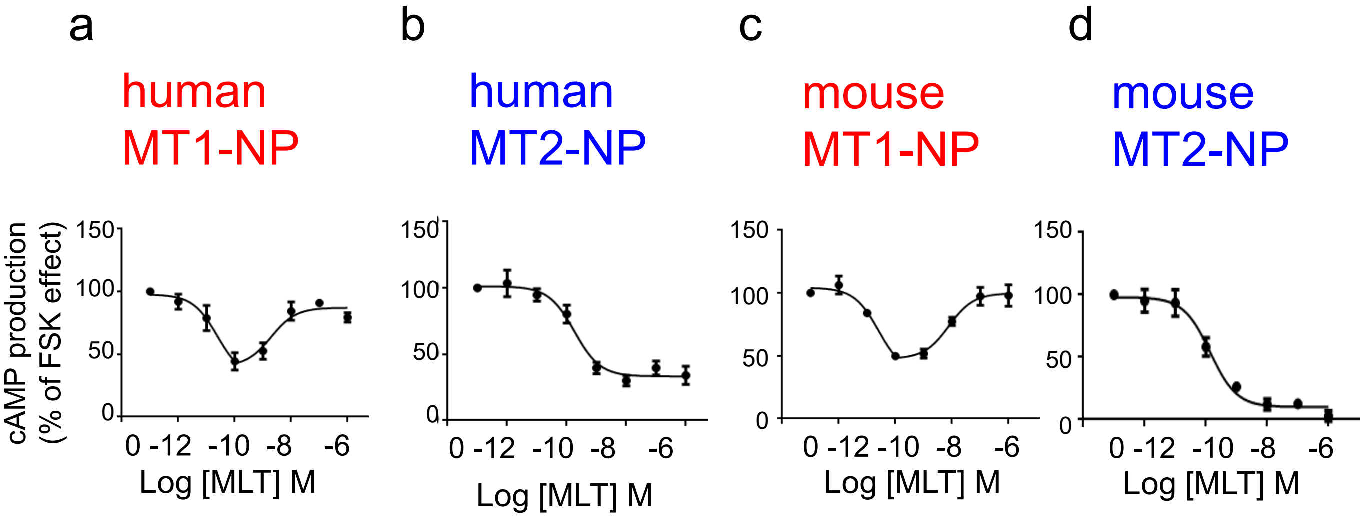

Supplementary Fig.1 Dose–response analysis of melatonin-induced cAMP production via C-tail tagged human or mouse melatonin receptor.

Dose–response analysis of melatonin-induced cAMP production in HEK293T cells expressing Flag-tagged human MT1-NP (a), human MT2-NP (b), mouse MT1-NP (c) and mouse MT2-NP (d) . Cells were treated with forskolin (5 μM) together with the indicated concentrations of melatonin for 10 min. cAMP levels were monitored using the BRET-based CAMYEL biosensor. Data represent means ± SEM from three independent experiments performed in duplicate (n = 3).

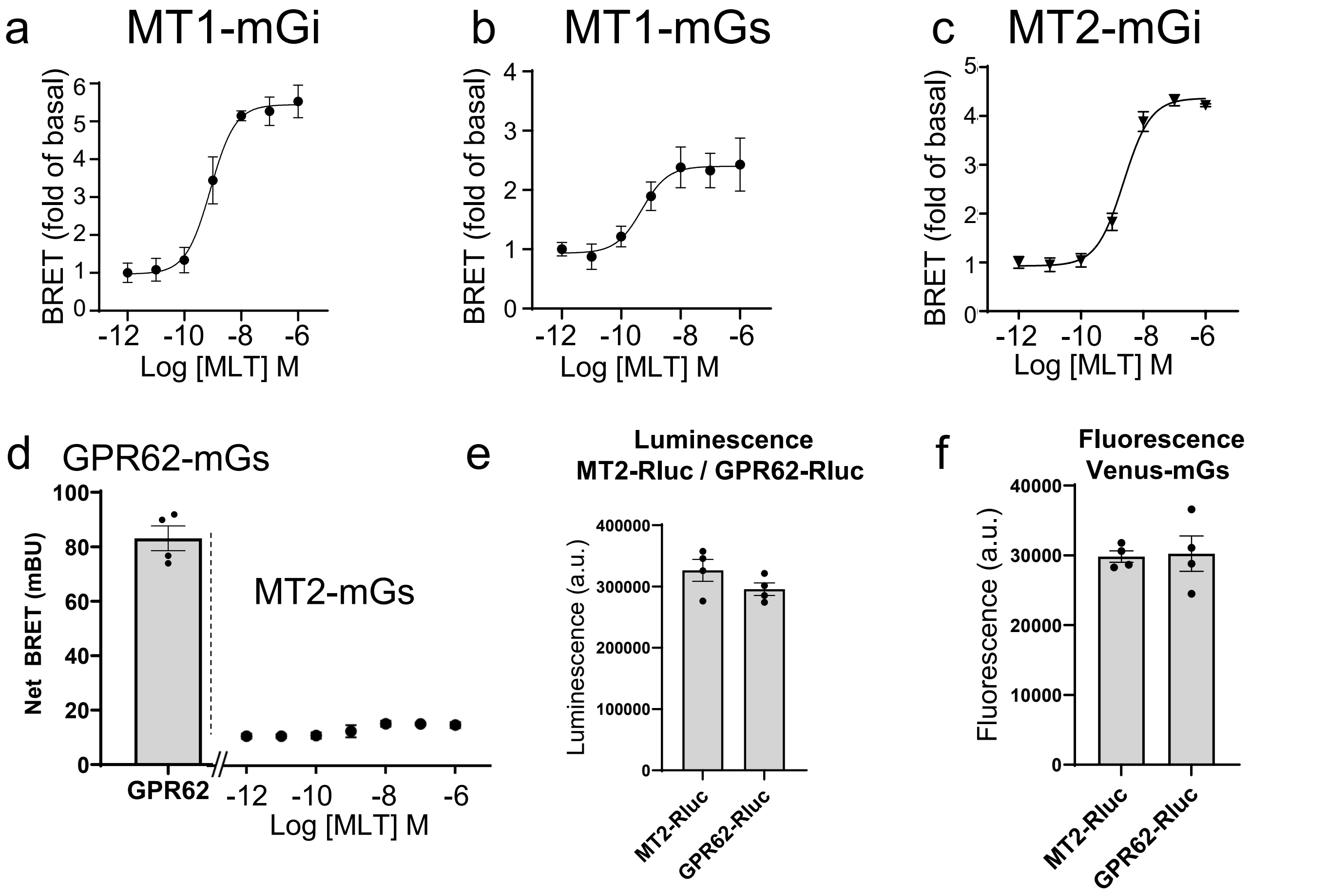

Supplementary Fig. 2 miniG protein BRET assay between melatonin receptors and Venus-miniG proteins. (a-c) BRET assays between MT1-Rluc and Venus–miniGi (pEC50: 9.08 ± 0.786) (a) and Venus–miniGs (pEC50: 9.31 ± 0.365) (b), and MT2-Rluc and Venus–miniGi (pEC50: 8.63 ± 0.08448) (c). (d) Basal (constitutive) recruitment of Venus-miniGs to GPR62-Rluc and melatonin-stimulated recruitment to MT2-Rluc. (e,f) Expression controls of BRET donors (Rluc fusion proteins) (e) and acceptors (Venus-mGs) (f) of panel d. Data are mean ± SEM from four independent biological experiments (n=4).

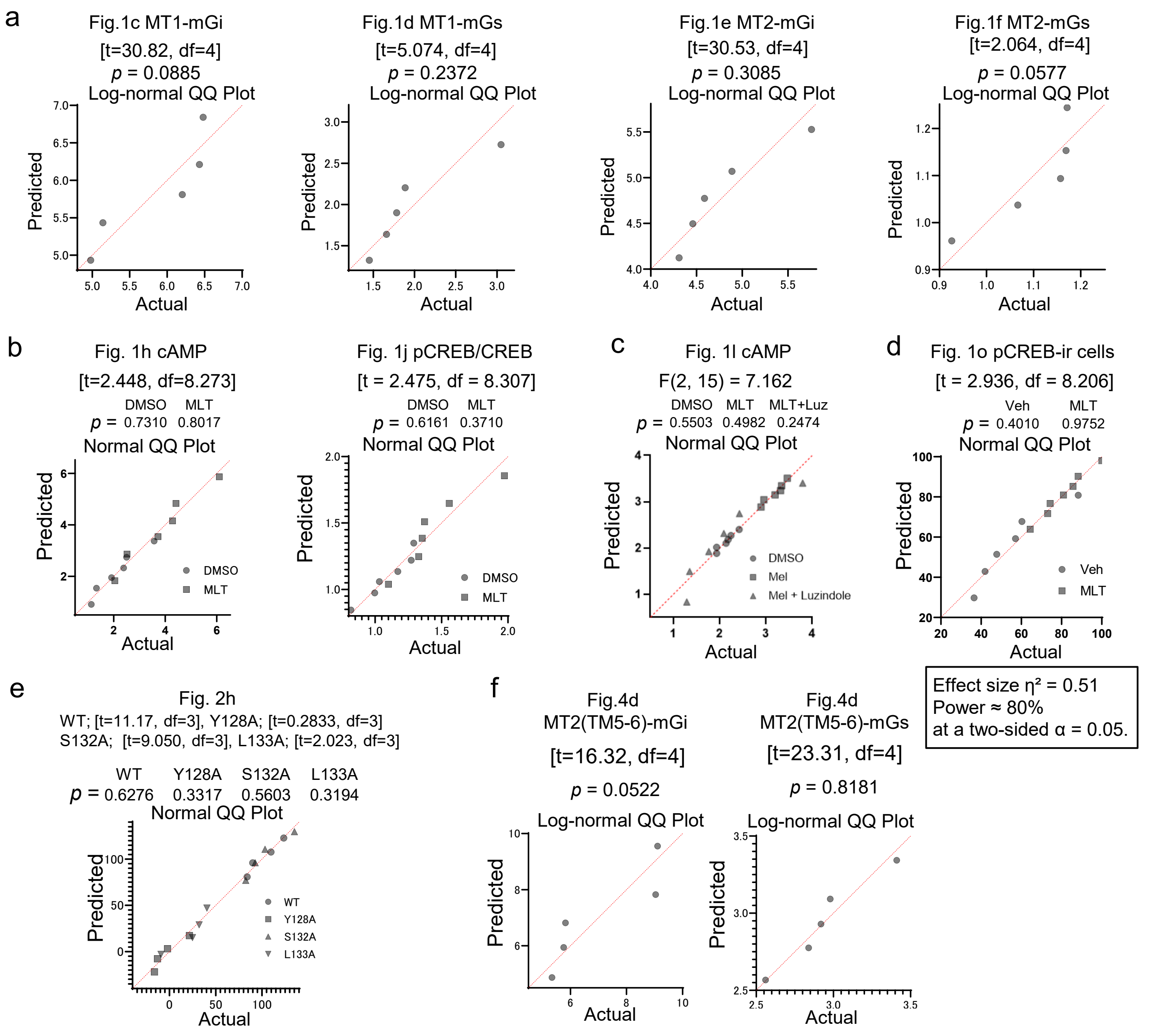

Supplementary Fig. 3 Normal and log-normal Q–Q plots for assessment of distributional assumptions. **(Continued on next page)** (a-i) Normality assumptions were evaluated using the Shapiro–Wilk test, and the corresponding Q–Q plots are shown for each dataset used in the main statistical analyses. Panels are arranged to match the order of the corresponding main-figure datasets. For fold-change (ratio) datasets (baseline/vehicle-normalized) (a, f, h), analyses were performed on the log scale, and log-normal Q–Q plots of the log-transformed values (log ratios) are shown. For datasets normalized to 0 concentration (set to 100%) (g, i), normality was assessed on the within-subject differences (Δ) relevant to the planned comparisons, and normal Q–Q plots of Δ are shown. Shapiro–Wilk p-values are indicated within each panel; all datasets satisfied the normality criteria ($p > 0.05$). Detailed test statistics are indicated in the corresponding panels. For t-tests, t and df denote the t statistic and degrees of freedom, respectively (a, b, d, e, f, h). For ordinary one-way ANOVA, F(DFn, DFd) denotes the F statistic with numerator and denominator degrees of freedom, respectively (c). For repeated-measures one-way ANOVA with Geisser–Greenhouse correction, F(DFn, DFd) denotes the F statistic with corrected numerator and denominator degrees of freedom, respectively (g, i). For Fig. 1o (d), the observed effect size was large ($\eta^2 = 0.51$), and group sizes of n = 6–7 provide approximately 80% power at a two-sided $\alpha = 0.05$.

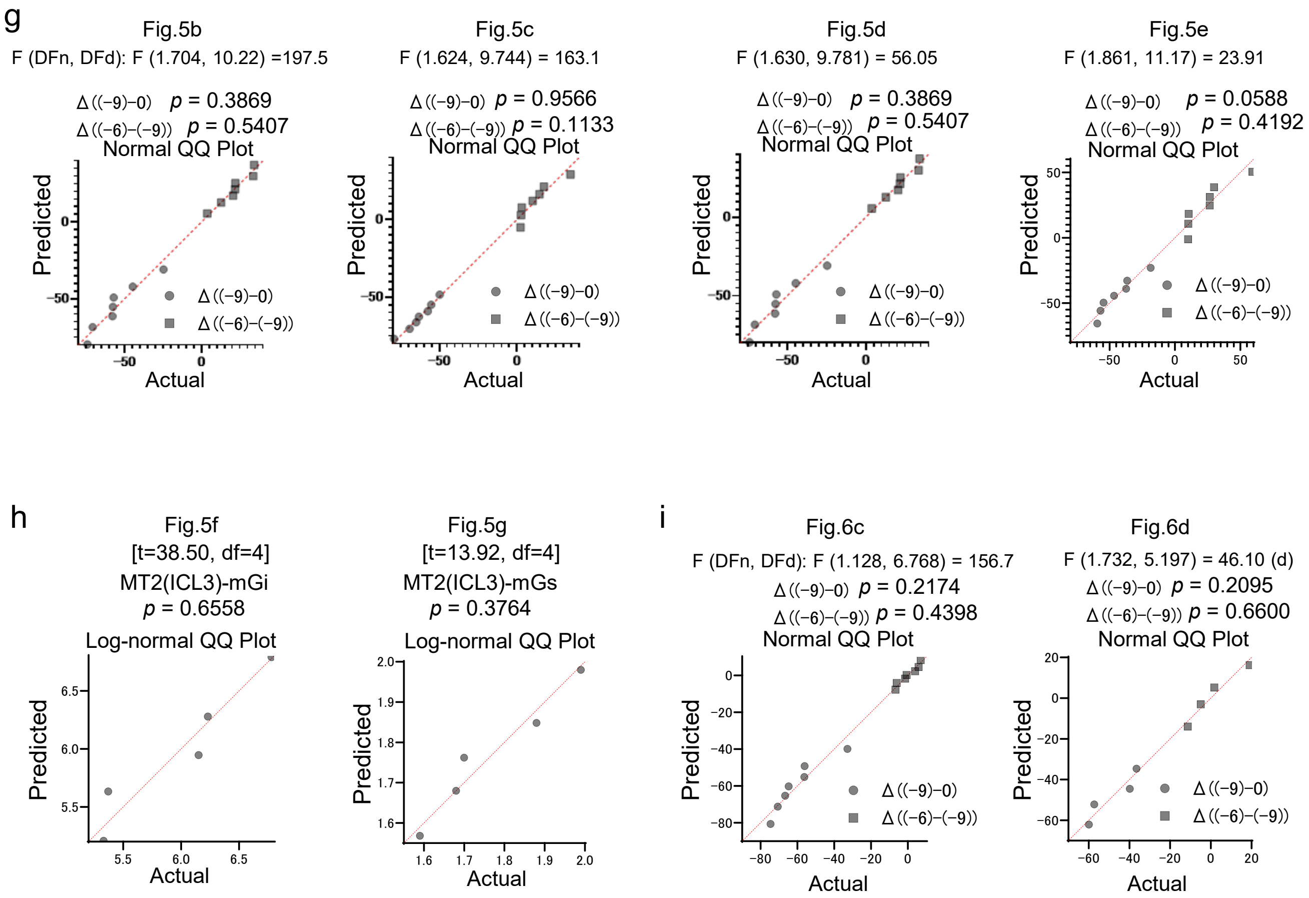

Supplementary Fig. 3 Normal and log-normal Q–Q plots for assessment of distributional assumptions. (continued)

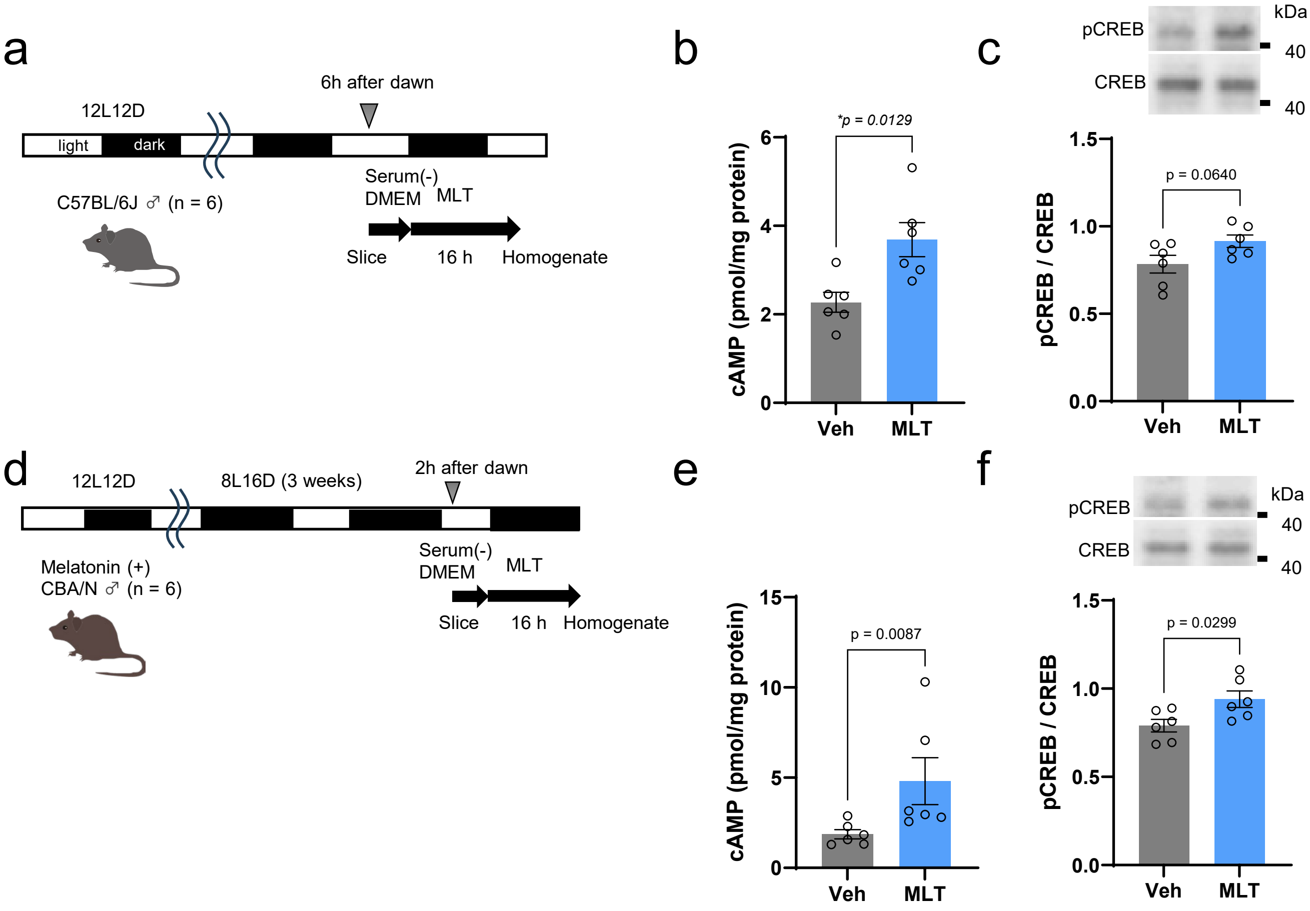

Supplementary Fig. 4 Additional validation of MLT-induced cAMP-pCREB signaling in PT slices. (**a**) Experimental design for *ex vivo* stimulation under 12L12D conditions. Coronal brain slices containing the pars tuberalis (PT) were prepared from C57BL/6J mice (n = 6) and incubated in serum-free DMEM with melatonin (MLT, 10 μM) for 16 h starting 6 h after dawn. (**b**) cAMP levels in PT slices treated with Veh or MLT (**p* = 0.0129, two-tailed unpaired t-test with Welch's correction). Normality was assessed by Shapiro–Wilk ($p > 0.05$). (**c**) Representative immunoblot and densitometric quantification of phosphorylated CREB (pCREB) normalized to total CREB in PT slice homogenates treated with Veh or MLT (*p* = 0.0640, two-tailed unpaired t-test with Welch's correction). Normality was assessed by Shapiro–Wilk ($p > 0.05$). (**d**) Experimental design for *ex vivo* stimulation using melatonin-proficient CBA/N mice (n = 6) entrained to 12L12D followed by 8L16D for 3 weeks. PT slices were prepared 2h after dawn and incubated with MLT (10 μM) for 16 h. Normality was assessed by Shapiro–Wilk ($p > 0.05$). (**e**) cAMP levels in PT slices treated with Veh or MLT (**p* = 0.0087, Mann–Whitney U test.) Normality was rejected by the Shapiro–Wilk test for the MLT group (p = 0.0239); therefore, a nonparametric test was used.(**f**) Representative immunoblot and densitometric quantification of phosphorylated CREB (pCREB) normalized to total CREB in PT slice homogenates treated with Veh or MLT (* *p* = 0.0299, two-tailed unpaired t-test with Welch's correction). Normality was assessed by Shapiro–Wilk ($p > 0.05$). Data are shown as mean ± s.e.m. with scatter plot. Statistical analyses and exact *P* values are indicated in the panels.

Related to Supplementary Figure S4c Western blots used for densitometry quantification

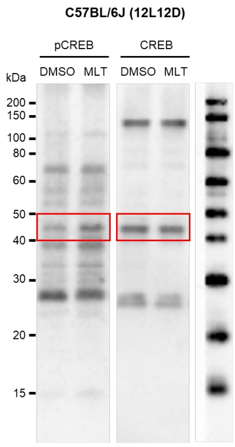

Related to Fig.1i Western blots used for densitometry quantification

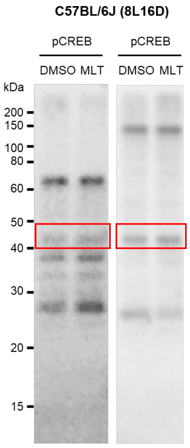

Related to Supplementary Fig 4f Western blots used for densitometry quantification

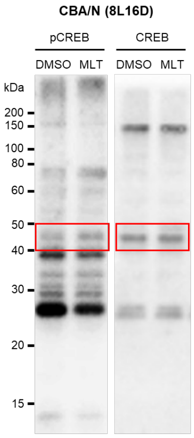

Supplementary Fig. 5 Images for uncropped blots

Uncropped images of western blots from the corresponding cropped western blots, shown in the main text. Molecular weight markers are shown. Figure subpanel is indicated for each blot. CREB, cyclic adenosine monophosphate response element binding protein

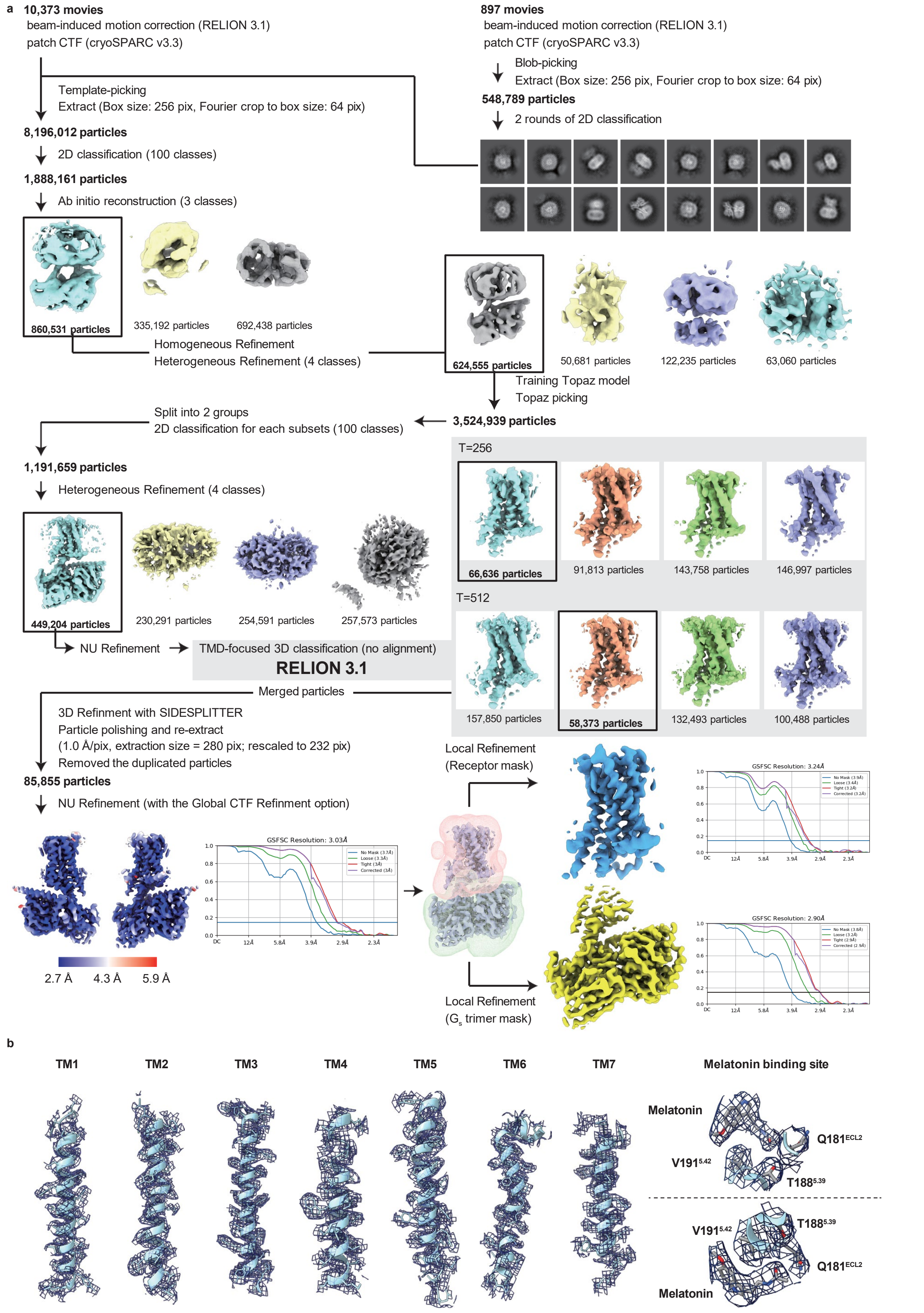

Supplementary Fig. 6 Cryo-EM analysis of the MT_1-$miniG_s$ complex

(a) The workflow of Cryo-EM analysis of MT_1-$miniG_s$ complex using RELION 3.1 and cryoSPARC v3.3.
(b) The structure of MT_1-$miniG_s$ complex in cryo-EM map at TMD and melatonin binding site of MT_1.

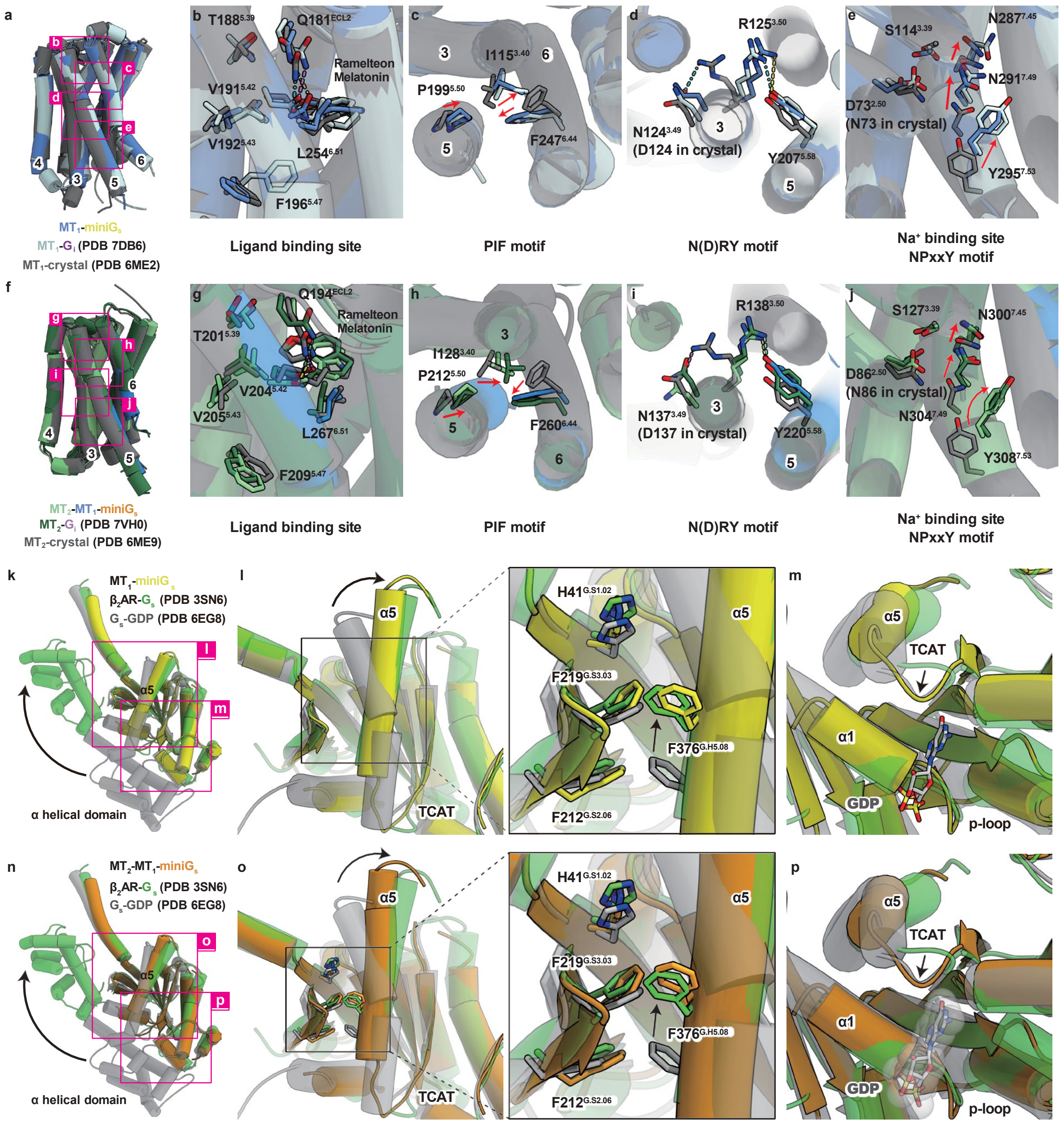

Supplementary Fig. 7 Structural comparison of the MT_1-miniG$_s$ and MT_2- MT_1-miniG$_s$ complexes

a-e, Structural comparison of MT_1-miniG$_s$, MT_1-G$_i$ (PDB 7DB6) and crystal structure of MT_1 (PDB 6ME2), on (a) overall receptor, (b) ligand binding site, (c) PIF motif, (d) N(D)RY motif and (e) Na^+ binding site and NPxxY motif.

f-j, Structural comparison of MT_2-MT_1-miniG$_s$, MT_2-G$_i$ (PDB 7VH0) and crystal structure of MT_2 (PDB 6ME9), on (f) overall receptor, (g) ligand binding site, (h) PIF motif, (i) N(D)RY motif and (j) Na^+ binding site and NPxxY motif.

k-m, Structural comparison of miniG$_s$ protein among MT_1-miniG$_s$ (yellow), β_2AR-G$_s$ (green; PDB 3SN6) and GDP bound G$_s$ (gray; PDB 6EG8), focusing on (i) overall structure, (j) α5 helix and (k) TCAT motif.

n-p, Structural comparison of G$_s$ protein among MT_2-MT_1-miniG$_s$, β_2AR-G$_s$ (PDB 3SN6) and GDP-bound inactive state (PDB 6EG8), on (n) overall region, (o) α5 helix region and (p) GDP/GTP binding site.

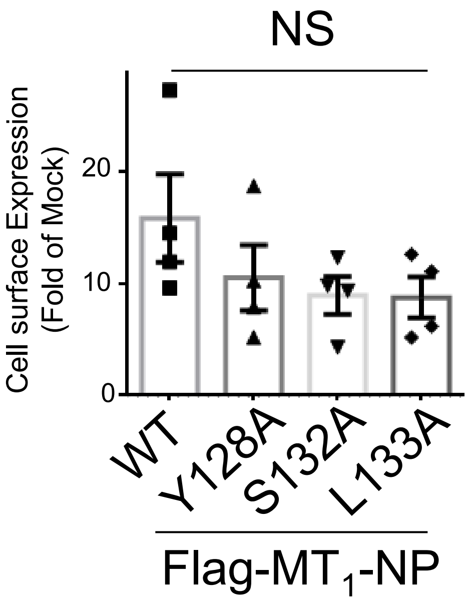

Supplementary Fig. 8. Cell Surface Expression of MT_1–WT or mutants.

Cell surface expression of Flag-tagged human WT or mutant MT_1 was determined by ELISA. Data represent means ± SEM from four independent experiments performed in duplicate (n=4). Statistical analysis was performed by Kruskal-Wallis test followed by Dunn's multiple comparison test. NS; $p > 0.05$

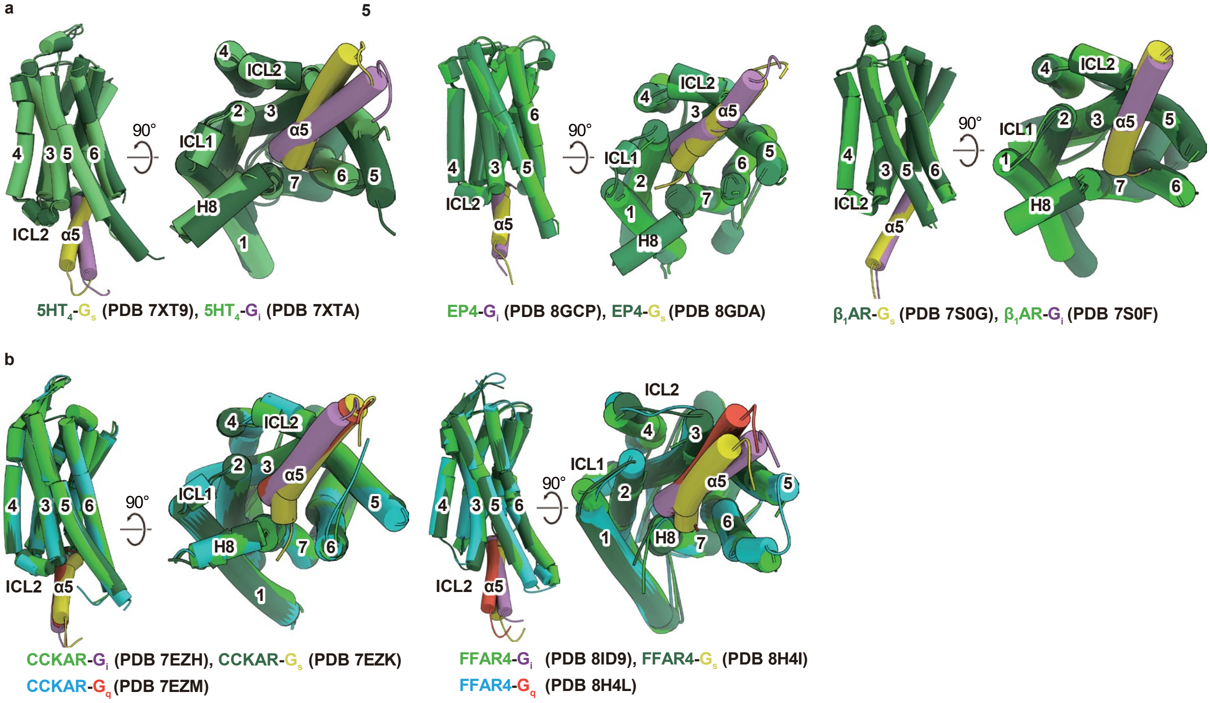

Supplementary Fig. 9 Structural comparison of GPCR-$G_{s/i}$ with the same receptor

a-b, Structural comparison among reported GPCR-G complexes with the same receptor showing (a) primarily G_s coupling receptors including $5HT_4$ (PDB 7XT9 and 7XTA), $β_1$AR (PDB 7S0G and 7S0F), EP4 (PDB 8GCP and 8GDA), or (b) primarily G_q coupling receptors including CCKAR (PDB 7EZM, 7EZH and 7EZK) and FFAR4 (PDB 8H4L, 8ID9 and 8H4I).

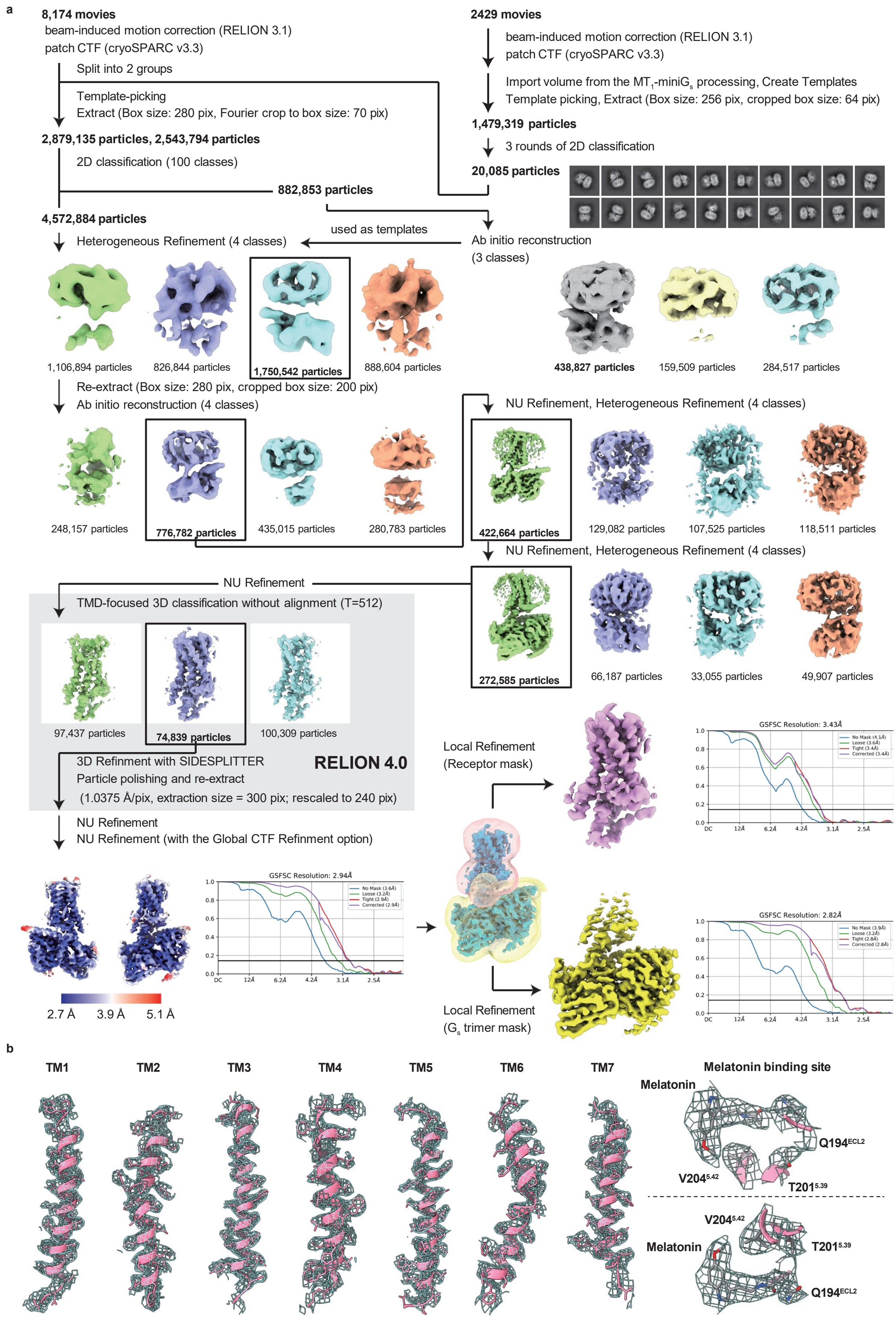

Supplementary Fig. 10 Cryo-EM analysis of the MT_2 chimera-miniG$_s$ complex

(a) The workflow of Cryo-EM analysis of MT_2 chimera-miniG$_s$ complex using RELION 3.1, RELION 4.0 and cryoSPARC v3.3

(b) The structure of MT_2 chimera-miniG$_s$ complex in cryo-EM map at TMD and melatonin binding site of MT_2 chimera.

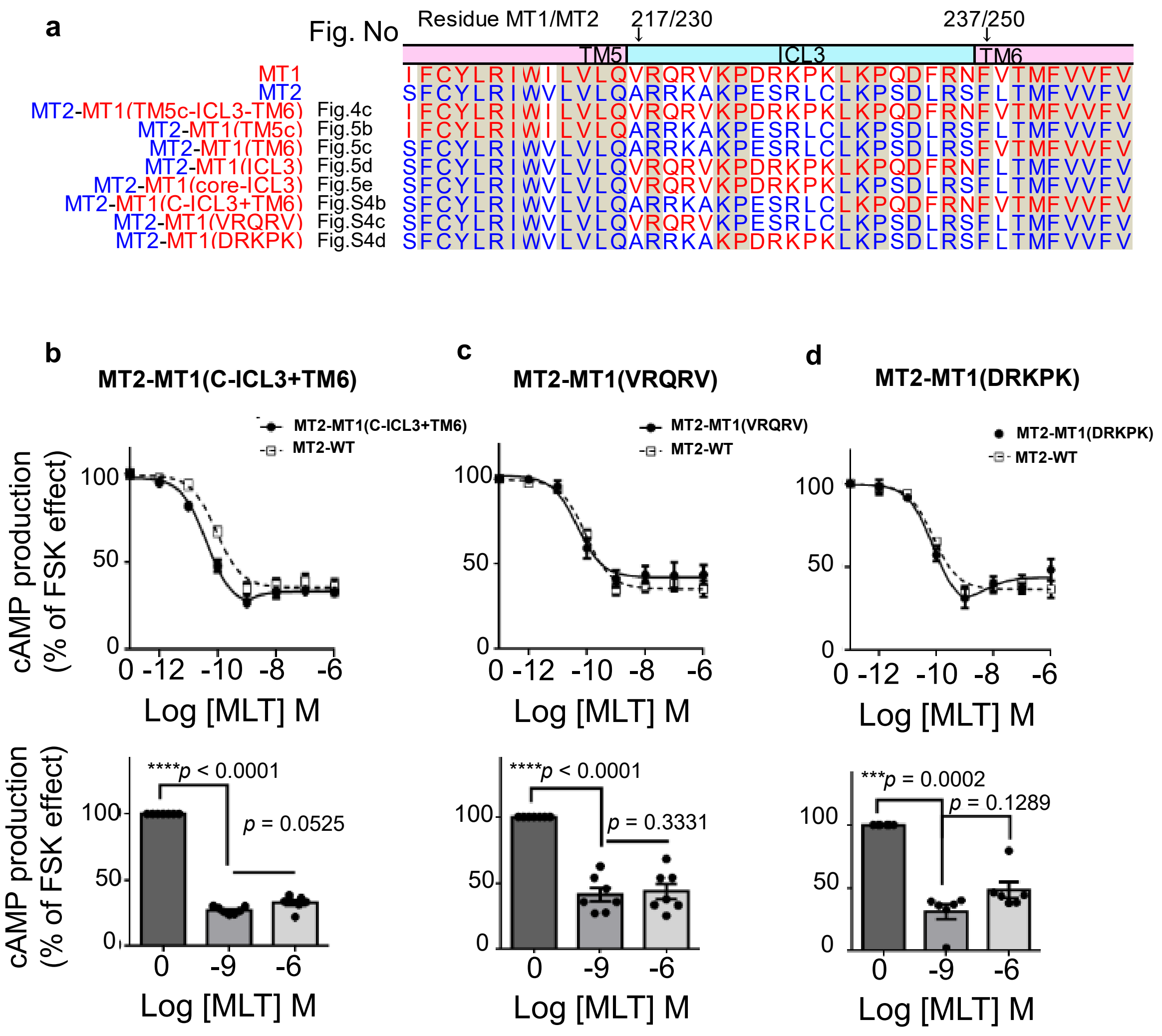

Supplementary Fig.11 Melatonin-induced cAMP response of MT2-ICL3 chimera.
(a) Amino acid sequences alignment of MT2 chimeric receptors (b-d) (upper) Melatonin-induced cAMP production measured by CAMYEL-BRET sensor in transfected HEK293T cells. (lower) Statistical analysis between MLT 0nM and 1nM and 1uM. Data represent means ± SEM from 6 or 7 independent experiments performed in duplicate (n = 7 for (b),(c), n = 6 for (d)). Statistical analysis was performed by One-Way ANOVA followed by Bonferroni's multiple comparison test.

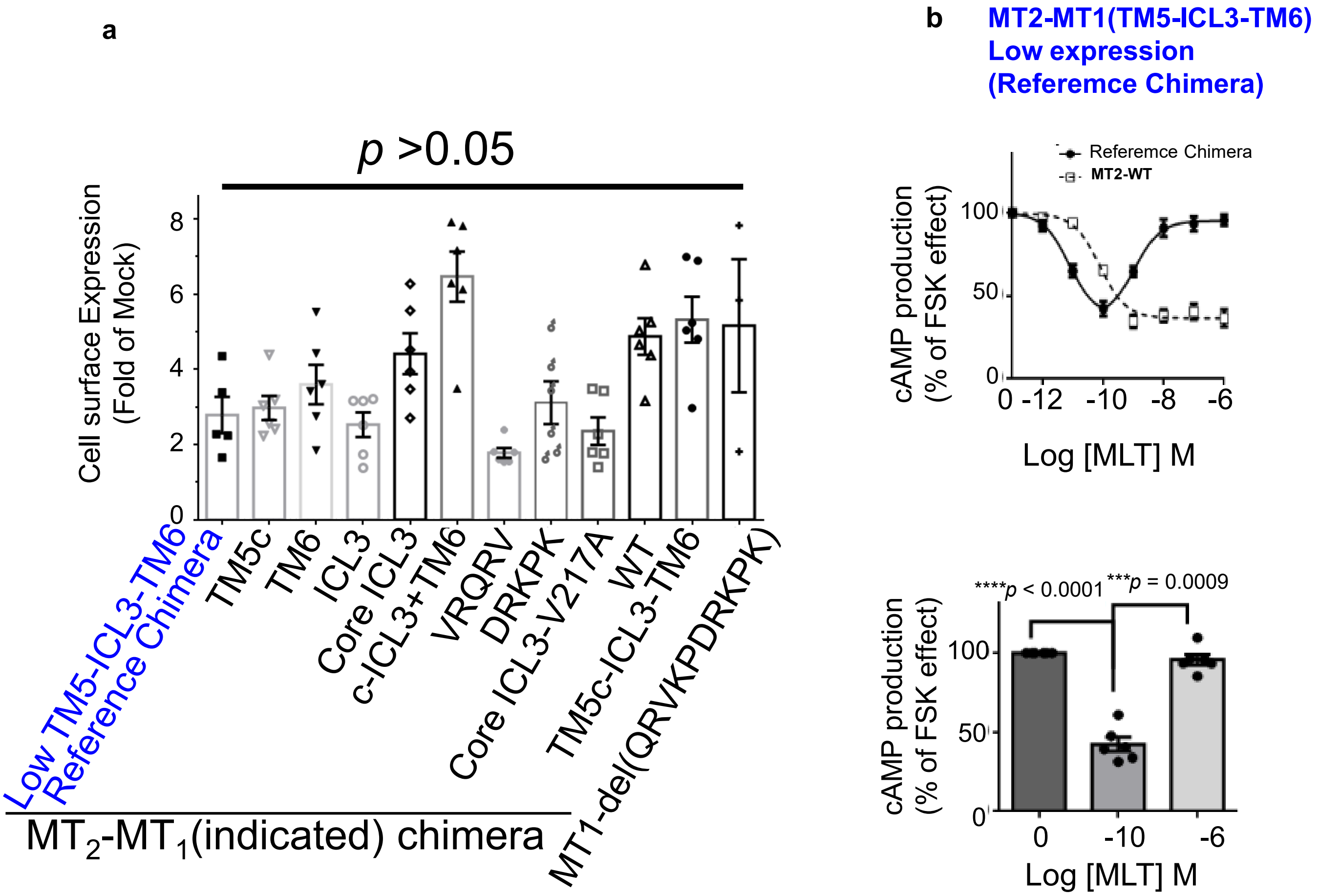

Supplementary Fig.12 Cell Surface Expression of MT2 -MT1 Chimera protein and MT1-deletion mutant.

(a) Cell surface expression of Flag-tagged human MT2-WT or chimera MT2 or MT1-deletion mutant were determined by ELISA. The MT2-MT1(TM5-ICL3-TM6) chimera expressed at low levels was used as a reference. Data represent means ± SEM from at least three independent experiments performed in duplicate. Statistical analysis was performed by Kruskal-Wallis test followed by Dunn's multiple comparison test compared with reference chimera. (b) (upper) Concentration-response curve of reference chimera used in panel (a) (MT2-MT1(TM5-ICL3-TM6)). (lower) Statistical analysis between MLT 0nM and 1nM and 1uM. Data represent means ± SEM from 6 independent experiments performed in duplicate (n = 6). Statistical analysis was performed by One-Way ANOVA followed by Bonferroni's multiple comparison test.

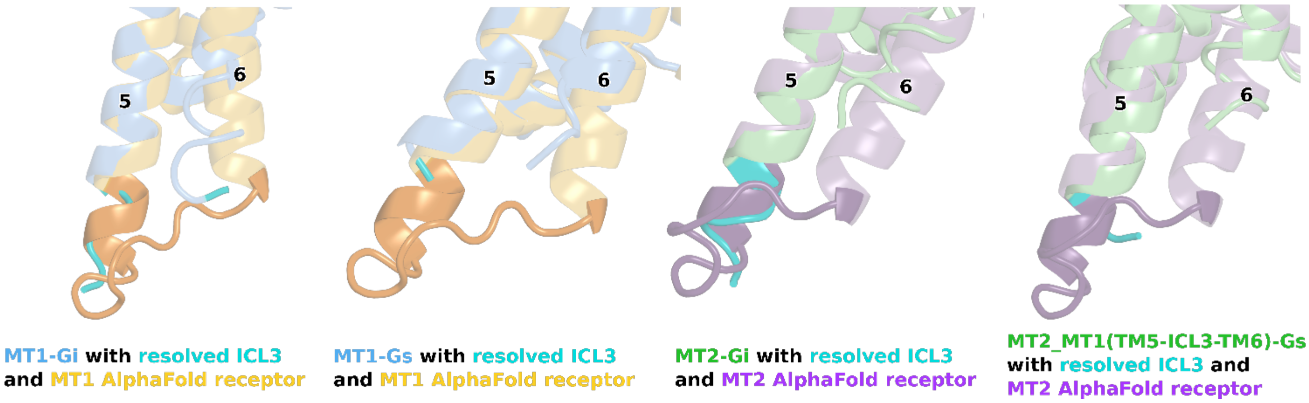

Supplementary Fig. 13 Comparison of AlphaFold-predicted ICL3 conformations with cryo-EM resolved structures of the melatonin receptor-G protein complexes. Overlay of AlphaFold models with corresponding cryo-EM structures for MT1-Gi (PDB 7DB6), MT1-Gs, MT2-Gi (PDB 7VH0), and MT2-MT1(TM5-ICL3-TM6)-Gs complexes. The cryo-EM resolved receptor structures are shown in blue (MT1) and light green (MT2), with the experimentally resolved portions of ICL3 highlighted in bright cyan. AlphaFold-modelled ICL3 regions are shown in dark orange (MT1) and purple (MT2). TM5 and TM6 are labelled for orientation. The AlphaFold ICL3 conformations show reasonable agreement with the resolved portions of the cryo-EM structures, supporting their use in molecular dynamics simulations to investigate ICL3-G protein interactions.

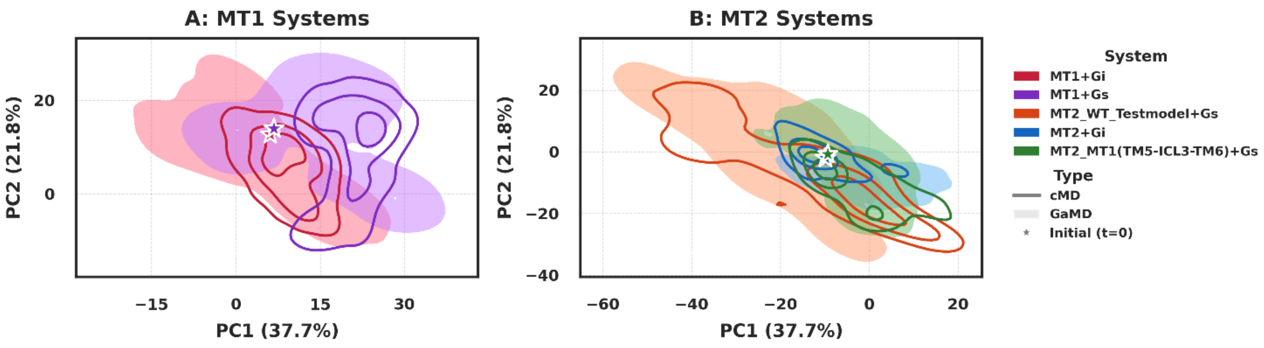

Supplementary Fig. 14. Principal Component Analysis reveals distinct ICL3 conformational ensembles sampled during conventional MD (cMD) and Gaussian accelerated MD (GaMD) simulations across the melatonin receptor-G protein complexes.

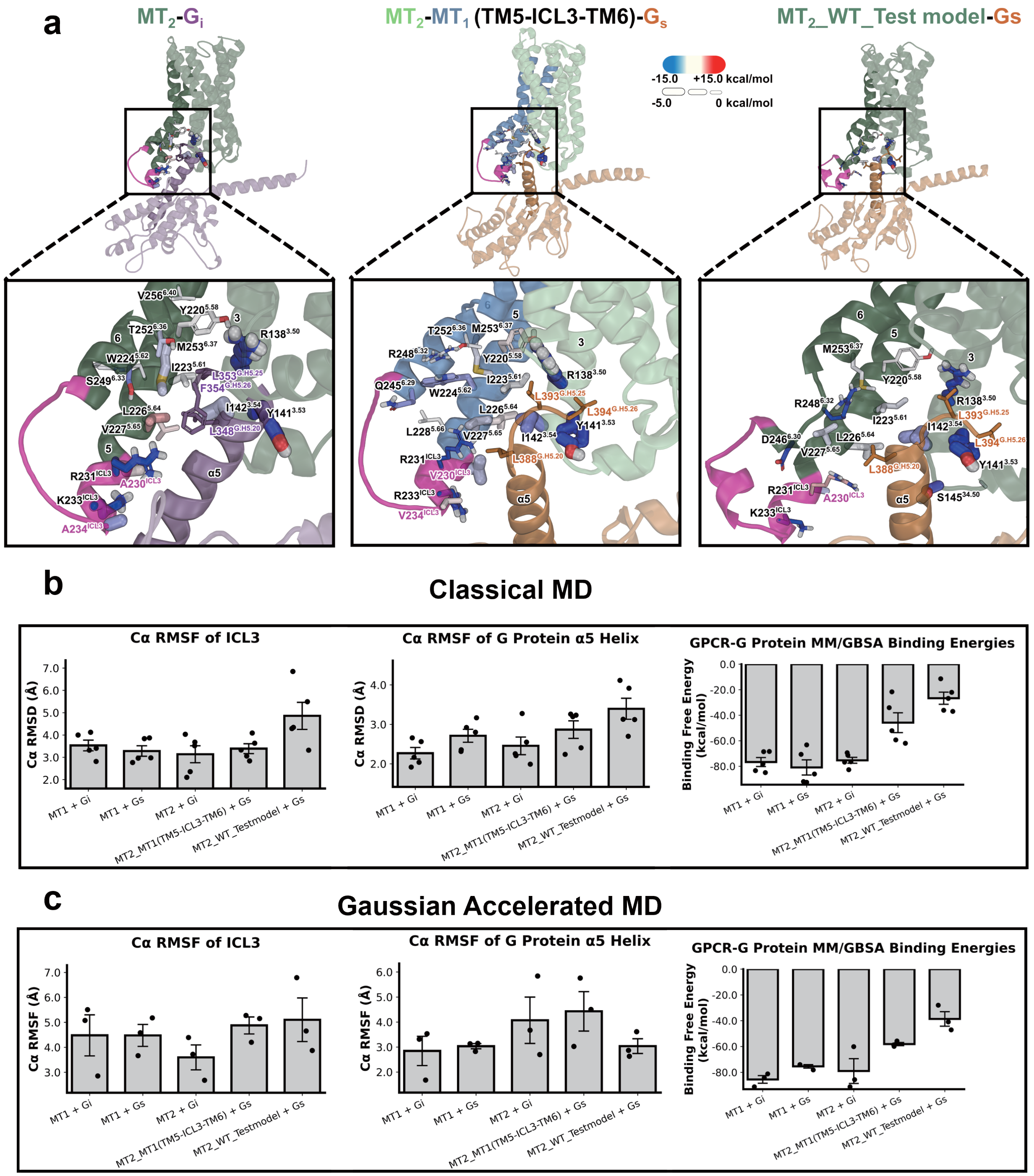

Supplementary Fig. 15 Molecular dynamics simulations support differential G protein binding modes and ICL3-dependent G_s coupling selectivity in the melatonin receptors.

a, Representative MD snapshots of melatonin receptor-G protein complexes. Carbon colours indicate electrostatic energies; stick thickness represents van der Waals interactions with α5 helix. Residues with >1 kcal/mol interactions in TM5/TM6/ICL3 are shown (some removed for clarity). AlphaFold3-modeled ICL3 is in pink and TM5/TM6 are in darker blue. Key contacts: $V217^{ICL3}$ with $L388^{G.H5.20}$ (G_s shallow binding) versus $I210^{5.61}$ with $L348^{G.H5.20}$, $F354^{GH5.26}$, $L353^{GH5.25}$ (Gi deep binding). MT_2-G_s test model shows compromised interactions from alanine substitutions. b, Cα RMSF of ICL3 (left) and G protein α5 helix (middle), and MM/GBSA binding free energies (right) from five independent classical MD simulations (5 × 1 μs each). c, Same analyses from three independent GaMD simulations (3 × 1 μs each). Data: mean ± SEM (n=3).

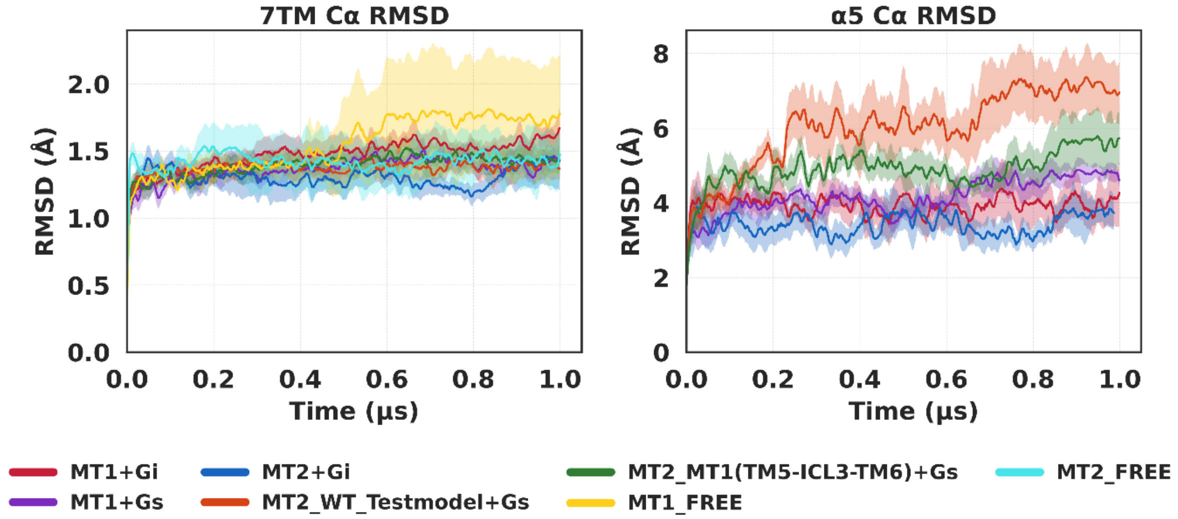

Supplementary Fig. 16 RMSD analysis of the melatonin receptor-G protein complexes from classical MD simulations. RMSD of Cα atoms for the 7-transmembrane domain (7TM), and α-helix 5 (α5) over 1 μs simulations. Systems analyzed: MT1+Gi (dark red), MT1+Gs (purple), MT2+Gi (blue), MT2_WT_Testmodel+Gs (orange), and MT2_MT1(TM5-ICL3-TM6)+Gs chimera (green). Solid lines represent mean values; shaded regions indicate standard deviation.

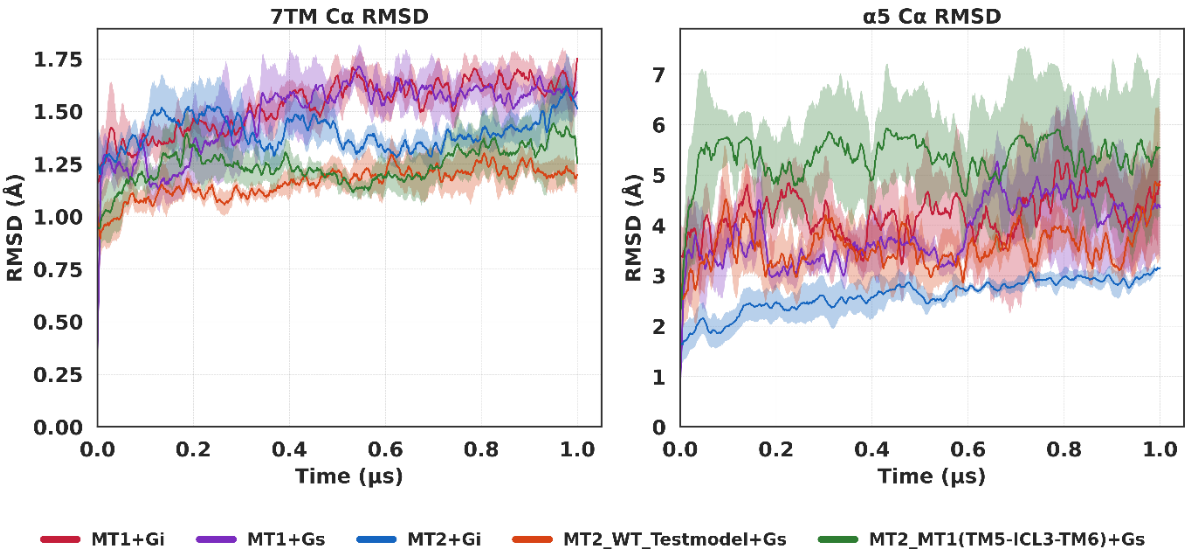

Supplementary Fig. 17 RMSD analysis of the melatonin receptor-G protein complexes from GaMD simulations. RMSD of Cα atoms for the 7-transmembrane domain (7TM), and α-helix 5 (α5) over 1 μs simulations. Systems analyzed: MT1+Gi (dark red), MT1+Gs (purple), MT2+Gi (blue), MT2_WT_Testmodel+Gs (orange), and MT2_MT1(TM5-ICL3-TM6)+Gs chimera (green). Solid lines represent mean values; shaded regions indicate standard deviation.

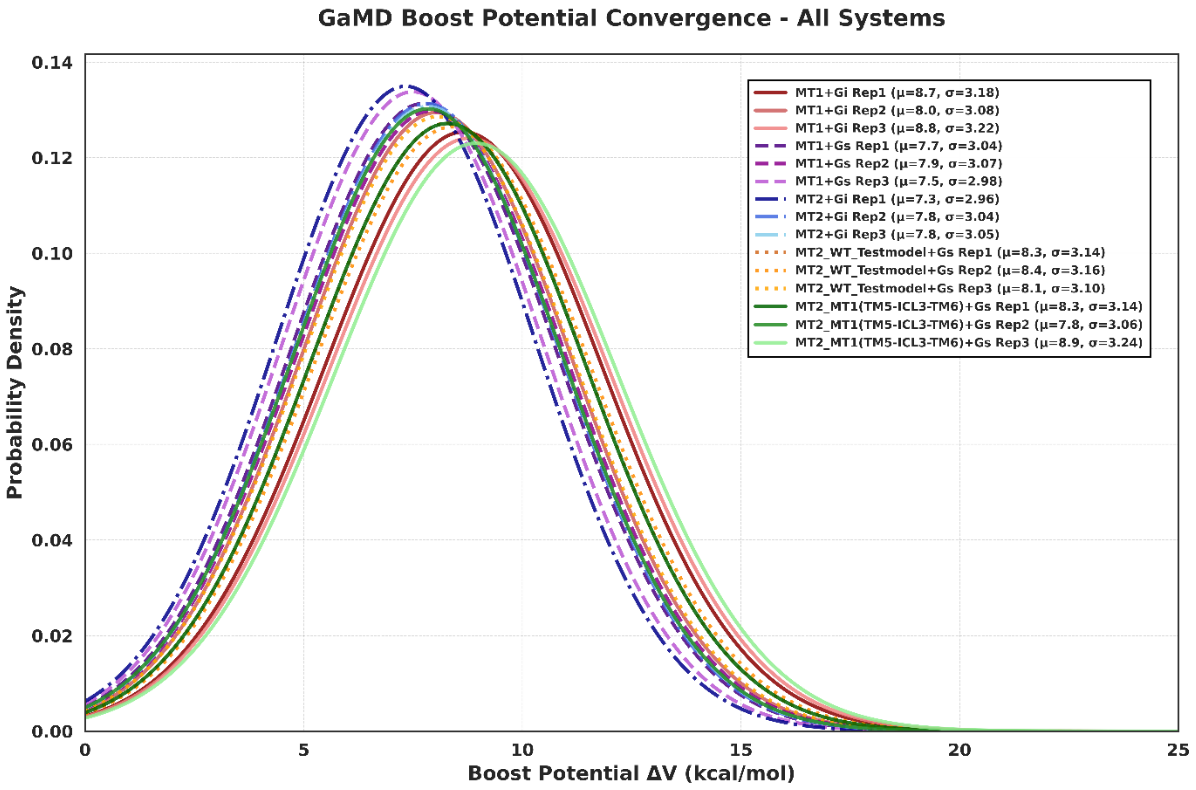

Supplementary Fig. 18 GaMD boost potential distributions. Probability density of boost potential (ΔV) for all GaMD simulation replicates. Overlapping Gaussian distributions across three replicates per system (μ = 7.3-8.9 kcal/mol) demonstrate convergence and reproducibility of enhanced sampling simulations.

Systems	7TM Cα RMSD, Å	7TM Cα RMSF, Å	ICL3 Cα RMSD, Å	ICL3 Cα RMSF, Å	α5 Cα RMSD, Å	α5 Cα RMSF, Å
**Classical MD**						
**MT1_free**	1.54 ± 0.24	1.11 ± 0.14	10.94 ± 0.26[a]	7.71 ± 0.58[a]	-	-
**MT2_free**	1.42 ± 0.18	1.09 ± 0.08	8.54 ± 1.10[b]	6.45 ± 0.25[b]	-	-
**MT1+Gi**	1.48 ± 0.05	0.77 ± 0.03	5.99 ± 0.33[a]	3.53 ± 0.24[a]	3.95 ± 0.32	2.27 ± 0.15
**MT1+Gs**	1.36 ± 0.05	0.69 ± 0.03	7.67 ± 0.43[a]	3.28 ± 0.24[a]	4.33 ± 0.15	2.71 ± 0.16
**MT2+Gi**	1.35 ± 0.11	0.78 ± 0.05	5.03 ± 0.49[b]	3.14 ± 0.38[b]	3.61 ± 0.26	2.46 ± 0.22
**MT2_MT1(TM5-ICL3-TM6)+Gs**	1.40 ± 0.06	0.73 ± 0.05	6.02 ± 0.29[c]	3.39 ± 0.22[c]	5.07 ± 0.24	2.87 ± 0.23
**MT2_WT_Testmodel+Gs**	1.44 ± 0.13	0.77 ± 0.08	8.14 ± 0.81[b]	4.86 ± 0.60[b]	6.22 ± 0.66	3.40 ± 0.27
**GaMD**						
**MT1+Gi**	1.53 ± 0.04	1.18 ± 0.02	5.40 ± 0.97[a]	4.48 ± 0.82[a]	4.21 ± 0.69	2.84 ± 0.59
**MT1+Gs**	1.49 ± 0.10	1.17 ± 0.04	6.03 ± 0.48[a]	4.48 ± 0.44[a]	3.84 ± 0.27	3.03 ± 0.11
**MT2+Gi**	1.40 ± 0.04	1.06 ± 0.05	4.97 ± 0.21[b]	3.60 ± 0.50[b]	2.61 ± 0.20	4.07 ± 0.93
**MT2_MT1(TM5-ICL3-TM6)+Gs**	1.24 ± 0.09	0.97 ± 0.05	6.46 ± 0.26[c]	4.88 ± 0.34[c]	5.44 ± 0.99	4.43 ± 0.79
**MT2_WT_Testmodel+Gs**	1.16 ± 0.05	0.94 ± 0.03	7.33 ± 1.15[b]	5.10 ± 0.87[b]	3.56 ± 0.16	3.04 ± 0.30

Supplementary Table 1. Root-mean-square deviation (RMSD) and root-mean-square fluctuation (RMSF) data for all simulated melatonin receptor systems. Data were calculated from three 1 μs replicas for MT1_free and MT2_free, and five 1 μs replicas for MT1+Gi, MT1+Gs, MT2+Gi, MT2_MT1(TM5-ICL3-TM6)+Gs, and MT2_WT_Testmodel+Gs from classical MD simulations, and three 1 μs replicas for MT1+Gi, MT1+Gs, MT2+Gi, MT2_MT1(TM5-ICL3-TM6)+Gs, and MT2_WT_Testmodel+Gs from GaMD simulations. RMSD is measured against the starting point of simulations (after minimization) by Cα atoms of 7-transmembrane bundle residues for alignment. RMSD 7TM-Cα: 7-transmembrane bundle residues - MT1 systems (S28–R54, F65–V84, S103–Y128, L145–N162, I189–Q216, F234–G258, V278–Y295), MT2-based systems (A42–V65, F78–I101, A115–Y139, P158–P174, Y200–A230, F257–L272, F290–I306), RMSD ICL3: intracellular loop 3 residues (V217–P231[a], A230–P244[b], V230–P244[c]), RMSD Alpha5: G protein α5 helix residues (T327–F354 for Gi systems, D368–L394 for Gs systems). RMSF for melatonin receptor regions is measured upon alignment to the average frame of simulations by Cα atoms of 7-transmembrane bundle residues, with regional measurements conducted using the same structural definitions as RMSD calculations.

System	Box Dimensions (Å)	Total Atoms	Water Molecules	Ions (Na^+/Cl^-)	NaCl Conc.	POPC Lipids (upper/lower)
**MT1+Gi**	100.23 × 100.23 × 185.11	215200	41,532	112/119	~150 mM	129/128 (257 total)
**MT1+Gs**	100.09 × 100.09 × 184.60	213569	40,640	109/133	~150 mM	127/128 (255 total)
**MT2+Gi**	115.15 × 115.15 × 180.59	277596	54,104	147/150	~150 mM	172/175 (347 total)
**MT2_MT1(TM5-ICL3-TM6)+Gs**	100.20 × 100.20 × 186.99	216482	41,432	112/130	~150 mM	128/125 (253 total)
**MT2_WT_Testmodel+Gs**	100.20 × 100.20 × 187.40	217439	41,650	112/129	~150 mM	128/126 (254 total)

Supplementary Table 2. MD Simulation System Composition

Data collection	MT1-miniGs<br>PDB: 11OD<br>EMD-75880 (Overall)<br>EMD-76180 (TMD)<br>EMD-76181 (G protein)	MT2-MT1-miniGs<br>PDB: 11OZ<br>EMD-75909 (Overall)<br>EMD-76182 (TMD)<br>EMD-76183 (G protein)
Microscope	Titan Krios (Thermo Fisher Scientific)	
Voltage (keV)	300	
Electron exposure ($e^-/Å^2$)	50	
Detector	Gatan K3 summit camera (Gatan)	
Magnification	×105,000	
Defocus range (μm)	-0.8 ~ -1.6	
Pixel size (Å/pix)	0.83	
Number of movies	10,373	8,174
Symmetry	C1	
Picked particles	8,196,012	54,229,298
Final particles	85,855	74,839
Map resolution (Å)	3.03 / 3.24 / 2.90<br>(Overall / Receptor focused / G protein focused refinement)	2.94 / 3.43 / 2.82<br>(Overall / Receptor focused / G protein focused refinement)
FSC threshold	0.143	
**Model refinement**		
Atoms	8,040	7,956
**R.m.s. deviations for ideal**		
Bond lengths (Å)	0.002	0.004
Bond angles (°)	0.543	0.793
Validation		
Clash score	9	9
**Ramachandran plot**		
Favored (%)	93.4	94.79
Allowed (%)	6.4	5.21
Outlier (%)	0	0

Supplementary Table 3: Cryo-EM data collection, refinement and validation statistics.

Reliability and reproducibility checklist for molecular dynamics simulations<br>*All boxes must be marked YES by acceptance unless an N/A option is available	Yes	N/A	Response<br>(Please state where this information can be found in the text)
**1. Convergence of simulations and analysis**			
1a. Is an evaluation presented in the text to show that the property being measured has equilibrated in the simulations (*e.g.* time-course analysis)?	☒		Supplementary Table 1<br>Supplementary Fig. 16<br>Supplementary Fig. 17
1b. Then, is it described in the text how simulations are split into equilibration and production runs and how much data were analyzed from production runs?	☒		Methods - Computer Simulations
1c. Are there at least 3 simulations per simulation condition with statistical analysis?	☒		Methods - Computer Simulations<br>Classical MD – 5 replicates<br>GaMD – 3 replicates
1d. Is evidence provided in the text that the simulation results presented are independent of initial configuration?	☒		Supplementary Table 1
**2. Connection to experiments**			
2a. Are calculations provided that can connect to experiments (*e.g.* loss or gain in function from mutagenesis, binding assays, NMR chemical shifts, J-couplings, SAXS curves, interaction distances or FRET distances, structure factors, diffusion coefficients, bulk modulus and other mechanical properties, *etc.*)?	☒		Results - ICL3 of MT1 supports coupling to Gs but not Gi
**3. Method choice**			
3a. Is it described in the text what force field and water model are used and why?	☒		Methods - Computer Simulations<br><br>This force field combination was selected based on current best practices for membrane-embedded GPCR simulations, with FF19SB and OPC providing improved conformational sampling and solvent properties compared to older force fields.
3b. Do simulations contain membranes, membrane proteins, intrinsically disordered proteins, glycans, nucleic acids, polymers, or cryptic ligand binding?	☒	☐	Methods - Computer Simulations<br><br>The simulations contain membranes (POPC bilayer), membrane proteins (GPCRs + G proteins), intrinsically disordered regions (ICL3), and a ligand (melatonin). They do NOT contain glycans, nucleic acids, polymers, or cryptic ligand binding sites.

Supplementary Table 4: MD simulation check list (Continued on next page)

	Reliability and reproducibility checklist for molecular dynamics simulations<br>*All boxes must be marked YES by acceptance unless an N/A option is available	Yes	N/A	Response<br>(Please state where this information can be found in the text)
	If 3b is **YES**, are enhanced sampling methods used?	☒	☐	Methods - Computer Simulations<br><br>Yes, enhanced sampling (GaMD) was appropriately used for systems containing flexible/disordered regions.
	If enhanced sampling methods are used, are the convergence criteria clearly stated?	☒		Methods - Computer Simulations<br><br>Simulations were considered equilibrated when Cα RMSD of the 7TM bundle stabilized within 1.5 Å for at least 100 ns.<br><br>Convergence of GaMD simulations was assessed by monitoring boost potential distributions and free energy profiles (Supplementary Fig. 18).
	If 3b is **YES**, is it explained in the text why or why not enhanced sampling methods are used?	☒		Methods - Computer Simulations
**4. Code and reproducibility**				
4a. Is a table provided describing the system setup, such as simulation box dimensions, total number of atoms, total number of water molecules, salt concentration, lipid composition (number of molecules and type)?		☒		Methods - Computer Simulations<br><br>Yes, Supplementary Table 2.
4b. Is it described in the text what simulation and analysis software and which versions are used?		☒		Methods - Computer Simulations
4c. Are initial coordinate and simulation input files and a coordinate file of the final output provided as supplementary files or in a public repository?		☒		**10.5281/zenodo.17107103**
4d. Is there custom code or custom force field parameters?		☐	☒	Response not needed if **N/A**
	If **YES**, are they provided as supplementary profiles or in a public repository?	☐		

Supplementary Table 4: MD simulation check list (continued)
