# Supplementary material for: Structural basis and physiological significance of non-canonical Gs coupling to the melatonin MT1 receptor
Source: Nat Commun. 2026 May 21;17:6706. doi: 10.1038/s41467-026-73555-6 (PMC13385403; doi:10.1038/s41467-026-73555-6)
Supplement: Supplementary file 2 — Reporting Summary [file 41467_2026_73555_MOESM2_ESM.pdf]

nature portfolio

Corresponding author(s): Osamu Nureki

Last updated by author(s): 2026/04/24

Reporting Summary

Nature Portfolio wishes to improve the reproducibility of the work that we publish. This form provides structure for consistency and transparency in reporting. For further information on Nature Portfolio policies, see our Editorial Policies and the Editorial Policy Checklist.

Statistics

For all statistical analyses, confirm that the following items are present in the figure legend, table legend, main text, or Methods section.

n/a	Confirmed	
☐	☒	The exact sample size (n) for each experimental group/condition, given as a discrete number and unit of measurement
☐	☒	A statement on whether measurements were taken from distinct samples or whether the same sample was measured repeatedly
☐	☒	The statistical test(s) used AND whether they are one- or two-sided<br>*Only common tests should be described solely by name; describe more complex techniques in the Methods section.*
☒	☐	A description of all covariates tested
☐	☒	A description of any assumptions or corrections, such as tests of normality and adjustment for multiple comparisons
☐	☒	A full description of the statistical parameters including central tendency (e.g. means) or other basic estimates (e.g. regression coefficient) AND variation (e.g. standard deviation) or associated estimates of uncertainty (e.g. confidence intervals)
☐	☒	For null hypothesis testing, the test statistic (e.g. F, t, r) with confidence intervals, effect sizes, degrees of freedom and P value noted<br>*Give P values as exact values whenever suitable.*
☒	☐	For Bayesian analysis, information on the choice of priors and Markov chain Monte Carlo settings
☒	☐	For hierarchical and complex designs, identification of the appropriate level for tests and full reporting of outcomes
☒	☐	Estimates of effect sizes (e.g. Cohen's d, Pearson's r), indicating how they were calculated

Our web collection on statistics for biologists contains articles on many of the points above.

Software and code

Policy information about availability of computer code

Data collection	Tecan infinite F500, Tecan SPARK(Tecan), Mithras LB940(Berthold)<br>SpectraMax M5 (Molecular Devices)<br>BZ-X800 (Keyence)<br>Amersham Imager 600 (Cytiva Lifescience)<br>FUSION SOLO.7S.EDGE (Vilber-Lourmat)<br>Nivo S (PerkinElmer)<br>EPU software (Thermo Fisher Scientific)
Data analysis	GraphPad Prism 6.0 , 8.0, 10.0 and 11.0 software (GraphPad Software Inc.)<br>SpectraMax M5 (Molecular Devices)<br>MyAssays (PerkinElmer)<br>Amersham Imager 600 (Cytiva Lifescience)<br>BZ-X800 (Keyence)<br>ImageJ software v1.52a (NIH)<br>RELION 3.1, cryoSPARC v3.3, SIDESPLITTER, PHENIX (version 1.19-4092), WinCoot (version 0.9.8.7), USCF ChimeraX (version 1.7), Cuemol2 (version 2.2.3.443)

For manuscripts utilizing custom algorithms or software that are central to the research but not yet described in published literature, software must be made available to editors and reviewers. We strongly encourage code deposition in a community repository (e.g. GitHub). See the Nature Portfolio guidelines for submitting code & software for further information.

Data

Policy information about availability of data

All manuscripts must include a data availability statement. This statement should provide the following information, where applicable:

- Accession codes, unique identifiers, or web links for publicly available datasets
- A description of any restrictions on data availability
- For clinical datasets or third party data, please ensure that the statement adheres to our policy

The cryo-EM density maps have been deposited in the Electron Microscopy Data Bank (EMDB) under accession codes EMD-75880 (MT1–miniGs, overall), EMD-76180 (MT1–miniGs, transmembrane domain), EMD-76181 (MT1–miniGs, G protein), EMD-75909 (MT2–MT1–miniGs, overall), EMD-76182 (MT2–MT1–miniGs, transmembrane domain), and EMD-76183 (MT2–MT1–miniGs, G protein). Atomic coordinates have been deposited in the Protein Data Bank (PDB) under accession codes 11OD (MT1–miniGs) and 11OZ (MT2–MT1–miniGs). The molecular dynamics simulation data generated in this study have been deposited in Zenodo at Zenodo entry 17107103 [https://doi.org/10.5281/zenodo.17107103]. No restricted-access datasets are included in this study.
Further information and requests for resources and reagents should be directed to and will be fulfilled by the Lead Contact, Ralf Jockers (ralf.jockers@inserm.fr).
Source data are provided with this paper.

Research involving human participants, their data, or biological material

Policy information about studies with human participants or human data. See also policy information about sex, gender (identity/presentation), and sexual orientation and race, ethnicity and racism.

Reporting on sex and gender	NA
Reporting on race, ethnicity, or other socially relevant groupings	NA
Population characteristics	NA
Recruitment	NA
Ethics oversight	NA

Note that full information on the approval of the study protocol must also be provided in the manuscript.

Field-specific reporting

Please select the one below that is the best fit for your research. If you are not sure, read the appropriate sections before making your selection.

☒ Life sciences ☐ Behavioural & social sciences ☐ Ecological, evolutionary & environmental sciences

For a reference copy of the document with all sections, see nature.com/documents/nr-reporting-summary-flat.pdf

Life sciences study design

All studies must disclose on these points even when the disclosure is negative.

Sample size	(For Cell based assay) Sample sizes was not determined by statistical method. Sample sizes were at least three that is a standard sample size to accurately detect differences in cell biology field.<br>(For Animal experiment) No statistical methods were used to predetermine sample size. The number of mice used was set to N = 6 or more because individual differences are likely to occur. However, in vivo experiment, the observed effect size was large ($\eta^2 = 0.51$), and with these group sizes the statistical power is estimated to be approximately ~0.8 at $\alpha = 0.05$, indicating that the sample size was sufficient to detect biologically meaningful differences. Specifically, CBA/NSlc mice were used at n = 6 per group, and C57BL/6JJmsSlc mice were used at n = 6 per group (except MLT for IHC, n = 7).
Data exclusions	No data were excluded from the analysis
Replication	All experiments were performed in at least biological triplicate with similar results. In the animal experiments, samples were collected in the independent measurement.
Randomization	Inbred mice of the same age and sex were bred in the same environment and used in the experiment. Sampling was performed randomly.
Blinding	No blinding was used in this study

Reporting for specific materials, systems and methods

We require information from authors about some types of materials, experimental systems and methods used in many studies. Here, indicate whether each material, system or method listed is relevant to your study. If you are not sure if a list item applies to your research, read the appropriate section before selecting a response.

Materials & experimental systems

n/a	Involved in the study	
	☒	Antibodies
	☒	Eukaryotic cell lines
☒		Palaeontology and archaeology
	☒	Animals and other organisms
☒		Clinical data
☒		Dual use research of concern
☒		Plants

Methods

n/a	Involved in the study	
☒		ChIP-seq
☒		Flow cytometry
☒		MRI-based neuroimaging

Antibodies

Antibodies used	Alexa Fluor 647 anti-cAMP antibody (1:100; the LANCE cAMP Detection Kit; #AD0262, PerkinElmer, USA).<br>Rabbit anti- phospho-CREB (Ser133) (1:1,000 [WB], 1:500 [IHC]; Cell Signalling Technology Cat#9198)<br>Rabbit anti- CREB (1:1,000; Cell Signalling Technology Cat#9192)<br>HRP-conjugated goat polyclonal antibody against rabbit IgG (1:10,000; Cell Signalling Technology Cat# 7074)<br>Anti-IgG(H+L), Rabbit, Goat-Poly, Biotin (1:1,000; VECTASTAIN Elite; #BA-1000-1.5)
Validation	All antibodies were validated by commercial source. No homemade or previously unpublished antibodies were used in this study. All antibodies were validated using western blotting and/or immunofluorescence staining.

Eukaryotic cell lines

Policy information about cell lines and Sex and Gender in Research

Cell line source(s)	HEK293T cells were purchased from ATCC.<br>Sf9 (Gibco), HEK293F (Thermo Fisher Scientific)
Authentication	HEK293T cells were authentificated by ATCC by STR sequencing and used at low passages.<br>Sf9 and HEK293F are authenticated by the supplier. No additional authentication was performed by the authors in this study.
Mycoplasma contamination	Not tested (Sf9 and HEK293F); tested routinely negative (HEK293T) for mycoplasma contamination in our lab.
Commonly misidentified lines<br>(See ICLAC register)	No commonly misidentified lines were used.

Animals and other research organisms

Policy information about studies involving animals; ARRIVE guidelines recommended for reporting animal research, and Sex and Gender in Research

Laboratory animals	Five-week-old male C57BL/6JJmsSlc mice and CBA/NSlc mice (Japan SLC Inc., Shizuoka, Japan)
Wild animals	This study did not involve wild animals.
Reporting on sex	Only male mice were used to avoid variability associated with the estrous cycle.
Field-collected samples	This study did not involve samples collected from the field.
Ethics oversight	All animal experiments were approved by the Committee of Animal Care and Use of the Aichi Medical University (protocol number 2022-62) and the Kyushu University (protocol number A25-228-1). All experimental procedures were conducted in accordance with the institutional guidelines for the use of experimental animals.

Note that full information on the approval of the study protocol must also be provided in the manuscript.

Plants

Seed stocks	NA
Novel plant genotypes	NA
Authentication	NA
