# Supplementary material for: Structural basis and physiological significance of non-canonical Gs coupling to the melatonin MT1 receptor
Source: Nat Commun. 2026 May 21;17:6706. doi: 10.1038/s41467-026-73555-6 (PMC13385403; doi:10.1038/s41467-026-73555-6)
Supplement: Supplementary file 3 — Transparent Peer Review File [file 41467_2026_73555_MOESM3_ESM.pdf]

nature portfolio

Peer Review File

Structural basis and physiological significance of non-canonical G_s coupling to the melatonin MT_1 receptor

Corresponding Author: Professor Osamu Nureki

Parts of this Peer Review File have been redacted as indicated to remove third-party material.

This file contains all reviewer reports in order by version, followed by all author rebuttals in order by version.

Version 0:

Reviewer comments:

Reviewer #1

(Remarks to the Author)
Review of Oishi et al.

The manuscript by Oishi and co-workers describes the structure of the Melatonin Receptor (MT1) in complex with a minimized Gs G-Protein complex. The work highlights the ability of MT1 to promiscuously activate Gs and Gi and the apparent Gi selectivity of MT2. The manuscript details the potential mechanism that is driven by the ICL3 of MT1 and confirmed by a chimeric genetic construct approach.
In general, the work is well conceived and is an important insight into the melatonin driven circadian rhythms. This reviewer believes there is enough evidence in the manuscript to point out the role of ICL3 in G-protein selectivity. I however have a few minor comments and things that could be addressed to strengthen the work presented.
1. I firstly thank the authors in sharing their raw cryoEM potential maps and models. This is where I think I have the largest concerns. With the MT1:miniGs structure the entire TM5 is out of index, which is the cause of the significant amount of Ramachandran outliers in the structure. As TM5 makes some important contacts with the G-protein, this should be addressed. Also with both MT1 and the MT2 chimeric structure, the entrance into Helix 8 from TM7 is incorrectly modelled and would need to be fixed in both. I'm also uncertain why the authors decided to model more cholesterol molecules in the MT2 hybrid, and how certain are the authors that it is cholesterol and not CHS? My personal view, if I'm uncertain about lipids, unless I have M/S evidence I am generally reluctant to model them.
2. With regards to the LgBIT nLuc assays. While I'm moderately convinced, I'm not certain that the vehicle is the best control. As the NanoBIT interaction can be driven by equilibrium, so the lack of response in the MT2 Gs assays is not necessarily no interaction. However, the cAMP assays with/without PTX go someway to addressing this, but these are secondary messenger assays and not direct coupling assessment. Would it not be worthwhile doing a BRET based TRUPATH assay or something similar to confirm that there is definitely no Gs coupling with MT2?
3. While it seems striking the alpha-5 engagement angle of Gs to MT1 and this could be driven by ICL3 topology, my worry is that the system is not using the more WT Gs (or even and Dominant Negative Gs) and only a small subset of confirmations would be allowed by Nb35 binding. I think it's also worth noting that while the angle of alpha5 engagement is different, it doesn't appear to change the overall binding position of the G-protein trimer, with the G-beta residing in largely the same position as the Gi-heterotrimer.
Some very minor points:
1. Should line 307 read "selectivity" or "enabling Gs coupling"? "Dispensible for Gi coupling" to me suggests that ICL3 is not playing any role there at all which it obviously is….
2. Line 195: The jump from the MT results to GPR61 seems a bit of a logical leap. Could the authors please clarify what they mean here.
3. Figure 2i-2k, these are very minor differences and are probably attributable to overall 3D-variability. As these calculations were not performed, I would urge caution to interpret minor changes in structure in a SPA experiment, especially as we have observed in our work that there is a significant 3D-variation in the G-protein trimers.
4. Line 55 seems a pretty bold claim backed by only a single meta analysis. For example, Class B GPCRs all couple to many different G-proteins, while being predominately Gs coupled, and so do many Class A (CCK, Muscarinics, etc…)

I think the manuscript deserves publication and adds valuable knowledge into the MT1 and MT2 receptor signaling

functions.

Matthew Belousoff, Monash Institute of Pharmaceutical Sciences

Reviewer #2

(Remarks to the Author)
The manuscript by Oishi et al. reports that the melatonin MT1 receptor can engage a non-canonical Gs/cAMP signaling pathway in addition to its canonical Gi signaling. The authors show this with in vitro assays (HEK293T, BRET/HTRF, NanoBiT), ex-vivo PT/MBH slice cultures where 16-h melatonin exposure increases cAMP/pCREB, and an in-vivo experiments in which melatonin injection (ZT8) in mice kept under a long-night schedule (8L:16D) increases PT pCREB measured 16 h later (ZT0). The work also includes cryo-EM structures and MD/chimera experiments that identify ICL3/TM3/ICL2 determinants for MT1–Gs coupling.

1) The in-vivo and ex-vivo work used C57BL/6J mice. C57BL/6 strains are well known to be melatonin-deficient (practically absent nocturnal melatonin rhythm), which may critically affect both baseline PT physiology and response to exogenous melatonin. Please justify the choice of C57BL/6J and discuss whether the observed Gs activation at “dawn” requires an intact endogenous melatonin rhythm. Providing key in-vivo and/or ex-vivo experiments in a melatonin-proficient strain such as the C3H melatonin proficient mice would help clarifying this issue.
2)In line with the previous comment, given that strain and LD schedule determine endogenous melatonin, reporting serum melatonin levels could better provide a picture of the observed effects.
3) In the Methods the authors state that slices for ex-vivo were from mice maintained 12L:12D and treated at ZT12 for 16 h in culture, whereas in-vivo animals were kept under 8L:16D for 3 weeks and received melatonin at ZT8, with tissue collection after 16 h (ZT0). Please explicitly justify the switch from 12L:12D (ex-vivo source) to 8L:16D (in-vivo) — what physiological condition is modelled by 8L:16D? In keeping, is any dynamic Gi-Gs switch expected according to timing and melatonin physiological levels? The authors have included a single time point. At least a comment should be provided. Finally, the sequence of time points used in ex vivo and in vivo experiments (ZT definitions, 6 h post-dawn collection, 16-h incubation, etc.) is scientifically reasonable but not intuitive for non-chronobiologists. I suggest a simple figure highliting light–dark cycles, melatonin exposure, and tissue collection times to greatly improve clarity for non-specialized readership.
4) one key finding is the cAMP/pCREB increase to MT1–Gs coupling in vivo. I would suggest one of the following experiments to prove this finding: use of a selective MT1 antagonist or MT1 KO (or MT1/MT2 double KO) control; demonstration that the signal is abolished by PKA inhibition although very selective compounds are relatively missing; or inclusion of MT1 receptor expression/localization data in PT under the experimental LD conditions of the study.
5) I suggest reporting exactly the n (biological replicates) for each panel in the figures (some panels report n=4 or n=6–7 but be explicit for each experiment). Consider adding power justification for the in-vivo measures.
6) Statistics: The authors report one-tailed p-values for some ex-vivo and in-vivo comparisons (e.g., pCREB analyses). One-tailed tests are acceptable only with strong a-priori directional hypotheses and should be justified. MOreover, t-tests, ANOVAs, Holm–Šidák and Bonferroni corrections have been computed. Please ensure each figure legend explicitly lists the test used, exact n, degrees of freedom (or F/t values), and whether normality was tested (as exmaple using Shapiro–Wilk given the low n per group) and variance equality assessed. Finally, provide exact p-values and corrected p-values consistently.
7) The limitations of the study should be better adressed.
8) The manuscript should be largely revised for typos, grammar and incorrect phrasing. The abbreviations should also be used consistently and cited at the first time of appearence.

Reviewer #3

(Remarks to the Author)
I co-reviewed this manuscript with one of the reviewers who provided the listed reports. This is part of the Nature Communications initiative to facilitate training in peer review and to provide appropriate recognition for Early Career Researchers who co-review manuscripts.

Reviewer #4

(Remarks to the Author)
A very interesting article, worthy of publishing in Nature Communications, on the mechanism and importance of the coupling of the melatonin MT1 receptor to both Gi and Gs. The authors have combined pharmacology, structural biology and computational chemistry in a very effective synergy and have shed some interesting light on the difficult issue of G protein selectivity.
Where it says ‘The physiological relevance of dual Gi/Gs coupling… remains elusive” is a very elegant phrase but it is probably an overstatement that should be toned down, e.g. Weston et al., JBC (2015, 2016) showed how RAMPs affected GPCR Gs/Gi coupling in a ligand dependant manner.
The statement that “with no universal structural determinant emerging from current structures, highlighting the complexity of GPCR-G protein selectivity” (p7, line 213)is a little depressing if we are looking for simple answers, but it is very important and the authors shed considerable light on this topic in this article, e.g. with regard to the proposed (by others) importance of the TM5-TM6 distance (p7, line 22). The most interesting aspects of this work lie in the excellent work on MT1-MT2 chimeras (including a cryo-EM structure), which point to the importance of ICL3. There is a very long history on the importance of ICL3

in G protein coupling that should be noted, but I think this is significant and well-validated.
The modelling of ICL3 is discussed on p9. It is refered to as a short loop – but at 20 residues that is not the case. I would accept relatively short rather than short - its still in the region difficult to model and I'm not convinced we can fully trust AlphaFold in this type of modelling.
Generally the modelling is excellent, and provides valuable supporting information. However, I have concerns about modelling the interaction energies. NAMDenergy is simply a molecular mechanics energy and as such does not take into account entropy, which is important for systems such as this that involve hydrophobic interactions, and so the current calculations are not acceptable - the work should be repeated, e.g. with entropy taken into account with a surface area term, e.g. as in MMGBSA.

Minor comments
P2 line 55. Rarely co-couple to Gi/o proteins or CO-COUPLE with a large…. Would add clarity
P6 line 187 "I" should be "is"
P7 line 213. The authors point out the possible problems of using engineered G proteins. Can the authors confirm that their G proteins were not engineered and so there are no hidden problems arising from that?
P13 line 427. no need for space before degrees C - it's not an SI unit

Version 1:

Reviewer comments:

Reviewer #1

(Remarks to the Author)
I think the authors have done a commendable job with revising their manuscript. It is my opinion that it is greatly strengthened by the extra hard work which was undertaken. I am completely satisfied that my concerns were addressed. It is my recommendation that the manuscript is more than appropriate for publication in its revised state.

Kind Regards

Matthew Belousoff, Monash Institute of Pharmaceutical Sciences

Reviewer #2

(Remarks to the Author)
The authors have addressed my concerns and produced new data to support their findings and hypothesis.
I just suggest including the protocol numbers for the ethical approvals of the animal experiments at line 496.

Reviewer #3

(Remarks to the Author)
I co-reviewed this manuscript with one of the reviewers who provided the listed reports. This is part of the Nature Communications initiative to facilitate training in peer review and to provide appropriate recognition for Early Career Researchers who co-review manuscripts.

Reviewer #4

(Remarks to the Author)
I am happy that the authors have done an excellent job in addressing my concerns so I am happy for the manuscript to be published with no further changes required

Open Access This Peer Review File is licensed under a Creative Commons Attribution 4.0 International License, which permits use, sharing, adaptation, distribution and reproduction in any medium or format, as long as you give appropriate credit to the original author(s) and the source, provide a link to the Creative Commons license, and indicate if changes were made.
In cases where reviewers are anonymous, credit should be given to 'Anonymous Referee' and the source.
The images or other third party material in this Peer Review File are included in the article's Creative Commons license, unless indicated otherwise in a credit line to the material. If material is not included in the article's Creative Commons license and your intended use is not permitted by statutory regulation or exceeds the permitted use, you will need to obtain permission directly from the copyright holder.
To view a copy of this license, visit https://creativecommons.org/licenses/by/4.0/

REVIEWER COMMENTS
Reviewer #1 (Remarks to the Author):
Review of Oishi et al.

The manuscript by Oishi and co-workers describes the structure of the Melatonin Receptor (MT1) in complex with a minimized Gs G-Protein complex. The work highlights the ability of MT1 to promiscuously activate Gs and Gi and the apparent Gi selectivity of MT2. The manuscript details the potential mechanism that is driven by the ICL3 of MT1 and confirmed by a chimeric genetic construct approach.
In general, the work is well conceived and is an important insight into the melatonin driven circadian rhythms. This reviewer believes there is enough evidence in the manuscript to point out the role of ICL3 in G-protein selectivity. I however have a few minor comments and things that could be addressed to strengthen the work presented.

1. I firstly thank the authors in sharing their raw cryoEM potential maps and models. This is where I think I have the largest concerns.

With the MT1:miniGs structure the entire TM5 is out of index, which is the cause of the significant amount of Ramachandran outliers in the structure. As TM5 makes some important contacts with the G-protein, this should be addressed. Also with both MT1 and the MT2 chimeric structure, the entrance into Helix 8 from TM7 is incorrectly modelled and would need to be fixed in both.
Response: We appreciate your suggestion on modeling of these regions and totally agree with your point and therefore refined the model. Especially for H8, the density is too blur to model precisely, so we decided not to model this region and exclude the discussion on this part. We just attached refined PDB models with cryoEM maps, in which you can see the modifications. We uploaded the zip file which contains these updated data. A following link to the data storage location is also provided.
https://ucsfonline-my.sharepoint.com/:f:/g/personal/hiroyuki_okamoto_ucsf_edu/IgAs0GnLurz0QaEecR8QTS1IAfjC0ompaLMhyxJ9vXApwQc?e=NI0X91

I'm also uncertain why the authors decided to model more cholesterol molecules in the MT2 hybrid, and how certain are the authors that it is cholesterol and not CHS? My personal view, if I'm uncertain about lipids, unless I have M/S evidence I am generally reluctant to model them.
Response: We also agree with your suggestion here. As we didn't perform mass spectroscopy experiments, we eliminated the CHS molecule from the model. Same as the last point, we just attached refined PDB models with cryoEM maps, in which you can see the modifications (Please see the uploaded zip file or the link above).

2. With regards to the LgBIT nLuc assays. While I'm moderately convinced, I'm not certain that the vehicle is the best control. As the NanoBIT interaction can be driven by equilibrium, so the lack of response in the MT2 Gs assays is not necessarily no interaction. However, the cAMP assays with/without PTX go someway to addressing

this, but these are secondary messenger assays and not direct coupling assessment. Would it not be worthwhile doing a BRET based TRUPATH assay or something similar to confirm that there is definitely no Gs coupling with MT2?

Response: We appreciate the reviewer's thoughtful suggestion. Following the recommendation of the reviewer we replicated Gi and Gs coupling to MT1 and MT2 in a BRET recruitment assay. As a positive benchmark for constitutive Gs coupling, we included GPR62, which has been reported to constitutively activate the Gs/cAMP pathway (Oishi et al., Sci Rep 2017). In our BRET miniGs assay, GPR62 showed clear basal miniGs recruitment (80mBu), whereas MT2 did only display a basal signal of 15mBu (Supplementary Fig. S2a), which did not change with increasing melatonin concentrations at comparable donor and acceptor expression levels between the GPR62 and MT2 settings (Supplementary Fig. S2b,c). This shows that constitutive Gs coupling is measurable with the BRET assay with GPR62, but no constitutive and ligand-induced Gs recruitment was seen for MT2.

Regarding the concern with NanoBit-complementation assay, complementary BRET experiments confirm the Nano-Bit results. BRET assays readily detected melatonin-induced miniGi-Venus recruitment to MT1-Rluc and MT2-Rluc as well as miniGs-Venus recruitment to MT1-Rluc with (pEC50: 9.31 ± 0.365 (Supplementary Fig.S1d-f), confirming our initial results obtained with the split Nanoluc complementation assay (Fig 1c-f).

The new part in the Results section reads in page4 Line115-119 as follows:

Melatonin-induced G protein profiles were replicated in complementary BRET assays (Supplementary Fig.S2a-d). In addition, BRET assays did not provide evidence for constitutive Gs coupling to MT2, which was readily detectable for GPR62, which has been reported to constitutively activate the Gs/cAMP pathway [22] (Supplementary Fig.S2d-f).

3. While it seems striking the alpha-5 engagement angle of Gs to MT1 and this could be driven by ICL3 topology, my worry is that the system is not using the more WT Gs (or even and Dominant Negative Gs) and only a small subset of confirmations would be allowed by Nb35 binding. I think it's also worth noting that while the angle of alpha5 engagement is different, it doesn't appear to change the overall binding position of the G-protein trimer, with the G-beta residing in largely the same position as the Gi-heterotrimer.

Response: We agree with the reviewer's comment, as we could see uniform conformation when we overlay G protein trimer from MT1-Gi (PDB (7DB6), MT1-Gs (this study) and MT2-MT1-Gs (this study)). We acknowledge that we cannot exclude the possibility you mentioned that Nb35 (or scFv16) restrict the conformations of α5 helix. Nevertheless, the overlay of the complex still reveals some differences in α5 helix orientation Motivated by this observation we further investigated ICL3 contribution to Gs coupling by MD simulations.

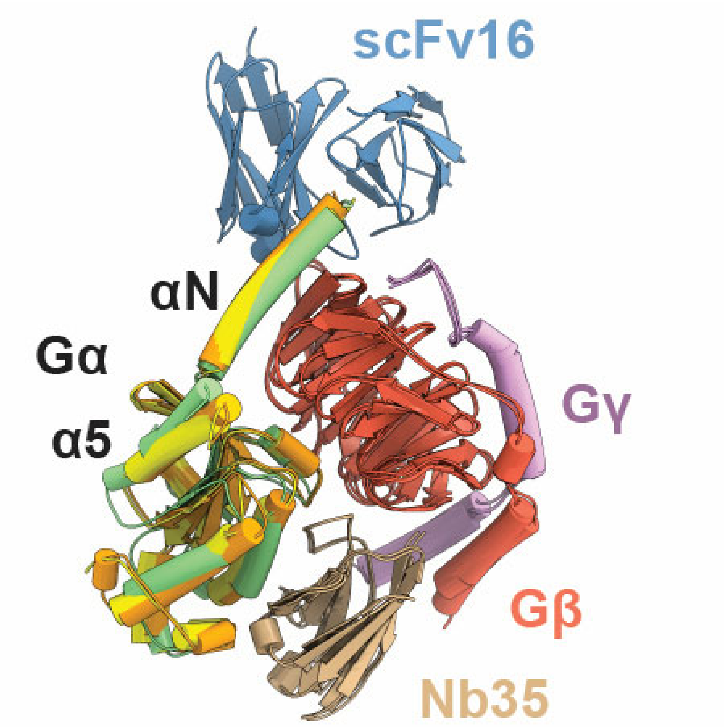

Revision Fig. 1

Structual comparison of miniGs/Gi trimer with Nb35/scFv16. miniGs (yellow from MT1-miniGs and orange from MT2-MT1-miniGs) and Gi (green from MT1-Gi (PDB 7DB6)) were overlayed by Gβ (red). Gγ and Nb35 are well overlayed.

Some very minor points:
1. Should line 307 read "selectivity" or "enabling Gs coupling"? "Dispensible for Gi coupling" to me suggests that ICL3 is not playing any role there at all which it obviously is….
Response: We agree with your suggestions and modified the manuscript accordingly as below: "This is consistent with the observation that chimeric replacement of TM5 alone is not sufficient to enable Gs coupling to MT2 (see Fig. 5b)."

2. Line 195: The jump from the MT results to GPR61 seems a bit of a logical leap. Could the authors please clarify what they mean here.
Response: Thank you for your suggestions, we weakened the conclusion of this topic as below: "These structural insights may reflect the difference in G protein coupling between primary constitutive Gs signaling (of GPR61) and secondary Gs signaling (of MT1, primarily coupling to Gi)"

In addition, we thought structural comparison with GPR61 is enough to discuss the TM3 feature of MT_1-miniG_s and removed the previous Extended Fig.4 including comparison among GPR174-G_s and 5HT4-G_s/G_i.

3. Figure 2i-2k, these are very minor differences and are probably attributable to overall 3D-variability. As these calculations were not performed, I would urge caution to interpret minor changes in structure in a SPA experiment, especially as we have observed in our work that there is a significant 3D-variation in the G-protein trimers.
Response: Thank you for bringing up this point. Our intention was to mention that the miniGs protein in our structure showed the same structural features as the full-length Gs in previous signaling complexes of Gs coupled GPCRs. We moved these figures to Extended Fig. 4a, b.

4. Line 55 seems a pretty bold claim backed by only a single meta analysis. For example, Class B GPCRs all couple to many different G-proteins, while being predominately Gs coupled, and so do many Class A (CCK, Muscarinics, etc…)
Response:
Thank you for your suggestions. To respond to your point, we showed the similarity of conformations between G protein coupling among Gs-/Gi- complexes with the same GPCRs. We have also toned down the claim and revised the conclusion accordingly. We merged sentences into the previous section" MT1 shares a similar Gs entry angle with GPR61 but shows a different binding mode from GPR61" as below and moved the previous Fig. 3f-g to Extended Data Fig. 4a, b. Corresponding to this change, we thought comparing with structures of Gi-/Gq- complexes with the same GPCRs is out of the mainstream of this manuscript and removed the previous Extended Data Fig. 5.

This is reflected in the manuscript in Page7 Line215-229 as follows;

"These structural insights may reflect the difference in G protein coupling between primary constitutive Gs signaling (of GPR61) and secondary Gs signaling (of MT1, primarily coupling to Gi).
We also compared the structures of five GPCRs in complex with both G_s- and G_i-trimers with the MT_1-miniG_s (Extended Data Fig. 4). Among these receptors, MT_1 is unique as the first primarily G_i-coupled GPCR also complexed with G_s. 5HT4-G_s/-G_i displayed conformational differences in the α5 helix (Extended Data Fig.4a), a pattern closely resembling MT_1-G_s/-G_i complexes (Fig.2i). In contrast, other four GPCRs-G_s/-G_i complexes exhibited less pronounced differences in this region (Extended Data Fig.4a,b). These comparisons implied there are several patterns of -G_s/-G_i coupling to the same GPCR, however, identifying common structural features—including those involving the C-terminal regions of Gα—will likely require the accumulation of additional structural data for multiple GPCR–Gα binding modes."

I think the manuscript deserves publication and adds valuable knowledge into the MT1 and MT2 receptor signaling functions.
Matthew Belousoff, Monash Institute of Pharmaceutical Sciences

Reviewer #2 (Remarks to the Author):

The manuscript by Oishi et al. reports that the melatonin MT1 receptor can engage a non-canonical Gs/cAMP signaling pathway in addition to its canonical Gi signaling. The authors show this with in vitro assays (HEK293T, BRET/HTRF, NanoBiT), ex-vivo PT/MBH slice cultures where 16-h melatonin exposure increases cAMP/pCREB, and an in-vivo experiments in which melatonin injection (ZT8) in mice kept under a long-night schedule (8L:16D) increases PT pCREB measured 16 h later (ZT0). The work also includes cryo-EM structures and MD/chimera experiments that identify ICL3/TM3/ICL2 determinants for MT1–Gs coupling.

Response: We sincerely thank Reviewer #2 for the thoughtful and constructive comments. Below we address each point individually.

1) The in-vivo and ex-vivo work used C57BL/6J mice. C57BL/6 strains are well known to be melatonin-deficient (practically absent nocturnal melatonin rhythm), which may critically affect both baseline PT physiology and response to exogenous melatonin. Please justify the choice of C57BL/6J and discuss whether the observed Gs activation at “dawn” requires an intact endogenous melatonin rhythm. Providing key in-vivo and/or ex-vivo experiments in a melatonin-proficient strain such as the C3H melatonin proficient mice would help clarifying this issue.

Response: Thank you for your suggestion. C57BL/6J mice were selected for the following reasons: In the pars tuberalis (PT), thyroid-stimulating hormone (TSH), a so-called “spring hormone,” is synthesized and secreted locally and acts via an autocrine mechanism through its own receptor (TSHR), which is a Gs-coupled GPCR in melatonin proficient animals (Unfried et al., 2009 (PMID 19589858), Endocrinology; Nakao et al., 2008 Nature (PMID 18354476)). Therefore, potential crosstalk between endogenous TSH–TSHR–Gs signaling and MT1–Gs signaling must be considered. To minimize possible interference from endogenous Gs signaling pathways, and to exclude the influence of endogenous melatonin rhythms, we initially chose melatonin-deficient C57BL/6J mice without seasonal TSH responses. Importantly, although C57BL/6J mice lack robust endogenous melatonin production and they maintain MT1 expression and retain functional responsiveness to exogenous melatonin (Ono et al., PNAS 2008).

To answer the valid point of the reviewer, we generated now new data in the melatonin-proficient CBA/N strain. CBA/N mice were entrained under an 8L:16D photoperiod for three weeks. PT tissues were collected 2 hours after lights-on, prepared for slice culture, and pre-incubated for 6 hours. Subsequently, melatonin (10 μM) was added and slices were exposed for 16 hours before protein extraction.

Under these conditions, melatonin exposure significantly increased cAMP levels and pCREB expression (Supplemental Fig. S4d-f). This replicates our initial data obtained in the C57BL/6J strain now in the CBA/N strain. The results indicate that the MT1–Gs response observed in our ex vivo experiments is unlikely to be driven by endogenous melatonin rhythms and instead reflects a receptor-intrinsic signaling shift induced by prolonged melatonin exposure.

We added this information to the materials and methods, results, and discussion.

The following changes have been made in materials and methods section:

Animals

Since PT expressed not only MT_1 but also G_s-coupled thyroid stimulating hormone receptor related with melatonin signaling [39,40], we compared melatonin-deficient C57BL/6J and melatonin-proficient CBA/N mice. Five-week-old male C57BL/6JJmsSlc mice and CBA/NSlc (Japan SLC Inc., Shizuoka, Japan) were purchased and housed in plastic cages (170 W × 240 D × 125 H mm, Clea, Tokyo, Japan) maintained at a constant temperature (23 ± 1°C). Standard rodent diet (MF; Oriental Yeast Co., Ltd., Tokyo, Japan) and autoclave water were provided *ad libitum*. C57BL/6J and CBA/N mice were kept under short day conditions (8-h light (200 lx of fluorescent light) / 16-h dark cycles (8L16D, 1200 light ON, 2000 light OFF). Under 8L16D, melatonin-proficient mice showed prolonged melatonin secretion during dark compared with long day conditions [41]. To address the effects of photoperiod, C57BL/6JJmsSlc mice were kept under a 12-h light / 12-h dark cycles (12L12D, 0800 light ON, 2000 light OFF) for more than 3 weeks.

In the results section:

MT_1 is abundantly expressed in the rodent pituitary PT whereas MT_2 is absent[23,24]. Acute melatonin treatment has been shown to inhibit FSK-stimulated cAMP production in cultured PT cells[25]. To simulate physiologically relevant long-term stimulation conditions, we first established *ex-vivo* cultures from PT of melatonin-deficient C57BL/6 mice kept under short-day conditions (8h light: 16h dark) and stimulated them for 16h with melatonin (Fig. 1g). After assessing the data normality (Supplementary Fig.S3), we found that, under these conditions, melatonin promoted cAMP production (Fig. 1h) and further downstream CREB phosphorylation (Fig. 1i,j; Supplementary Fig. S3,S5), which are conserved in C57BL/6 mice kept under different light conditions (12h light : 12h dark) (Supplementary Fig. S4a-c) and in melatonin-proficient CBA/N mice (Supplementary Fig. S4d-f), indicating a minor contribution of the endogenous melatonin rhythm and circadian timing on the responses.

In the discussion section:

Stimulation of the G_s/cAMP/PKA pathway was observed in our study *ex vivo* and *in vivo* in both melatonin-proficient and -deficient mouse PT expressing endogenous MT_1 regardless of ambient photoperiod, which seems to be independent of internal circadian timing. Previously, melatonin has been shown to have a dual impact on the rhythmic transcription of PT cells [31].

Supplemental Fig. S4d-f

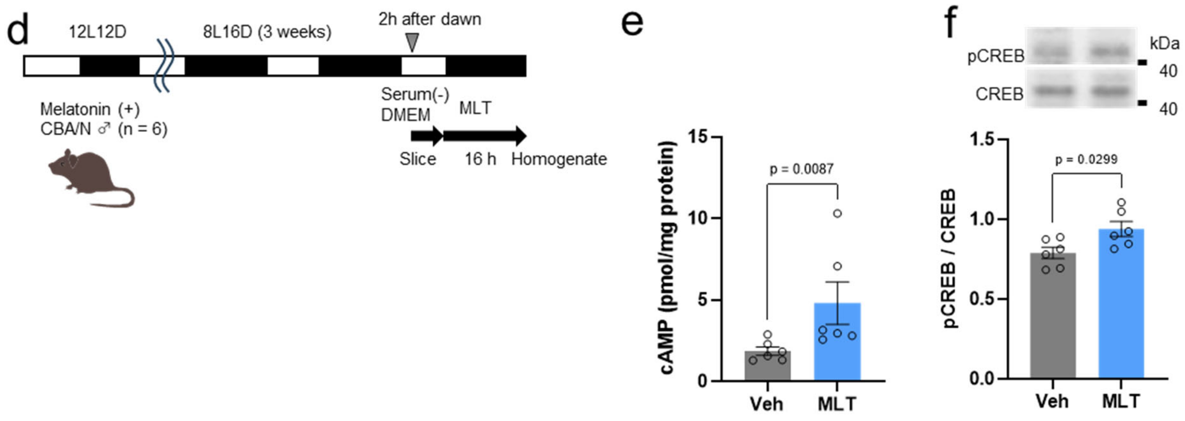

2)In line with the previous comment, given that strain and LD schedule determine endogenous melatonin, reporting serum melatonin levels could better provide a picture of the observed effects.

Response: We agree that reporting serum melatonin levels would provide additional physiological context. Rather than performing additional measurements in the present study, we refer to well-established previous findings.
In our earlier work, we demonstrated that in CBA mice, the expression rhythms of melatonin-synthesizing enzyme genes in the pineal gland vary according to photoperiod (Ikegami et al., PloS One 2012). Furthermore, in melatonin-proficient MSM mice, photoperiodic change alters the duration of elevated melatonin secretion (Kasahara et al., PNAS 2010). Specifically, under a long-day condition (16L8D), melatonin levels show only a transient increase approximately two hours before the end of the dark phase. In contrast, under a short-day condition (8L16D), melatonin secretion begins to rise about three hours after lights-off and remains elevated for a prolonged period throughout the dark phase.
Based on these findings, it is reasonable to infer that melatonin-proficient CBA mice exhibit a similar photoperiod-dependent melatonin rhythm. Therefore, in the additional ex vivo experiments described in response to Comment 1, PT slices were obtained from CBA mice entrained under a physiologically extended melatonin secretion profile.
We have added this clarification to the materials and methods to better contextualize the physiological relevance of our findings.

[REDACTED]

(Kasahara et al., PNAS 2010)

In the materials and methods section:

C57BL/6J and CBA/N mice were kept under short day conditions (8-h light (200 lx of fluorescent light) / 16-h dark cycles (8L16D, 1200 light ON, 2000 light OFF). Under 8L16D, melatonin-proficient mice showed prolonged melatonin secretion during dark compared with long day conditions [41].

3) In the Methods the authors state that slices for ex-vivo were from mice maintained 12L:12D and treated at ZT12 for 16 h in culture, whereas in-vivo animals were kept under 8L:16D for 3 weeks and received melatonin at ZT8, with tissue collection after 16 h (ZT0). Please explicitly justify the switch from 12L:12D (ex-vivo source) to 8L:16D (in-vivo) — what physiological condition is modelled by 8L:16D? In keeping, is any dynamic Gi-Gs switch expected according to timing and melatonin physiological levels? The authors have included a single time point. At least a comment should be provided.

Response: We sincerely thank the reviewer for this important and constructive comment. We agree that in our original design, the use of 12L12D conditions for the ex vivo experiments was not explicitly linked to a defined physiological model.

In response to this suggestion, we performed additional ex vivo experiments under short-day conditions (8L16D), which model a winter-like photoperiod. Using C57BL/6J mice entrained to 8L16D, we examined the effect of 16-hour melatonin exposure. Under these short-day conditions, melatonin increased cAMP and pCREB levels. In addition, experiments using melatonin-proficient CBA mice were also performed under short-day conditions, and this is now explicitly described in the revised manuscript. Thus, all ex vivo experiments are now consistently conducted under a defined short-day physiological model.

These results indicate that prolonged melatonin exposure induces -Gi-to-Gs signaling shift at the MT1 receptor. However, whether such switching occurs dynamically within a physiological circadian context remains unknown. We have therefore added a statement in the limitations section clarifying that:

- Although the switching appears to be relatively insensitive to endogenous melatonin rhythms (as discussed in our response to Comment 1),
- We currently assessed only a single time point per day,
- Therefore, time-of-day dependency cannot be excluded.

We state in the Discussion that future studies will address the temporal dynamics of this signaling shift.

In materials and methods

Animals

Since PT expressed not only MT_1 but also G_s-coupled thyroid stimulating hormone receptor related with melatonin signaling[39,40], we compared melatonin-deficient C57BL/6J and melatonin-proficient CBA/N mice. Five-week-old male C57BL/6JJmsSlc mice and CBA/NSlc (Japan SLC Inc., Shizuoka, Japan) were purchased and housed in plastic cages (170 W × 240 D × 125 H mm, Clea, Tokyo, Japan) maintained at a constant

temperature (23 ± 1°C). Standard rodent diet (MF; Oriental Yeast Co., Ltd., Tokyo, Japan) and autoclave water were provided *ad libitum*. C57BL/6J and CBA/N mice were kept under short day conditions (8-h light (200 lx of fluorescent light) / 16-h dark cycles (8L16D, 1200 light ON, 2000 light OFF). Under 8L16D, melatonin-proficient mice showed prolonged melatonin secretion during dark compared with long day conditions [41]. To address the effects of photoperiod, C57BL/6JJmsSlc mice were kept under a 12-h light / 12-h dark cycles (12L12D, 0800 light ON, 2000 light OFF) for more than 3 weeks.

Ex- and *In-Vivo* Melatonin treatment

For analysis of melatonin effect on cultured PT/MBH, brain of 8–12-week-old mice were quickly removed under general anesthesia at 6 hours after dawn in 12L12D and at 2 hours after dawn in short day conditions (8L16D), and then immersed in ice-cold Hank's buffered saline (HBS; #09735-75, Nacalai tesque, Kyoto, Japan). The hypothalamus, containing the PT and MBH was sectioned at 1 mm thickness by brain matrix. Then, the PT/MBHs were placed on a culture membrane (Millicell-CM PICM0RG50, Merck Millipore, Darmstadt, Germany) in a 35-mm petri dish and cultured at 37°C with 1.2 mL of medium containing Dulbecco's modified Eagle medium (DMEM) (Gibco, Carlsbad, CA), 100 U/mL penicillin, and 100 µg/mL streptomycin. After 6 hours preincubation, slices were then added with melatonin (finally 10 µM, 0.1 % DMSO; Wako, Tokyo, Japan) in C57BL/6J mice, and in CBA/N mice (finally 10 µM) at light offset. After 16 hours, proteins of the PT/MBH were extracted for using cAMP measurement and western blotting.
For pharmacological inhibitory experiment, brain of 8-week-old mice were quickly removed under general anesthesia 2 hours after dawn in 8L16D. After slice and 5 hours preincubation, slices were then added with MT1/2 antagonist luzindole (Luz, 30 µM) for 1 hour, and then with melatonin (finally 10 µM) for 16 hours.

In results

MT_1 is abundantly expressed in the rodent pituitary PT whereas MT_2 is absent[23,24]. Acute melatonin treatment has been shown to inhibit FSK-stimulated cAMP production in cultured PT cells[25]. To simulate physiologically relevant long-term stimulation conditions, we first established *ex-vivo* cultures from PT of melatonin-deficient C57BL/6 mice kept under short-day conditions (8h light: 16h dark) and stimulated them for 16h with melatonin (Fig. 1g). After assessing the data normality (Supplementary Fig.S3), we found that, under these conditions, melatonin promoted cAMP production (Fig. 1h) and further downstream CREB phosphorylation (Fig. 1i,j; Supplementary Fig. S3,S5), which are conserved in C57BL/6 mice kept under different light conditions (12h light : 12h dark) (Supplementary Fig. S4a-c) and in melatonin-proficient CBA/N mice (Supplementary Fig. S4d-f), indicating a minor contribution of the endogenous melatonin rhythm and circadian timing on the responses.

In discussion

Stimulation of the G_s/cAMP/PKA pathway was observed in our study *ex vivo* and *in vivo* in both melatonin-proficient and -deficient mouse PT expressing endogenous MT_1

regardless of ambient photoperiod, which seems to be independent of internal circadian timing. Previously, melatonin has been shown to have a dual impact on the rhythmic transcription of PT cells [31].

Several limitations of this study should be acknowledged. First, *in vivo* signaling was assessed at a single melatonin dose and at a single time point, leaving the minimal melatonin dose necessary and potential time-of-day–dependent dynamics of MT_1 G_i–G_s switching open questions. Second, analyses focused primarily on the PT, and broader hypothalamic network effects, including the SCN, were not examined. Finally, behavioral or physiological alterations affected by the observed signaling shift should be determined in future studies.

Figure 1g-l

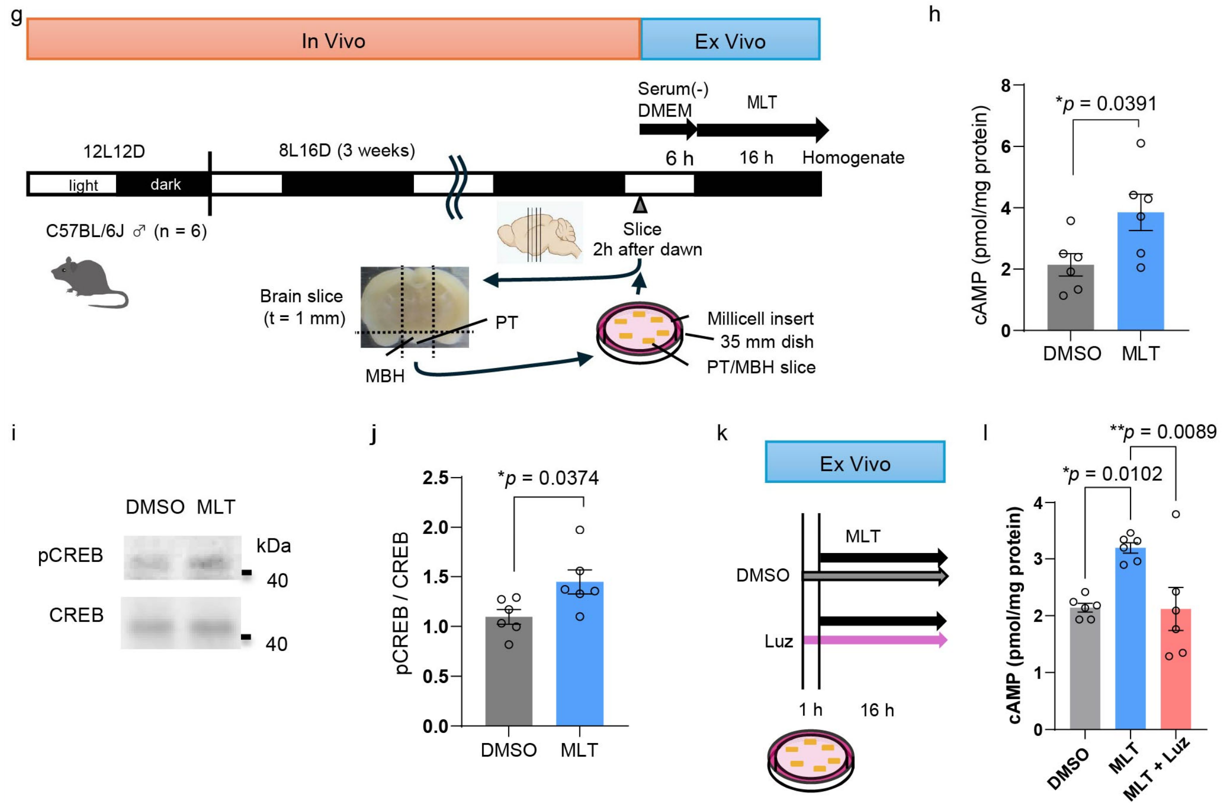

Finally, the sequence of time points used in ex vivo and in vivo experiments (ZT definitions, 6 h post-dawn collection, 16-h incubation, etc.) is scientifically reasonable but not intuitive for non-chronobiologists. I suggest a simple figure highliting light–dark cycles, melatonin exposure, and tissue collection times to greatly improve clarity for non-specialized readership.

Response: We fully agree that the temporal design was not intuitive for non-chronobiologists. To improve clarity:

- We removed ZT notation and unified timing relative to lights-on.

- We added a schematic figure (Fig. 1, Supplemental Fig.S4) showing light–dark cycles as black/white bars.
- Experimental schedules, including tissue collection times and melatonin administration timing, are indicated directly on the timeline.

We believe these revisions substantially improve the clarity and physiological interpretation of the study.

Figure 1g

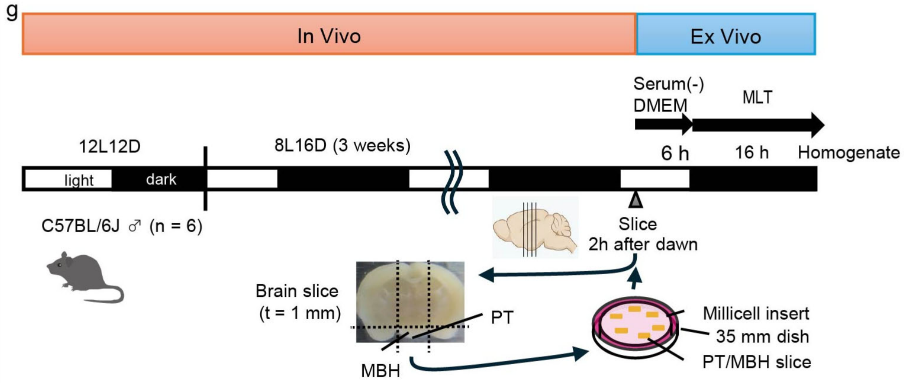

Supplemental Figure S4a

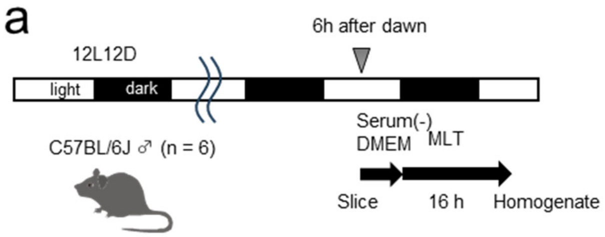

Supplemental Figure S4d

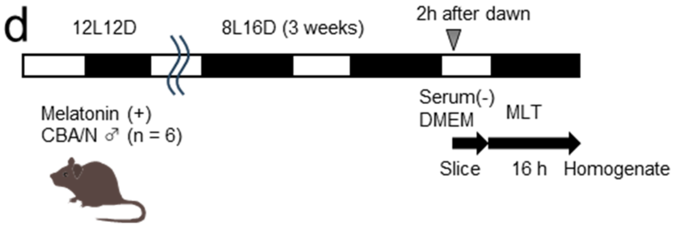

4) one key finding is the cAMP/pCREB increase to MT1–Gs coupling in vivo. I would suggest one of the following experiments to prove this finding: use of a selective MT1 antagonist or MT1 KO (or MT1/MT2 double KO) control; demonstration that the signal is abolished by PKA inhibition although very selective compounds are relatively missing; or inclusion of MT1 receptor expression/localization data in PT under the experimental LD conditions of the study.

Response: We appreciate the reviewer's suggestion to strengthen the evidence supporting MT1–Gs coupling *in vivo*.

Although the use of MT1 knockout mice would have been ideal, we were unfortunately unable to obtain MT1 KO mice or a well-validated MT1-specific antibodies within the revision period. Therefore, we employed a pharmacological approach in our *ex vivo* system by using the luzindole antagonist. In the absence of any well-characterized MT1 selective antagonist for in vivo studies, as pointed out repeatedly in the literature, we used the non-selective antagonist luzindole which blocks both MT1 and MT2. The aim was to verify whether the melatonin-induced cAMP increase can be blocked at the receptor level. The rational of using luzindole was motivated by the fact that it is the most extensively characterized melatonin receptor antagonist and the fact that no functional MT2 is expressed in the PT, as reported in previous studies (Yasuo et al. 2009, PMID: 19261884, Ref24). Luzindole treatment attenuated the melatonin-induced elevation of cAMP (Fig. 1l) which is most likely due to MT1 receptor blockade.
Taken together, these findings indicate that the melatonin-induced activation of the cAMP/pCREB pathway in our system is receptor-dependent and consistent with MT1–Gs coupling. Although genetic confirmation using MT1 KO models remains an important future direction, our pharmacological data provide convergent evidence supporting the conclusion that the in vivo cAMP pathway activation is driven by MT1–Gs signaling.
We have incorporated these results and their interpretation into the revised manuscript.

In results

To confirm the importance of MT1 in these responses, we applied the MT1/2 antagonist luzindole, which suppressed, as expected, the melatonin-induced cAMP elevation (Fig. 1k,l).

In materials and methods

For pharmacological inhibitory experiment, brains of 8-week-old mice were quickly removed under general anesthesia 2 hours after dawn in 8L16D. After slice and 5 hours preincubation, slices were then added with MT1/2 antagonist luzindole (Luz, 30 μM) for 1 hour, and then with melatonin (finally 10 μM) for 16 hours.

Figure 1k,l

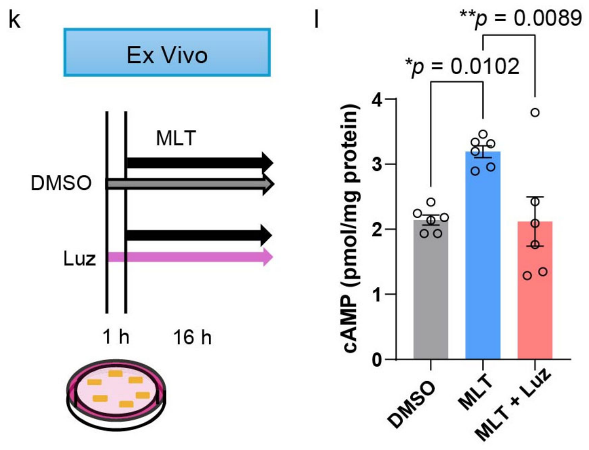

5) I suggest reporting exactly the n (biological replicates) for each panel in the figures (some panels report n=4 or n=6–7 but be explicit for each experiment). Consider adding power justification for the in-vivo measures.

Response: We thank the reviewer for this important suggestion.
In the revised manuscript, we now explicitly report the exact biological replicate number (n) for each panel in the figure legends. The previously reported n = 4 corresponded to ex vivo experiments performed under 12L:12D conditions, which have now been removed. Therefore, these data and their associated sample sizes have been deleted.
For the in vivo experiments, n = 6–7 animals per group is now clearly stated in each figure legend. The newly added ex vivo experiments under short-day conditions were conducted with n = 6 animals per group, and this is also explicitly indicated.
Sample sizes for the in vivo experiments (n = 6–7 per group) were chosen based on prior experience with this experimental system and on effect sizes observed in preliminary studies, in which melatonin-induced changes in pCREB were consistently robust. For example, in in vivo experiment, the observed effect size was large (η^2 = 0.51), and with these group sizes the statistical power is estimated to be approximately ~0.8 at α = 0.05, indicating that the sample size was sufficient to detect biologically meaningful differences (Supplemental Fig. S3b).
All figure legends have been revised to include exact n, statistical test used, degrees of freedom, test statistics (t or F values), exact p-values, and confirmation that normality was assessed using the Shapiro–Wilk test. We have included these details along with my response to Comment 6.
We believe these revisions improve transparency and statistical rigor.

In materials and methods

Animals

Sample sizes (Veh; n =6, MLT; n = 7)) for in vivo experiments were selected based on prior experience with similar studies and pilot data. The observed effect size was large (η^2 = 0.51), and group sizes of n = 6–7 provide approximately 80% power at a two-sided α = 0.05 (Supplemental Fig.S3).

Revised Figure 1 legend
(g-l) Melatonin induces cAMP production in mouse pituitary *pars tuberalis* (PT) *ex vivo*. (g) Melatonin (10 μM) were exposed to sliced PT / mediobasal hypothalamus (MBH) tissue complex of C57BL/6J mice (n = 6) kept under 8L/16D conditions.

(o) Relative changes of pCREB-ir cells normalized by area in the PT. Melatonin injection increased pCREB (***p* = 0.0183 [t = 2.936, df = 8.206], two-tailed unpaired t-test with Welch's correction). Data are means ± SEM with scatter plot (Veh; n =6, MLT; n = 7). Normality was assessed by Shapiro–Wilk (Supplementary Fig. S3; all *p* > 0.05).

Supplemental Figure S3b

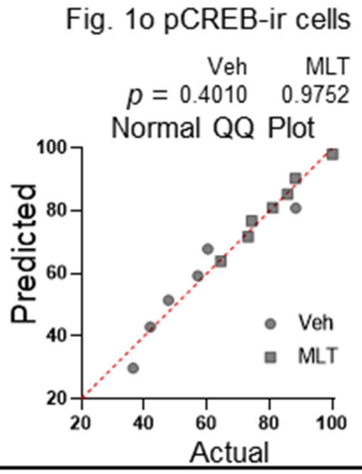

Effect size $\eta^2 = 0.51$
Power ≈ 80% at a two-sided $\alpha = 0.05$.

6) Statistics: The authors report one-tailed p-values for some ex-vivo and in-vivo comparisons (e.g., pCREB analyses). One-tailed tests are acceptable only with strong a-priori directional hypotheses and should be justified. MOreover, t-tests, ANOVAs, Holm–Šidák and Bonferroni corrections have been computed. Please ensure each figure legend explicitly lists the test used, exact n, degrees of freedom (or F/t values), and whether normality was tested (as exmaple using Shapiro–Wilk given the low n per group) and variance equality assessed. Finally, provide exact p-values and corrected p-values consistently.

Response: We thank the reviewer for this important comment. We agree that the statistical reporting in the original version was insufficiently detailed and that the use of one-tailed tests was not adequately justified.
In the revised manuscript, all statistical analyses have been re-evaluated and standardized as follows:

1. **Two-tailed tests** are now used throughout the manuscript. One-tailed tests have been removed to ensure a more conservative and transparent statistical approach.
2. **Normality testing:**
   The Shapiro–Wilk test was performed for all groups prior to parametric analysis. In addition, QQ plots were generated for each dataset to visually assess distribution normality. For datasets expressed as fold of basal (Fig. 1c–f, Fig. 4d, and Fig. 5f,g), log-normality was assessed, consistent with the use of ratio-paired t-tests. For datasets normalized to the 0 melatonin condition as 100%, normality was assessed on the distributions of within-sample changes relative to the reference condition, because the normalized reference values were fixed and therefore not suitable for direct normality testing. These Shapiro–Wilk p-values and QQ plots are now included in Supplementary Fig. S3.
3. **Two-group comparisons (Fig 1h, 1j, 1o):**
   Welch's t-test was used as the default approach to account for potential unequal

variances. In cases where normality was violated (e.g., the CBA melatonin-treated group (Supplementary Fig.S4d-f)), non-parametric Mann–Whitney U tests were applied instead.

4. **One-way ANOVA analyses (Fig.1l):**
   Ordinary one-way ANOVA was used when assumptions of normality and homogeneity of variance were met. Homogeneity of variance was assessed using Bartlett's test.
5. **Multiple comparisons (Fig.1l):**
   For Fig. 1l, statistical analysis was performed using ordinary one-way ANOVA followed by Dunnett's multiple comparisons test, and exact adjusted p-values are reported.
6. **In vitro paired and repeated-measures analyses (Fig. 1c–f, Fig. 4d, and Fig. 5b-g, Fig.6c-d):**
   For the in vitro experiments, ratio-paired t-tests[*1] were used for Fig. 1c–f, Fig. 4d, and Fig. 5f,g; paired multiple t-tests[*2] with Holm–Šidák correction were used for Fig. 2h; and repeated-measures one-way ANOVA with Geisser–Greenhouse correction followed by Bonferroni's multiple-comparisons test was used for Fig. 5b–e and Fig. 6c,d. For paired analyses (Fig.1c-f,2h,4d,5f,g), equality of variances was not assumed. For repeated-measures ANOVA (Fig.5b-e, 6c,d), sphericity was not assumed and was addressed using the Geisser–Greenhouse correction.
   [*1] For Fig. 1c–f, Fig. 4d, and Fig. 5f,g, the statistical analysis was corrected from paired t-tests to ratio-paired t-tests in the revised manuscript, because these datasets were evaluated on a log scale and ratio-paired analysis was considered more appropriate. Although the statistic conclusions were unchanged, the exact p-values shown in the figures were updated. This is noted here for transparency.
   [*2] For Fig. 2h, the statistical analysis was corrected from unpaired to paired multiple t-tests with Holm–Šidák correction in the revised manuscript. This change reflects the repeated-measures design used in the experiment. Although the significance conclusions were unchanged, the exact p-values shown in the figure were updated. This is noted here for transparency.

7. **Statistical reporting transparency:**
   All figure legends and/or Results sections now explicitly include:
   - exact n (biological replicates),
   - statistical test used,
   - degrees of freedom,
   - test statistics (t or F values),
   - exact p-values,
   - and confirmation that normality was assessed using the Shapiro–Wilk test.

We believe these revisions substantially improve statistical rigor, transparency, and reproducibility.

These modifications are presented as follows:

In materials and methods

Statistical analysis

All data are presented as the mean ± standard error of the mean (SEM). Normality was assessed by Shapiro–Wilk (Supplementary Fig. S3; all $p > 0.05$). For paired analyses (ratio-paired t-tests and paired multiple t-tests), equality of variances was not assumed. For repeated-measures one-way ANOVA, sphericity was not assumed and the Geisser–Greenhouse correction was applied. For unpaired comparisons in ex vivo and in vivo experiments, Welch's correction was applied; therefore, equality of variances was not assumed.

Revised Figure legend

Fig 1. Melatonin MT_1 receptor activates the stimulatory G_s/cAMP pathway *in vitro*, *ex vivo* and *in vivo*

(a-f) Melatonin MT_1 receptor activated G_s-cAMP pathway in HEK293T cells. (a-b) Concentration–response effect of melatonin (MLT) on forskolin (FSK)-induced cAMP production (30 min, FSK [2 μM], IBMX [1 mM]) in HEK293T cells expressing MT_1-WT or MT_2-WT, assessed by HTRF assay without (a) or with (b) PTX treatment (400 ng/mL, 4 h). Data represent means ± SEM of at least 4 independent experiments. (c-f) NanoBiT complementation assay showing recruitment of LgBiT–miniG_i (c,e) or LgBiT–miniG_s (d,f) to human MT_1-NP (c, d) or MT_2-NP (e, f) upon melatonin stimulation (1 μM, 2 min). Data are presented as mean ± SEM from five independent experiments (n=5). Statistical significance was determined using two-tailed ratio-paired t-tests; exact p-values are reported. Log-normality was assessed by Shapiro–Wilk (Supplementary Fig. S3; all $p > 0.05$). MT_1-G_i (c); [t=30.82, df=4], MT_1-G_s (d); [t=5.074, df=4], MT_2-G_i (e); [t=30.53, df=4], MT_2-G_s (f); [t=2.064, df=4].

(g-l) Melatonin induces cAMP production in mouse pituitary *pars tuberalis* (PT) *ex vivo*. (g) Melatonin (10 μM) were exposed to sliced PT / mediobasal hypothalamus (MBH) tissue complex of C57BL/6J mice (n = 6) kept under 8L/16D conditions. (h) Sixteen-hour exposure of melatonin significantly increased cAMP levels in the PT/MBH (** $p = 0.0391$ [t=2.448, df=8.273], two-tailed unpaired t-test with Welch's correction), and phosphorylated CREB (pCREB [Ser133]) (** $p = 0.0374$ [t = 2.475, df = 8.307], two-tailed unpaired t-test with Welch's correction). Data are means ± SEM with scatter plot. Normality was assessed by Shapiro–Wilk (Supplementary Fig. S3; all $p > 0.05$). (k,l) One hour preincubation with $MT_{1/2}$ antagonist luzindole (Luz) prevented the MLT-induced cAMP elevation (** $p = 0.0089$ vs MLT with Dunnet's multiple comparison test, ** $p = 0.0066$ with ordinary one-way ANOVA [F (2, 15) = 7.162]). Normality was assessed by Shapiro–Wilk (Supplementary Fig. S3; all $p > 0.05$).

(m-o) Melatonin activates CREB in mouse pituitary PT *in vivo*. (m) MLT (0.26 mM / 0.1 mL) was administered intraperitoneally (i.p.) at light offset under 8L16D, and brains were collected 16 h later for histological analyses. (n) Representative images of pCREB immunoreactivity (ir) in the PT and MBH of Veh and MLT-injected mice. 3V, Third ventricle. (o) Relative changes of pCREB-ir cells normalized by area in the PT. Melatonin injection increased pCREB (**$p = 0.0183$ [t = 2.936, df = 8.206], two-tailed unpaired t-test with Welch's correction). Data are means ± SEM with scatter plot (Veh; n =6, MLT; n = 7).

Normality was assessed by Shapiro–Wilk (Supplementary Fig. S3; all $p > 0.05$).

Fig. 2 Cryo-EM structure of the MT_1-miniG$_s$ complex

(a) CryoEM density map of MT_1-miniG$_s$ with Gβ_1, Gγ_2 and Nb35. MT_1, miniG$_s$, Gβ_1, Gγ_2 and Nb35 were shown as blue, yellow, red, pink and brown, respectively. Map including micelle density was shown in transparent style.
(b) Overall structure of MT_1-miniG$_s$ with Gβ_1, Gγ_2 and Nb35. Melatonin molecule is shown as light purple CPK model.
(c) The interface between MT_1 and miniG$_s$.
(d–g) Concentration–response effect of melatonin (MLT) on FSK-induced cAMP production (5 min, FSK (5μM) in HEK293T cells expressing MT1-WT (d) or mutant receptors Y128A (e), S132A (f), and L133A (g), monitored with the BRET-based CAMYEL biosensor. Data represent means ± SEM from six independent experiments.
(h) Melatonin (1 μM, 10 min)–induced cAMP production in the absence of FSK in HEK293T cells pretreated with PTX (400 ng/mL, 4 h) and expressing MT1-WT or mutant receptors (Y128A, S132A, L133A) assessed with the BRET-based CAMYEL biosensor. Normality was assessed by Shapiro–Wilk (Supplementary Fig. S3; all $p > 0.05$). Data represent means ± SEM from four independent experiments ($n = 4$). Multiple paired t-tests with Holm–Šidák correction were used for statistical comparison, exact adjusted p-values are indicated. WT; [t=11.17, df=3], Y128A; [t=0.2833, df=3], S132A; [t=9.050, df=3], L133A; [t=2.023, df=3].
(i-k) Structural comparison with MT_1-miniG$_s$ (light blue and purple; PDB 7DB6), displaying (i) overall structure, (j) ICL2 and (k) TM3.

Fig. 4 Structural basis of MT_2-MT_1-chimera G$_s$ coupling

(a) Amino acid sequences alignment of human MT_1 and MT_2 chimeric receptors in TM5-ICL3-TM6 region.
(b,c) Concentration–response effect of melatonin on FSK-induced cAMP production (5 min, FSK [5μM]) in HEK293T cells expressing (b) MT_1–MT_2(TM5–ICL3–TM6) or (c) MT_2–MT_1(TM5–ICL3–TM6) chimeric receptors, assessed with the BRET-based CAMYEL biosensor. Data represent means ± SEM of six independent experiments.
(d) NanoBiT complementation assay showing recruitment of LgBiT–miniG$_i$ (left) or LgBiT–miniG$_s$ (right) to the MT_2–MT_1(TM5–ICL3–TM6)–NP receptor upon melatonin stimulation (1 μM, 2 min). Data present means ± SEM of five independent experiments ($n = 5$). Log-normality was assessed by Shapiro–Wilk (Supplementary Fig. S3; all $p > 0.05$). Statistical significance was determined using ratio-paired two-tailed t-tests, exact p-values are reported. mGi; [t=16.32, df=4], mGs; [t=23.31, df=4].
(e) CryoEM density map (left) and overall structure (right) of MT_2-MT_1(TM5-TM6)-miniG$_s$ with Gβ_1, Gγ_2 and Nb35. MT_2, MT_1(TM5-TM6), miniG$_s$, Gβ_1, Gγ_2 and Nb35 were shown as light green, blue, orange, red, pink and brown, respectively. Map including micelle density was shown in transparent style.
(f, g) Structural comparison of MT_2-MT_1(TM5-TM6)-miniG$_s$ with (f) MT_1-miniG$_s$ or (g) MT_2-G$_i$ (PDB 7VH0), including lateral and cytoplasmic viewpoints. MT_2 in MT_2-G$_i$ is shown as light purple, and G$_i$ in MT_2-G$_i$ is shown as green.
(h) The interface between MT_2-MT_1(TM5-TM6) and miniG$_s$ around TM3 and ICL2.

(i, j) Structural comparison between MT_2-MT_1(TM5-TM6)-miniG_s and MT_1-miniG_s at (i) TM3 and α5 helix or (j) ICL2.

(k) Structural comparison between MT_2-MT_1(TM5-TM6)-miniG_s and MT_2-G_i at TM5, TM6 and α5 helix.

Fig. 5 Identification of key determinants of MT_1-G_s interaction by MT_2-MT_1 chimera receptors

(a) Amino acid sequences alignment of human MT_2 chimeric receptors.

(b-e) (upper) Schematic representation of chimera. (middle) Concentration–response effect of melatonin on FSK-induced cAMP production (10 min, FSK [5μM]) in HEK293T cells expressing (b) MT2–MT1(TM5c), (c) MT2–MT1(TM6), (d) MT2–MT1(ICL3) or (e) MT2–MT1(core ICL3) chimeric receptors, assessed with the BRET-based CAMYEL biosensor. Data represent means ± SEM of seven independent experiments (n = 7). (lower) Statistical comparison of concentration–response data at 0, 1 nM and 1 μM of melatonin. Normality was assessed by Shapiro–Wilk (Supplementary Fig. S3; all $p > 0.05$). Statistical analysis was performed by repeated-measures one-way ANOVA with Geisser–Greenhouse correction followed by Bonferroni's multiple-comparisons test. F (DFn, DFd): F (1.704, 10.22) =197.5 (b), F (1.624, 9.744) = 163.1 (c), F (1.630, 9.781) = 56.05 (d) and F (1.861, 11.17) = 23.91 (e). Exact adjusted *p*-values are shown on the plots.

(f, g) NanoBiT complementation assay showing recruitment of LgBiT–miniGi (f) or LgBiT–miniGs (g) to the MT_2–MT_1(coreICL3)–NP receptor upon melatonin stimulation (1 μM, 2 min). Log-normality was assessed by Shapiro–Wilk (Supplementary Fig. S3; all $p > 0.05$). Data represent means ± SEM of five independent experiments. Statistical significance was determined using ratio-paired two-tailed t-tests, exact *p*-values are reported. mGi (f); [t=38.50, df=4], mGs(g); [t=13.92, df=4].

Fig. 6 Molecular dynamics simulations support differential G protein binding modes and ICL3-dependent G_s coupling of MT_1

(a) Representative MD simulation snapshots of MT_1-G protein complexes. Carbon colours indicate electrostatic energies, stick thickness represents van der Waals interactions with α5 helix. Only residues >1 kcal/mol in TM5-ICL3-TM6 are shown. AlphaFold3-modeled ICL3 (pink) highlights non-conserved hydrophobic residues. TM5 and TM6 are highlighted in darker colour. Key contacts: $I210^{5.61}$ with $L348^{G.H5.20}$, $F354^{GH5.26}$, and $L353^{GH5.25}$ (G_i deep binding) versus $V217^{ICL3}$ with $L388^{G.H5.20}$ (G_s shallow binding).

(b) Amino acid sequence alignment of human MT_2 chimera and human MT_1 deletion mutant in TM5-ICL3 region.

(c,d) (upper) Concentration–response effect of melatonin on FSK-induced cAMP production (10 min, FSK [5μM]) in HEK293T cells expressing (c) MT2-MT1(coreICL3-V217A) and (d) MT1-ICL3-deletion mutant chimeric receptors, assessed with the BRET-based CAMYEL biosensor. Data represent means ± SEM of seven (c) or four (d) independent experiments (n = 7 (c), n = 4 (d)). (Lower) Statistical comparison of concentration–response data (Upper) at 0, 1 nM and 1 µM of melatonin. Normality was assessed by Shapiro–Wilk (Supplementary Fig. S3; all $p > 0.05$). Statistical analysis was performed by repeated-measures one-way ANOVA with Geisser–Greenhouse correction followed by Bonferroni's multiple-comparisons test. F (DFn, DFd): F (1.128, 6.768) = 156.7 (c) and F (1.732, 5.197) = 46.10 (d). Exact adjusted *p*-values are shown on the plots.

In supplemental figures with legend

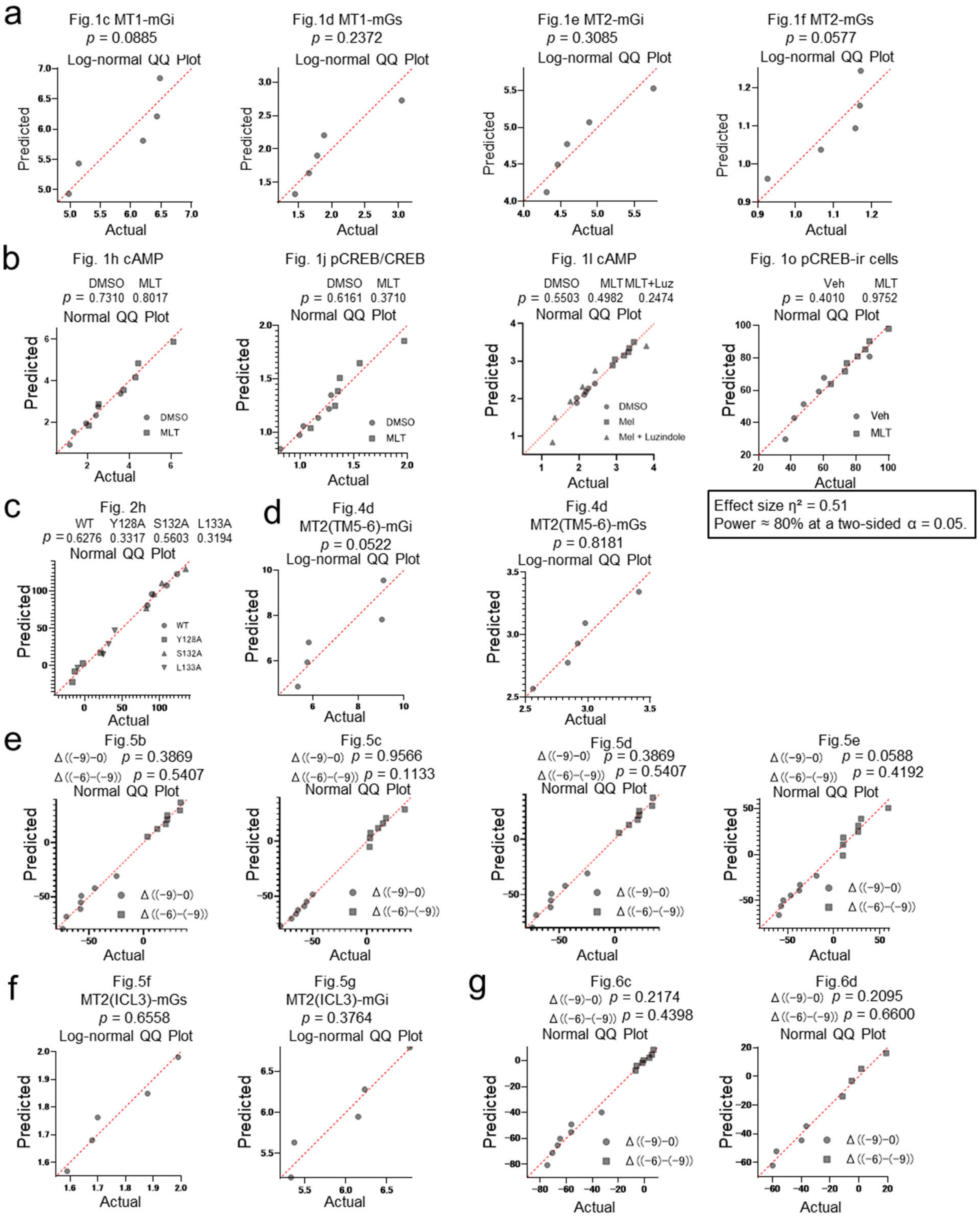

Supplementary Fig. S3 | Normal and log-normal Q–Q plots for assessment of distributional assumptions. Normality assumptions were evaluated using the Shapiro–Wilk test, and the corresponding Q–Q plots are shown for each dataset used in the main statistical analyses. Panels are arranged to match the order of the corresponding main-figure datasets. For fold-change (ratio) datasets (baseline/vehicle-normalized), analyses were performed on the log scale, and log-normal Q–Q plots of the log-transformed values (log ratios) are shown. For datasets normalized to 0 concentration (set to 100%), normality was assessed on the within-subject differences (Δ) relevant to the planned comparisons, and normal Q–Q plots of Δ are shown. Shapiro–Wilk p-values are indicated within each panel; all datasets satisfied the normality criteria ($p > 0.05$). For Fig. 1o, the observed effect size was large ($\eta^2 = 0.51$), and group sizes of $n = 6–7$ provide approximately 80% power at a two-sided $\alpha = 0.05$ (b).

7) The limitations of the study should be better adressed.

Response: Thank you for this important suggestion. We agree that the limitations of the study were not sufficiently articulated in the original version.
In the revised manuscript, we have substantially expanded the Limitations section to provide a more balanced and transparent interpretation of our findings.
We believe that these additions clarify the scope of the study and appropriately define their boundaries as below:

In discussion section:
Several limitations of this study should be acknowledged. First, *in vivo* signaling was assessed at a single melatonin dose and at a single time point, leaving the minimal melatonin dose necessary and potential time-of-day–dependent dynamics of MT_1 G_i–G_s switching open questions. Second, analyses focused primarily on the PT, and broader hypothalamic network effects, including the SCN, were not examined. Finally, behavioral or physiological alterations affected by the observed signaling shift should be determined in future studies.

8) The manuscript should be largely revised for typos, grammar and incorrect phrasing. The abbreviations should also be used consistently and cited at the first time of appearence.

Response: We have changed that the original manuscript required substantial revision for language clarity, grammatical accuracy, and consistency of terminology. In the revised version, the manuscript has been carefully edited throughout to correct typographical errors, improve grammar, and refine phrasing for clarity and precision. We have also ensured that all abbreviations are defined at first appearance and used consistently thereafter. In addition, terminology related to MT1–Gi/Gs signaling has been harmonized across all sections of the manuscript to avoid ambiguity.

Reviewer #3 (Remarks to the Author):

I co-reviewed this manuscript with one of the reviewers who provided the listed reports. This is part of the Nature Communications initiative to facilitate training in peer review and to provide appropriate recognition for Early Career Researchers who co-review manuscripts.

Reviewer #4 (Remarks to the Author):

A very interesting article, worthy of publishing in Nature Communications, on the mechanism and importance of the coupling of the melatonin MT1 receptor to both Gi and Gs. The authors have combined pharmacology, structural biology and computational chemistry in a very effective synergy and have shed some interesting

light on the difficult issue of G protein selectivity.
Where it says ‘The physiological relevance of dual Gi/Gs coupling… remains elusive” is a very elegant phrase but it is probably an overstatement that should be toned down, e.g. Weston et al., JBC (2015, 2016) showed how RAMPs affected GPCR Gs/Gi coupling in a ligand dependant manner.
The statement that “with no universal structural determinant emerging from current structures, highlighting the complexity of GPCR-G protein selectivity” (p7, line 213)is a little depressing if we are looking for simple answers, but it is very important and the authors shed considerable light on this topic in this article, e.g. with regard to the proposed (by others) importance of the TM5-TM6 distance (p7, line 22). The most interesting aspects of this work lie in the excellent work on MT1-MT2 chimeras (including a cryo-EM structure), which point to the importance of ICL3. There is a very long history on the importance of ICL3 in G protein coupling that should be noted, but I think this is significant and well-validated.
The modelling of ICL3 is discussed on p9. It is refered to as a short loop – but at 20 residues that is not the case. I would accept relatively short rather than short - its still in the region difficult to model and I'm not convinced we can fully trust AlphaFold in this type of modelling.

Response: We thank the reviewer for this valid point. We agree that describing ICL3 as "short" at 20 residues is imprecise, and we have revised the text to "relatively short" as suggested. The AlphaFold model aligns closely with our cryo-EM structures (**Figure R1**). The first five amino acids of ICL3 (VRQRV in MT1; ARRKA in MT2) are resolved experimentally as an extended TM5 helix, which AlphaFold models similarly. The pLDDT scores for ICL3 (65.17 for MT1; 73.62 for MT2) fall within the low-to-moderate confidence range, reflecting inherent loop flexibility. Importantly, V217, which our mutagenesis identified as specifically required for Gs coupling is resolved in the cryo-EM structures at the base of TM5.

We acknowledge that modelling flexible regions remains challenging. To avoid bias toward the initial AlphaFold conformation, we extended our simulations by performing both conventional MD and Gaussian accelerated MD (GaMD) simulations. PCA of the resulting trajectories confirms that ICL3 samples substantially larger conformational space than the starting structure (**Figure R2**). Crucially, our conclusions are based on primarily on experimental chimera and mutagenesis studies (Figures 5 and 6), with MD simulations providing mechanistic insight into these observations.

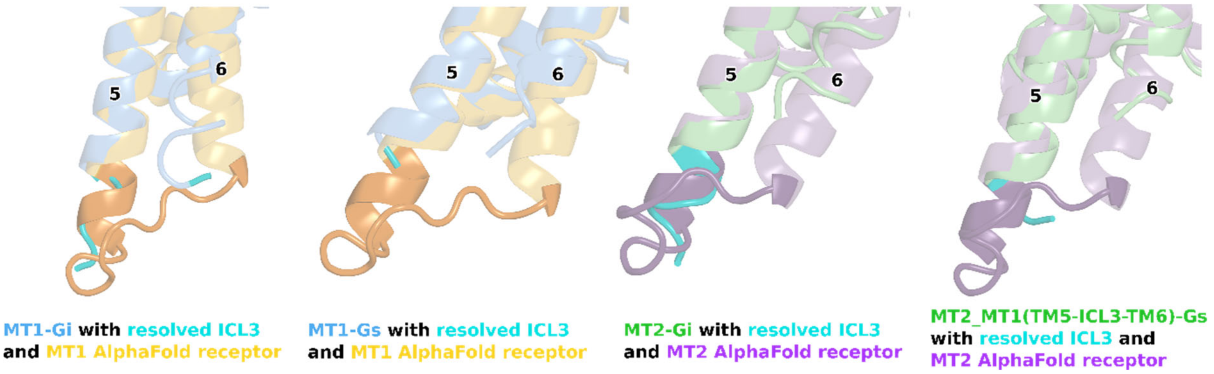

Figure R1: Comparison of AlphaFold-predicted ICL3 conformations with cryo-EM resolved structures of the melatonin receptor-G protein complexes. Overlay of AlphaFold models with corresponding cryo-EM structures for MT1-Gi (PDB: 7DB6), MT1-Gs, MT2-Gi (PDB: 7VH0), and MT2-MT1(TM5-ICL3-TM6)-Gs complexes. The cryo-EM resolved receptor structures are shown in blue (MT1) and light green (MT2), with the experimentally resolved portions of ICL3 highlighted in bright cyan. AlphaFold-modelled ICL3 regions are shown in dark orange (MT1) and purple (MT2). TM5 and TM6 are labelled for orientation. The AlphaFold ICL3 conformations show reasonable agreement with the resolved portions of the cryo-EM structures, supporting their use in molecular dynamics simulations to investigate ICL3-G protein interactions.

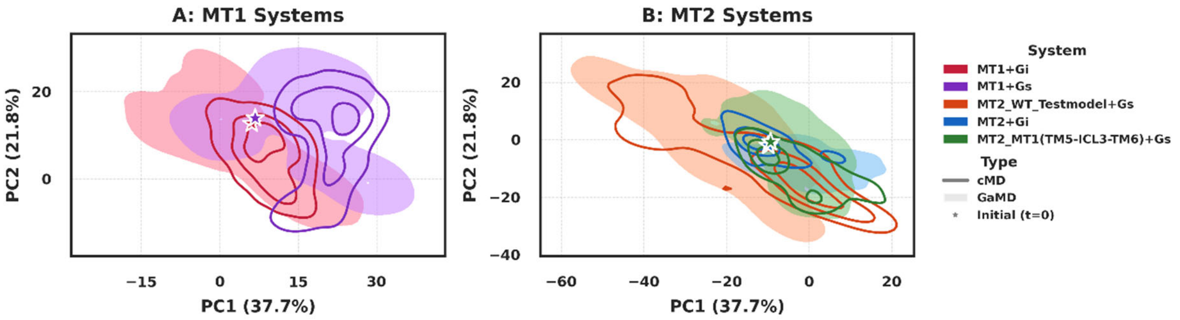

Figure R2. Principal Component Analysis (PCA) reveals distinct ICL3 conformational ensembles sampled during conventional MD (cMD) and Gaussian accelerated MD (GaMD) simulations across melatonin receptor-G protein complexes.

Generally the modelling is excellent, and provides valuable supporting information. However, I have concerns about modelling the interaction energies. NAMDenergy is simply a molecular mechanics energy and as such does not take into account entropy, which is important for systems such as this that involve hydrophobic interactions, and so the current calculations are not acceptable - the work should be repeated, e.g. with

entropy taken into account with a surface area term, e.g. as in MMGBSA.

Response: We thank the reviewer for this constructive suggestion. We agree that molecular mechanics energies alone do not adequately account for entropic contributions, particularly for hydrophobic interactions at the ICL3-Gα interface. Following the reviewer's recommendation, we have repeated the calculations using MM/GBSA, which incorporates a surface area term to capture hydrophobic contributions to binding (**Figure R3**). The analysis was performed on both cMD (n = 5) and GaMD (n = 3) trajectories. The results support our original conclusions: the MT2-WT test model displays substantially weaker Gs binding compared to MT1-Gs and chimera-Gs complexes, consistent with the inability of wild-type MT2 to stably couple to Gs. We have updated the manuscript accordingly.

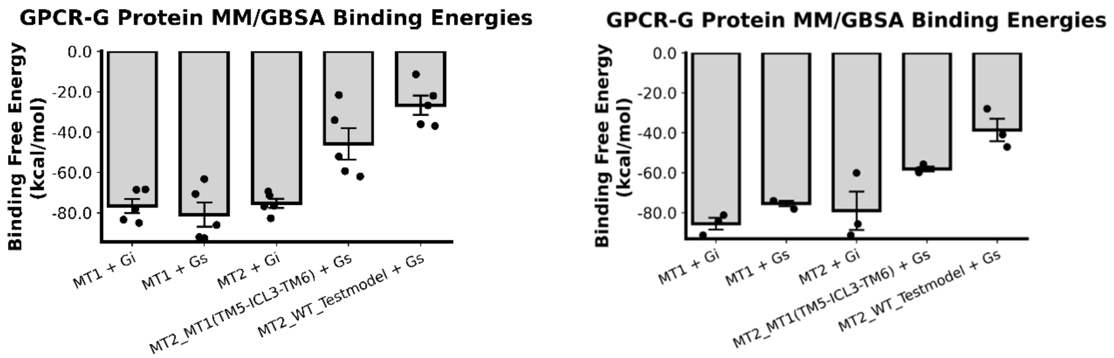

Figure R3: Receptor-Gα binding energies from cMD (left) and GaMD (right) simulations. MM/GBSA analysis reveals weaker Gs binding for the MT2-WT test model compared to MT1-Gs and chimera-Gs complexes, supporting the inability of wild-type MT2 to couple to Gs. Mean ± SEM; cMD: n = 5, GaMD: n = 3.

Minor comments

P2 line 55. Rarely co-couple to Gi/o proteins or CO-COUPLE with a large…. Would add clarity

Response: Thank you for this comment. To clarify, we added the representative examples as following **"**G_s-coupled GPCRs show the lowest coupling promiscuity and rarely co-couple to $G_{i/o}$ proteins (e.g.V2R) or with a large difference in their strength of activation (e.g.β1AR)[1]"

P6 line 187 "I" should be "is"

Response: Thank you for this comment. We corrected here "is".

P7 line 213. The authors point out the possible problems of using engineered G proteins. Can the authors confirm that their G proteins were not engineered and so there are no hidden problems arising from that?
Response: Thank you for your suggestion, yes in short. When we submitted, we considered structural comparison with β1AR is critical in the main logic flow but now toned down responding to the point from Reviewer1. We thought that Gi protein in the structure of β1AR-Gi was a chimeric mini Gi/s construct swapped most of its part by Gs protein except for C-term wavy hook (only 5 amino acids from Gi), which plays a main role in G protein selectivity. In parallel, we used the miniG version of Gs protein, which is not chimeric but pure miniGs, and this miniGs construct is used for plenty of structural biology studies in GPCR field, reflecting our consensus that using miniGs construct doesn’t cause the hidden artifacts.

P13 line 427. no need for space before degrees C - it's not an SI unit
Response: Thank you for this comment. We removed the space before “°C”.
